# Sequence-based drug design as a concept in computational drug design

**DOI:** 10.1038/s41467-023-39856-w

**Published:** 2023-07-14

**Authors:** Lifan Chen, Zisheng Fan, Jie Chang, Ruirui Yang, Hui Hou, Hao Guo, Yinghui Zhang, Tianbiao Yang, Chenmao Zhou, Qibang Sui, Zhengyang Chen, Chen Zheng, Xinyue Hao, Keke Zhang, Rongrong Cui, Zehong Zhang, Hudson Ma, Yiluan Ding, Naixia Zhang, Xiaojie Lu, Xiaomin Luo, Hualiang Jiang, Sulin Zhang, Mingyue Zheng

**Affiliations:** 1 Drug Discovery and Design Center, State Key Laboratory of Drug Research, Shanghai Institute of Materia Medica, Chinese Academy of Sciences, 555 Zuchongzhi Road, Shanghai, 201203 China; 2 University of Chinese Academy of Sciences, No. 19A Yuquan Road, Beijing, 100049 China; 3 School of Chinese Materia Medica, Nanjing University of Chinese Medicine, 138 Xianlin Road, Jiangsu Nanjing, 210023 China; 4 Shanghai Institute for Advanced Immunochemical Studies and School of Life Science and Technology, ShanghaiTech University, No. 393 Huaxia Middle Road, Shanghai, 200031 China; 5 Department of Analytical Chemistry, State Key Laboratory of Drug Research, Shanghai Institute of Materia Medica, Chinese Academy of Sciences, 555 Zuchongzhi Road, Shanghai, 201203 China; 6 School of Pharmaceutical Science and Technology, Hangzhou Institute for Advanced Study, University of Chinese Academy of Sciences, 1 Sub-lane Xiangshan, Hangzhou, 310024 China

**Keywords:** Drug screening, Target identification, Virtual drug screening, Computational models

## Abstract

Drug development based on target proteins has been a successful approach in recent decades. However, the conventional structure-based drug design (SBDD) pipeline is a complex, human-engineered process with multiple independently optimized steps. Here, we propose a sequence-to-drug concept for computational drug design based on protein sequence information by end-to-end differentiable learning. We validate this concept in three stages. First, we design TransformerCPI2.0 as a core tool for the concept, which demonstrates generalization ability across proteins and compounds. Second, we interpret the binding knowledge that TransformerCPI2.0 learned. Finally, we use TransformerCPI2.0 to discover new hits for challenging drug targets, and identify new target for an existing drug based on an inverse application of the concept. Overall, this proof-of-concept study shows that the sequence-to-drug concept adds a perspective on drug design. It can serve as an alternative method to SBDD, particularly for proteins that do not yet have high-quality 3D structures available.

## Abstract (web summary)

Conventional structure-based drug design pipeline is a complex, human-engineered process with multiple independently optimized steps. Here, the authors report a sequence-to-drug concept that discovers drug-like small molecule modulators directly from protein sequences.

## Introduction

**Fig. 1:**
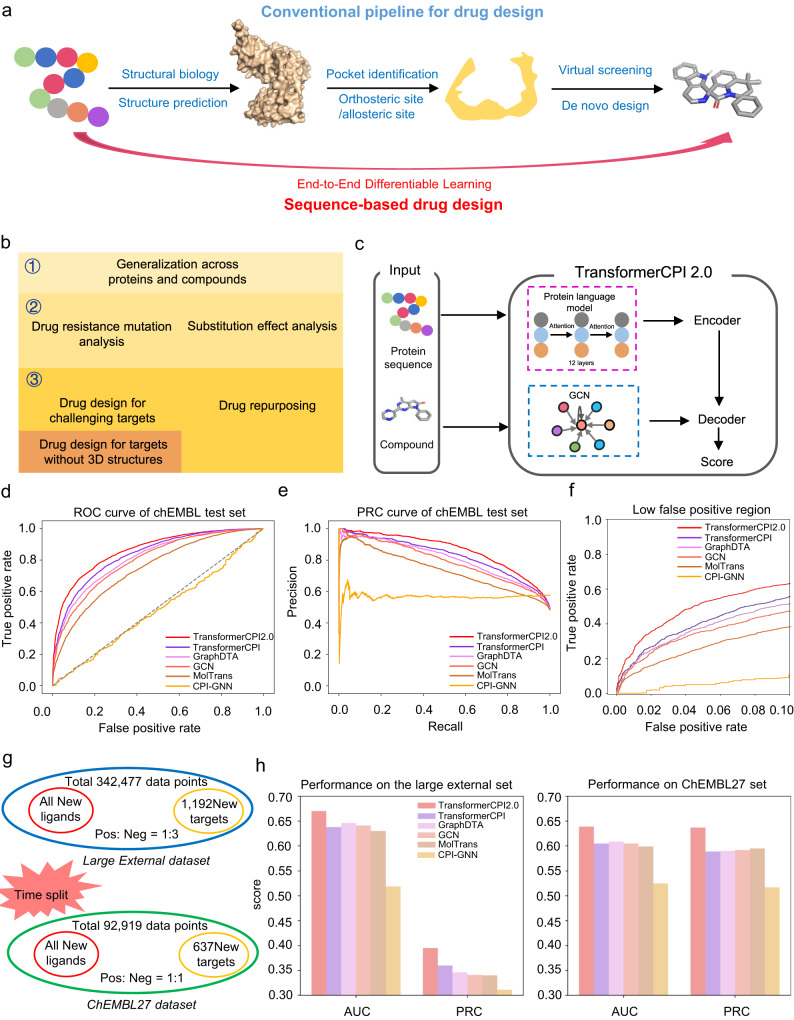
TransformerCPI2.0: predicting compound protein interaction without using protein structure. **a** The conventional pipeline for target-based drug design and the sequence-to-drug concept. **b** Three stages of the proof of sequence-to-drug concept, with each stage is labeled by different colors. First, we examined the generalization ability across proteins and chemical space. Second, we designed drug resistance mutation analysis and substitution effect analysis to interpret our model whether it learns knowledge as expected. Third, we applied a sequence-to-drug concept to screen new hits for challenging targets and novel targets without 3D structures, and conducted drug repurposing task. **c** The computational pipeline of TransformerCPI2.0. **d** AUC curves of TransformerCPI2.0 and baseline models on the ChEMBL set. **e** PRC curves of TransformerCPI2.0 and baseline models on the ChEMBL set. **f** ROC curves in low-false-positive region. **g** The arrangement of the external dataset and ChEMBL27 dataset. **h** The performance of TransformerCPI2.0 and baseline models on the external set and ChEMBL27 set.

Protein structure-based drug development has been a successful approach for diseases with well-defined protein targets over the past few decades^1–3^. A typical protein structure-based drug design (SBDD) project starts from the protein sequence and builds a three-dimensional (3D) structure through structural biology or structure prediction. It then identifies binding pockets, including orthosteric sites or allosteric sites, and finally discovers active modulators through virtual screening or de novo design^4,5^ (Fig. 1a). This process involves a complex, human-engineered pipeline with multiple independently optimized steps, and each step has its own limitations^5^. For example, many proteins do not have high-resolution structures, and while recent advances in protein structure prediction such as AlphaFold^6^ and RoseTTAFold^7^ have been successful, not all predicted structures are suitable for SBDD^8,9^, given that only 36% of all residues have very high confidence^10^. In particular, the precise predicting active sites remains a challenge as these local structures tend to break the ‘protein-folding rules’^9^. Another challenge is defining binding pockets for novel targets with multiple domains^11^, and predicting allosteric sites is still difficult^12^ due to the varied mechanisms of allosteric effects and high computational costs^13^. Additionally, structural flexibility allows proteins to adapt to their individual molecular binders and undergo different internal motions^9,14,15^, making pockets more difficult to define. Finally, virtual screening can generate false positives^16^ and accumulate errors from the previous two steps.

Here, we propose a sequence-to-drug concept that discovers modulators directly from protein sequences without intermediate steps, using end-to-end differentiable learning (Fig. 1a). End-to-end differentiable deep learning has revolutionized computer vision and speech recognition^17^ by replacing all components of complex pipelines with differentiable primitives, enabling joint optimization from input to output^18^. The success of AlphaFold^6^ in protein structure prediction also relies heavily on the idea of end-to-end differentiability. This concept is appealing because it performs the entire learning process in a self-consistent and data-efficient manner, potentially avoiding the error accumulation of complex pipelines.

Several deep learning models have been proposed to use protein sequences as input^19–28^. However, none have thoroughly verified the concept of the sequence-to-drug paradigm. In this work, we address the issue in three stages (Fig. 1b). First, we designed TransformerCPI2.0 as a fundamental tool of the sequence-to-drug paradigm, which exhibited generalization ability across proteins and chemical space. Second, we used case studies to interpret our model to verify whether it learns knowledge as expected, rather than exhibiting only data bias^20^. Third, we applied TransformerCPI2.0 to discover new hits for challenging targets, speckle-type POZ protein (SPOP) and ring finger protein 130 (RNF130), which lacks existing 3D structures. Additionally, we identified ADP-ribosylation factor 1 (ARF1) as a new target for proton pump inhibitors (PPIs). After the proof of concept, the sequence-to-drug concept appears to be a promising direction for rational drug design.

## Results

### TransformerCPI2.0: predicting compound protein interaction without using protein structure

To build a model that can implement the sequence-to-drug concept, we developed TransformerCPI2.0 based on our previous work^20^ and its framework is shown in Fig. 1c. As pointed out in our previous work, there is a common hidden ligand bias issue in existing CPI datasets^20^. Therefore, we ensured that each compound in our dataset exists in positive class and negative class, but pairs with different proteins. Because the compounds with positive and negative labels are exactly the same, ligand bias is greatly reduced in our dataset. Under this criteria, we constructed a ChEMBL dataset containing 217,732 samples in the training set, 24,193 samples in the validation set, and 10,199 in the test set. Consequently, we used the label reversal experiment^20^ to split the ChEMBL dataset, where some chosen ligands in the training set appear only in one class of samples (either positive or negative interaction CPI pairs), but in the opposite class of samples in the test set. If a model only memorizes the ligand patterns, it is unlikely to make correct predictions because the ligands it memorizes have the wrong (opposite) labels in the test set. Within the scheme of label reversal experiments, the model was forced to utilize protein information along with compound information to understand interaction patterns and thus overcome the ligand bias issue.

TransformerCPI^20^, CPI-GNN^19^, GraphDTA(GAT-GCN)^21^, MolTrans^29^ and Graph Convolutional Networks (GCN)^21^ were selected as baseline models, and all were retrained on the ChEMBL dataset. We trained TransformerCPI2.0 and baseline models under the same criteria and compared their performance in terms of area under the Receiver Operating Characteristic Curve (AUC) and area under the Precision Recall Curve (PRC) (Fig. 1d–f). TransformerCPI2.0 achieves the best performance among all models. In addition, we tested TransformerCPI2.0 and the baseline models on the other two external datasets: a large external set containing new proteins and molecules, and a time-split test set named the ChEMBL27 dataset containing the new data that were deposited online after the training set (Fig. 1g). TransformerCPI2.0 also showed the greatest generalization ability among all models (Fig. 1h and Supplementary Tables 1 and 2). The large external set supported that TransformerCPI2.0 can generalize to previously unseen proteins and molecules. Since our training set was generated from ChEMBL23, this time-split test suggested that our model can learn from past knowledge and generalize to future data. Overall, TransformerCPI2.0 is worthwhile to be applied to virtual screening and target identification tasks.

To confirm the feasibility of sequence-to-drug concept, we compared TransformerCPI2.0 with conventional structure-based drug design approaches to test its ability to screen active molecules from compound libraries. We used the benchmark dataset DUD-E set^30^ and DEKOIS2.0 set^31^ and the enrichment factor (EF0.5%, EF1%, EF5%) for the screen power assessment^32^, which is calculated from the proportion of true active compounds in the selection set in relation to the proportion of true active compounds in the entire dataset (at a sampling ratio of 0.5%, 1% and 5%, respectively).

From Supplementary Table 3, we may find that TransformerCPI2.0 has comparable screening ability to the structure-based docking models, which is inferior to the commercial program CCDC’s GOLD^33^, but slightly higher than the academic program AutoDock Vina^34^. From Supplementary Table 4, we may find that the screening ability of TransformerCPI2.0 is slightly higher than GOLD and AutoDock Vina. This result is encouraging because it demonstrates that sequence-to-drug models can achieve virtual screening performance close to structure-based methods (but without relying on any prior knowledge about the 3D structure of proteins), and it also verifies the feasibility of applying the concept for drug discovery.

### Interpretation of TransformerCPI2.0 by two analysis tools

To investigate whether TransformerCPI2.0 captures correct information about binding sites, we proposed an analysis method named drug resistance mutation analysis that mimics alanine scanning^35^. Briefly, we mutated each amino acid of the given protein sequence one by one and examined whether the prediction score changed significantly. We input the wild-type protein and drug into TransformerCPI2.0 to calculate the original prediction score, denoted as *s*. Then we mutated each amino acid of the protein sequence to all 20 amino acids (including itself) and calculated the prediction score *s’*. The activity change score Δ**S** is defined as the difference between *s* and *s*′ Then the relative activity change score (Δ**R**) is defined as the average of Δ**S** among 20 amino acids at each position, followed by normalization.

**Fig. 2:**
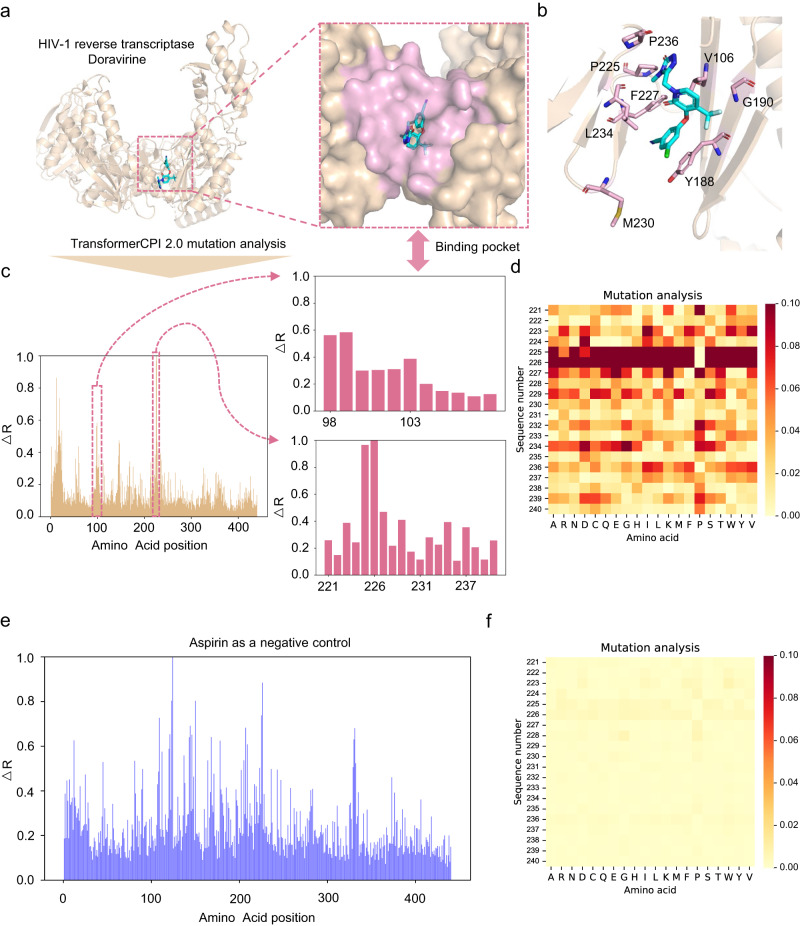
Drug resistance mutation analysis. **a** The cocrystal structure of HIV-1 reverse transcriptase and doravirine (PDB: 4NCG). The binding pocket of doravirine is highlighted in pink. **b** The binding mode of doravirine. The residues with drug-resistant mutations are colored pink. **c** Relative activity change score (Δ**R**) calculated by TransformerCPI2.0 at each amino acid position. The pink boxes mark the high Δ**R** regions, which are plotted in detail on the right. **d** The heatmap plots the activity change score of positions 221–240, where each position is mutated to each of the 20 amino acids (including itself). The darker color represents the higher activity change score caused by the mutation. **e** Relative activity change score (Δ**R**) calculated by TransformerCPI2.0 for each amino acid position. The pink boxes mark the high Δ**R** regions, which are plotted in detail on the right. **f** The heatmap plots the activity change score Δ**S** of positions 221–240, where each position is mutated to the 20 different amino acids (including itself). The darker color represents the higher activity change score caused by the mutation.

We selected HIV-1 reverse transcriptase and its inhibitor doravirine as an example (PDB: 4NCG, Fig. 2a). Doravirine (formerly MK-1439) has been approved by the FDA for the treatment of HIV-infected, treatment-naive individuals in combination with other antiretroviral drugs^36^. It is encouraging that positions with a high Δ**R** are highly overlapped with the binding sites of doravirine (Fig. 2a–c), since neither structural nor binding pocket information is included in the training phase. There is a region with a high Δ**R** but irrelevant to binding sites, possibly because this region is disordered in the 3D structure (PDB: 4NCG). We considered positions with Δ**R** above 0.38 as important sites corresponding to the top 5% sites of the entire sequence. As a result, P225, F227, L234 and P236 have been reported as drug resistance mutation sites^37–40^ and are correctly retrieved as important sites by TransformerCPI2.0 (Fig. 2b, c). Some predictions matched the reported drug resistance mutations, such as P225H, F227C/L/R and P236L (Fig. 2d). Position 226 has not been reported as a drug resistance mutation site, although it has a high Δ**R** predicted by TransformerCPI2.0. Position 226 may be just a false positive prediction, given that TransformerCPI2.0 still has limitations and cannot provide completely correct predictions, or that the mutation does cause resistance, but it has not been observed to be abundant in the patient population. Another reasonable concern is that the model might learn from protein sequence alone rather than protein–ligand interactions. We selected aspirin as a negative control (Fig. 2e, f) and found that the pattern of Δ**R** was significantly different from that of doravirine.

**Fig. 3:**
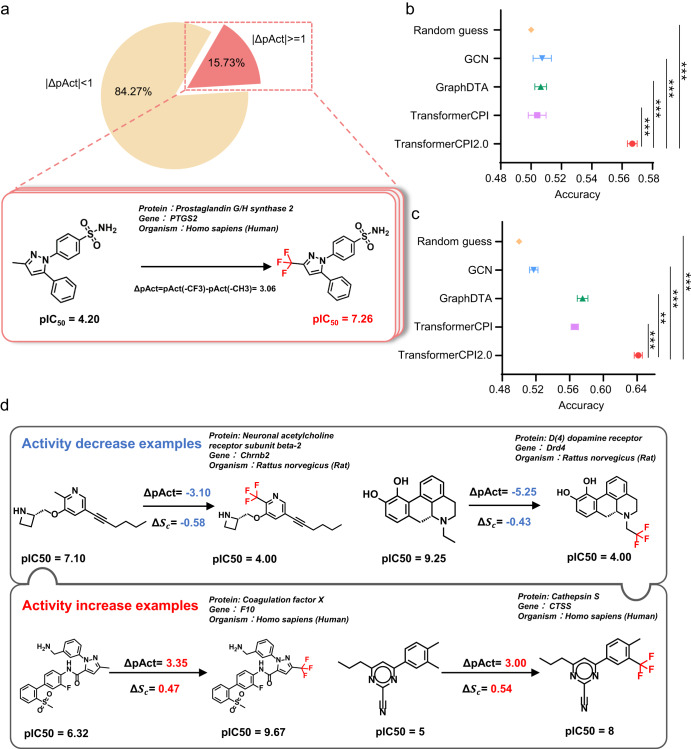
Substitution effect analysis of the trifluoromethyl group. **a** Left, data distribution of the trifluoromethyl substitution dataset. Only 15.73% of substitution of −CH_3_ by −CF_3_ could increase or decrease the biological activity by at least an order of magnitude. We conducted substitution effect analysis on this part of data. Right, an example of −CH_3_ changed by −CF_3_ leads to a significant increase in biological activity. **b** The overall accuracy of TransformerCPI2.0 and baseline models on the whole dataset. Error bars represent mean ± SEM of three independent experiments. *P* values were evaluated using 2-tailed unpaired *t*-test, ***P < 0.001. (TranformerCPI2.0 vs. TransformerCPI, *P* = 0.0007; TranformerCPI2.0 vs. GraphDTA, *P* = 0.0003; TranformerCPI2.0 vs. GCN, *P* = 0.0009; TranformerCPI2.0 vs. Random Guess, *P* < 0.0001.) **c** The overall accura**c**y of TransformerCPI2.0 and baseline models on the subset where the substitution of −CH_3_ by −CF_3_ could increase or decrease the biological activity by at least three orders of magnitude. Error bars represent mean ± SEM of three independent experiments. *P* values were evaluated using 2-tailed unpaired t-test, **P < 0.01,***P < 0.001. (TranformerCPI2.0 vs. TransformerCPI, *P* = 0.0003; TranformerCPI2.0 vs. GraphDTA, *P* = 0.0012; TranformerCPI2.0 vs. GCN, *P* < 0.0001; TranformerCPI2.0 vs. Random Guess, *P* < 0.0001.) **d** Additional two activity decrease examples and activity increase examples are shown. The predictions of TransformerCPI2.0 are consistent with the ground truth for proteins and compounds not present in the training set.

To interpret whether TransformerCPI2.0 captures activity-related information from compounds, we designed a substitution effect analysis of the trifluoromethyl group as an example. Activity cliffs are generally understood as pairs or groups of similar compounds with large differences in potency^41,42^. Recently, Abula et al.^43^. proposed a dataset including compound pairs and corresponding bioactivity data, with the only difference that −CH_3_ is replaced by −CF_3_, which was not overlapped with our training set after data cleaning. Only 15.73% of the substitutions of −CF_3_ for −CH_3_ could increase or decrease the biological activity by at least one order of magnitude, and an example is shown (Fig. 3a). We computed the activity change score ($\Delta {s}_{c}$) and conducted substitution effect analysis on this part of the data. $\Delta {s}_{c}$ is defined as the difference in activity between trifluoromethyl substituents and methyl substituents, which describes the effects of chemical groups.

TransformerCPI2.0 reveals higher consistency with ground truth than baseline models (Fig. 3b and Supplementary Table 5). In addition, we evaluated the performance of TransformerCPI2.0 and baseline models on a subset where −CH_3_ to −CF_3_ substitution could increase or decrease biological activity by at least three orders of magnitude. TransformerCPI2.0 still outperformed the baselines (Fig. 3c and Supplementary Table 6). This test is more challenging than the whole dataset because the drastic change in biological activity in this range involves the conversion of an active compound into an inactive one or vice versa. At last, we showed some illustrative examples (Fig. 3d) that subtle structural differences produce drastic changes in activity, when none of the protein targets and compounds were included in the training set. These results indicated that TransformerCPI2.0 can capture more useful information about compounds than baselines when training on the same dataset.

Here we have introduced two analysis tools to help users interpret the prediction result of TransformerCPI2.0 and assess the confidence of predictions based on whether binding sites are retrieved correctly or structure-activity relations agree with known knowledge. We emphasize that these two analysis methods serve only as interpretation tools, and the systematical evaluation of the prediction of binding sites and activity cliffs is beyond the scope of this work.

### Drug design targeting E3 ubiquitin-protein ligases

SPOP functions as an adapter of cullin3-RING ubiquitin ligase, mediates substrate protein recognition and ubiquitination^44,45^. Previous studies have validated SPOP as an attractive target for the treatment of clear-cell renal cell carcinoma (ccRCC) and reported the first SPOP inhibitor, but it is a challenging target in terms of protein–protein interactions^46^. In ccRCC cells, SPOP is overexpressed and misallocated in the cytoplasm, inducing proliferation and promoting renal tumorigenesis^47^. Two substrates of SPOP are phosphatase and tensin homolog (PTEN) and dual specificity phosphatase 7 (DUSP7)^47^. PTEN acts as a negative regulator of phosphoinositide 3-kinase/AKT pathway, and DUSP7 dephosphorylates extracellular signal-regulated kinase (ERK)^48^. The accumulation of cytoplasmic SPOP in ccRCC cells decreases cellular PTEN and DUSP7 by mediating the degradation of these two cytoplasmic proteins, leading to an increase in phosphorylated AKT and ERK and promoting ccRCC cell proliferation^47^.

**Fig. 4:**
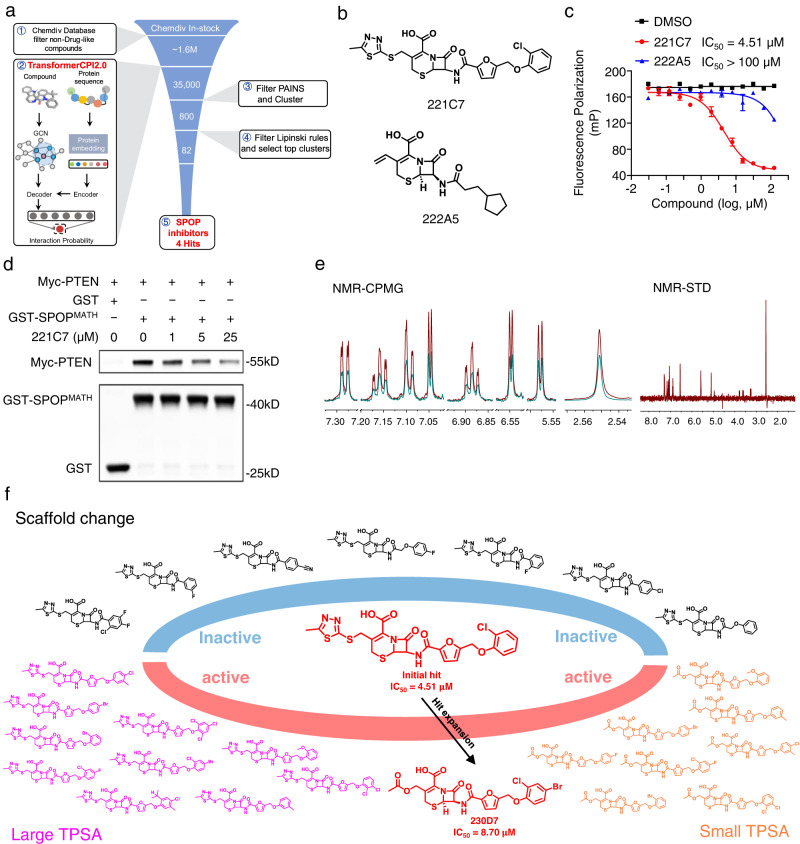
Discovering a novel scaffold hit of SPOP. **a** Scheme of virtual screening protocol for small-molecule inhibitors of SPOP. **b** Chemical structure of 221C7 and its negative control 222A5. **c** 221C7 competitively inhibits puc_SBC1 peptide binding to SPOP^MATH^, as measured by the FP assay. Negative control 222A5 does not inhibit puc_SBC1 peptide binding to SPOP^MATH^. Error bars represent mean ± SEM of two independent experiments. **d** 221C7 disrupts protein binding between SPOP^MATH^ and PTEN, as measured by in vitro pull-down assay. This experiment is repeated three times independently with similar results. **e** NMR measurement of direct binding between 221C7 and SPOP^MATH^. CPMG NMR spectra for 221C7 (red), 221C7 in the presence of 5 ${{{{{\rm{\mu }}}}}}$M SPOP^MATH^ (green). The STD spectrum for 221C7 is recorded in the presence of 5 ${{{{{\rm{\mu }}}}}}$M SPOP^MATH^. **f** A similarity search of 221C7 was conducted, and 26 compounds were purchased, 19 of which were active in the FP assay. Source data are provided as a Source Data file.

Since SPOP is a challenging target and not included in the training set of TransformerCPI2.0, SPOP is suitable to test the generalization of the sequence-to-drug concept to a new target. Here, a virtual screening with TransformerCPI2.0 was performed to discover new scaffold compounds that directly target SPOP (Fig. 4a, Supplementary Table 7). Four compounds were identified as initial hits by a fluorescence polarization (FP) assay (hit rate ~5%), and 221C7 was the most active compound with an IC_50_ of 4.51 μM (Fig. 4b, c, Supplementary Fig. 1a, b).

Compared with other tools, 221C7 was highly ranked and discovered only by TransformerCPI2.0 (Supplementary Table 8). Furthermore, these four hits revealed low similarity with the scaffold of known active compounds (Supplementary Table 9), indicating that TransformerCPI2.0 does not conduct a similarity search. We also revisited the training set to ensure that 221C7 was not screened only by compound similarity. The compounds in the training set have low similarity with 221C7 (Supplementary Fig. 1c), and the most similar compound (containing β-lactam ring) targets a different protein with very low sequence identity with SPOP (Supplementary Fig. 1d). Therefore, TransformerCPI2.0 does not replay the training set or rely on protein sequence similarity, but generalizes across protein and chemical space. It is interesting to note that 221C7 contains a β-lactam ring, which may have activity beyond the scope of antibiotics. However, β-lactam ring compounds have potential side effects and risks relating to antibiotic resistance. The covalent warhead of β-lactam rings can bind irreversibly to target proteins, leading to side effects such as the generation of allergenic modified proteins^49^. In addition, the widespread use of β-lactams can increase the risk of antibiotic resistance, mainly due to the production of β-lactamase^50^. Although compounds with β-lactam rings have been reported to exhibit anticancer activity^51^ and have been used in drug development, such as cholesterol absorption inhibitors and vasopressin V1a antagonists^52^, vigilance is necessary when developing non-antibacterial agents to avoid these risks.

To further confirm that 221C7 disrupts SPOP-substrate interactions, an in vitro pull-down assay was performed. The results revealed that the compound 221C7 dose-dependently reduced the binding of PTEN protein to the SPOP MATH domain (SPOP^MATH^) (Fig. 4d). A nuclear magnetic resonance (NMR) experiment was conducted, and the result indicated direct binding between SPOP^MATH^ and 221C7 (Fig. 4e). To demonstrate that the SPOP^MATH^-PTEN interaction is not disrupted by compounds that do not to bind SPOP, we included a negative control compound, 222A5, which showed no binding to SPOP^MATH^ (Fig. 4b, Supplementary Fig. 1e). Compound 222A5 competed with peptide substrate binding to SPOP^MATH^ with an IC_50_ value > 100 μM in the FP assay (Fig. 4c), and did not disrupt the protein interaction between SPOP^MATH^ and PTEN in the in vitro pull-down assay (Supplementary Fig. 1f). These results verified that 221C7 disrupts SPOP-substrate interactions by directly binding to SPOP^MATH^.

**Fig. 5:**
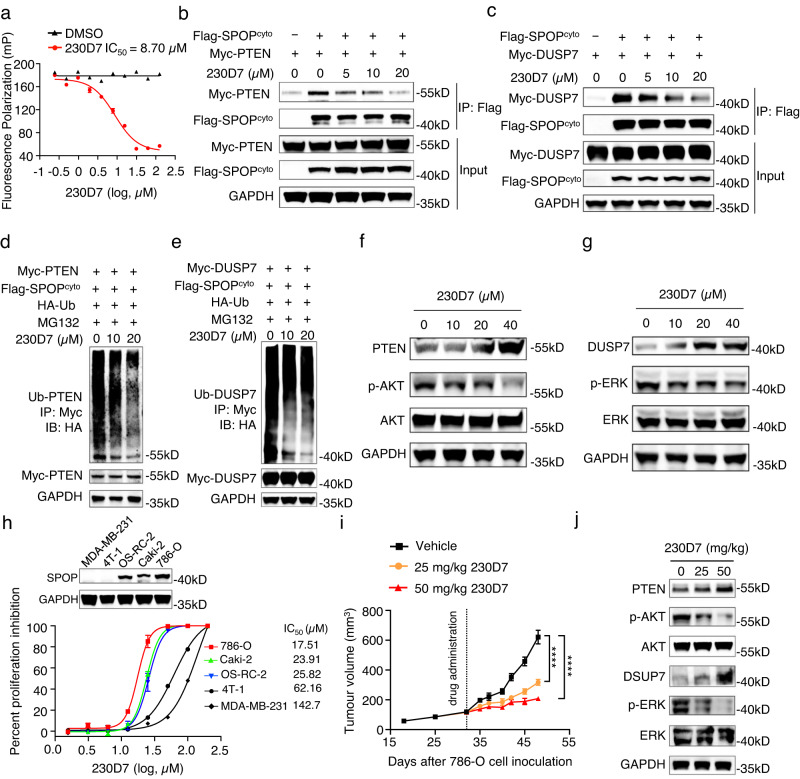
230D7 showed therapeutic potential for blocking oncogenic SPOP activity to treat ccRCC. **a** 230D7 competitively inhibits puc_SBC1 peptide binding to SPOP^MATH^, as measured by the FP assay. Error bars represent mean ± SEM of two independent experiments. **b**, **c** SPOP-PTEN and SPOP-DUSP7 protein interactions are inhibited in the presence of 230D7 in 293T cells. These experiments are repeated twice independently with similar results. **d**, **e** 230D7 inhibits the ubiquitination of PTEN and DUSP7 in 293T cells. These experiments are repeated twice independently with similar results. **f**, **g** 230D7 upregulates PTEN and DUSP7 protein levels in 786-O cells. The downstream p-AKT and p-ERK abundances are observed to decrease. These experiments are repeated twice independently with similar results. **h** Cell proliferations of three ccRCC cell lines and two non-ccRCC cell lines in the presence of 230D7. The abundance of cytoplasm SPOP protein was measured. Error bars represent mean ± SEM of three independent experiments. Western blot is repeated three times independently with similar results. **i** In vivo anti-ccRCC efficacy of 230D7 in 786-O xenograft models in NSG mice. Mice were administrated 230D7 at 25 or 50 mg/kg daily for 16 days by intraperitoneal dosing. Error bars represent mean ± SEM of seven biologically independent animals. *P* values were evaluated using 2-tailed unpaired *t*-test, ****P < 0.0001. (25 mg/kg 230D7 vs. Vehicle, *P* < 0.0001; 50 mg/kg 230D7 vs. Vehicle, *P* < 0.0001.) **j** Accumulation of PTEN and DUSP7 and repression of p-AKT and p-ERK at day 16 of 786-O xenograft tumors treated with vehicle or 230D7. This experiment is repeated twice independently with similar results. Source data are provided as a Source Data file.

The initial hit 221C7 was inactive in cell experiments, possibly due to poor cell permeability caused by its large topological polar surface area (TPSA)^53^ of 214Å^2^. Therefore, we conducted hit expansion and obtained 26 structural analogs of 221C7, 19 of which were active in the FP assay (Fig. 4f). Among them, 230D7 has a smaller TPSA (161Å^2^) and the smallest IC_50_ of the FP assay (Fig. 5a). To determine the cell permeability profile of 221C7 and 230D7, a cell permeability assay was performed. The assay showed that 221C7 displayed poor cell permeability with the extremely low intracellular content that below the detection limit, while 230D7 showed a much higher intracellular content (Supplementary Fig. 1g). This suggests that 230D7 overcame the problem of poor cell permeability. Thus, 230D7 was selected for further validation. A protein thermal shift assay (PTS) revealed dose-dependent T_m_ shifts (Supplementary Fig. 2a), indicating that 230D7 could bind directly to SPOP^MATH^. Additionally, NMR experiments confirmed the direct binding between SPOP^MATH^ and 230D7 (Supplementary Fig. 2b). An in vitro pull-down assay was performed to verify that 230D7 dose-dependently reduces PTEN binding to SPOP^MATH^ (Supplementary Fig. 2c, d). After validating the molecular activity, we used 230D7 for the functional study at the cellular level.

We firstly conducted a coimmunoprecipitation and in vivo ubiquitination experiment, and the results showed that 230D7 significantly disrupted the binding of PTEN and DUSP7 to SPOP in a dose-dependent manner (Fig. 5b, c), leading to decreases in PTEN and DUSP7 ubiquitination (Fig. 5d, e). While the negative control compound 222A5 neither disrupted the binding of PTEN and DUSP7 to SPOP, nor decreased the ubiquitination of PTEN and DUSP7 (Supplementary Fig. 2e–h). Due to the inhibition of PTEN and DUSP7 ubiquitination under 230D7 treatment, accumulation of cellular PTEN and DUSP7 proteins was observed in 786-O cells treated with 230D7, causing decreases in phosphorylated AKT and ERK (Fig. 5f, g). Next, we tested the cell proliferation of three ccRCC cell lines (786-O, Caki-2, OS-CR-2) and two non-ccRCC cell lines (4T-1, MDA-MB-231) in the presence of 230D7 (Fig. 5h). 230D7 specifically inhibited the growth of ccRCC cell lines with an IC_50_ of approximately 20 μM compared with non-ccRCC cell lines. To determine if 230D7 is suitable for in vivo studies, we investigated the pharmacokinetics and acute toxicity profile of 230D7. 230D7 can be efficiently absorbed into the blood circulation after intraperitoneal injection and has low acute toxicity (Supplementary Fig. 2i–l). A dose-dependent reduction in 786-O tumor growth rate could be observed in NSG mice treated with 230D7 (Fig. 5i), revealing a significant anti-ccRCC therapeutic effect of 230D7 in vivo. Statistically, no body weight loss was observed in NSG mice throughout the entire pharmacodynamics study of 230D7 (Supplementary Fig. 2m). Finally, we checked the effect of 230D7 on oncogenic SPOP signaling in ccRCC xenograft tumors. As expected, PTEN and DUSP7 are elevated in the 230D7 treated groups, and p-AKT and p-ERK levels are decreased (Fig. 5j). Moreover, we confirmed the high selectivity of 230D7 which does not target kinases (Supplementary Fig. 3). In conclusion, our sequence-to-drug concept has successfully identified new scaffolds targeting the protein of interest SPOP, among which 230D7 showed therapeutic potential for blocking SPOP activity to treat ccRCC.

After discovering inhibitors for SPOP, we applied this concept to discover hits for a more challenging target RNF130 whose crystal structure is unknown. RNF130 is an E3 ubiquitin-protein ligase without structural information, and no chemical binders have been reported. Therefore, the discovery of novel hits for RNF130 supports the generalization of this concept. Our recent study revealed that RNF130 plays an important role in autoimmune inflammation, suggesting that its inhibition could be of potential therapeutic value. We utilized TransformerCPI2.0 to screen compounds that bind directly to RNF130 (Supplementary Fig. 4a, Supplementary Table 10) and discovered that iRNF130-63 is a binder of RNF130 (Supplementary Fig. 4b–e). Direct binding between iRNF130-63 and RNF130 protein was confirmed through surface plasmon resonance (SPR), and this binding exhibited a fast-on, fast-off kinetic pattern with a *K*_D_ of 9.36 μM (Supplementary Fig. 4c). We also performed a cellular thermal shift assay (CETSA), and the results supported that iRNF130-63 directly binds to and thermally stabilizes the RNF130 proteins (Supplementary Fig. 4d). To further validate the binding of iRNF130-63 with RNF130 and exclude the possibility of the pan-assay interference compounds, the binding affinity was measured by isothermal titration calorimetry (ITC), widely known as a gold standard method used to determine the thermodynamic parameters of target-ligand interactions. As shown in Supplementary Fig. 4e, *K*_D_ of iRNF130-63 binding with RNF130 was 1.23 μM, ΔG and ΔH were −33.80 kJ/mol and −7.31 kJ/mol respectively, the stoichiometry of binding (N) is 1.01. Compared with other tools, iRNF130-63 was highly ranked and discovered only by TransformerCPI2.0 (Supplementary Table 11). Also, the compounds in the training set have low similarity with iRNF130-63 (Supplementary Fig. 4f), and the most similar compound targets another protein which shares very low sequence identity with RNF130 (Supplementary Fig. 4g). Success in discovering hits for SPOP and RNF130 demonstrated that the sequence-to-drug concept is practicable for virtual screening with encouraging prospects.

### Repositioning proton pump inhibitors as anticancer drugs by targeting ARF1

**Fig. 6:**
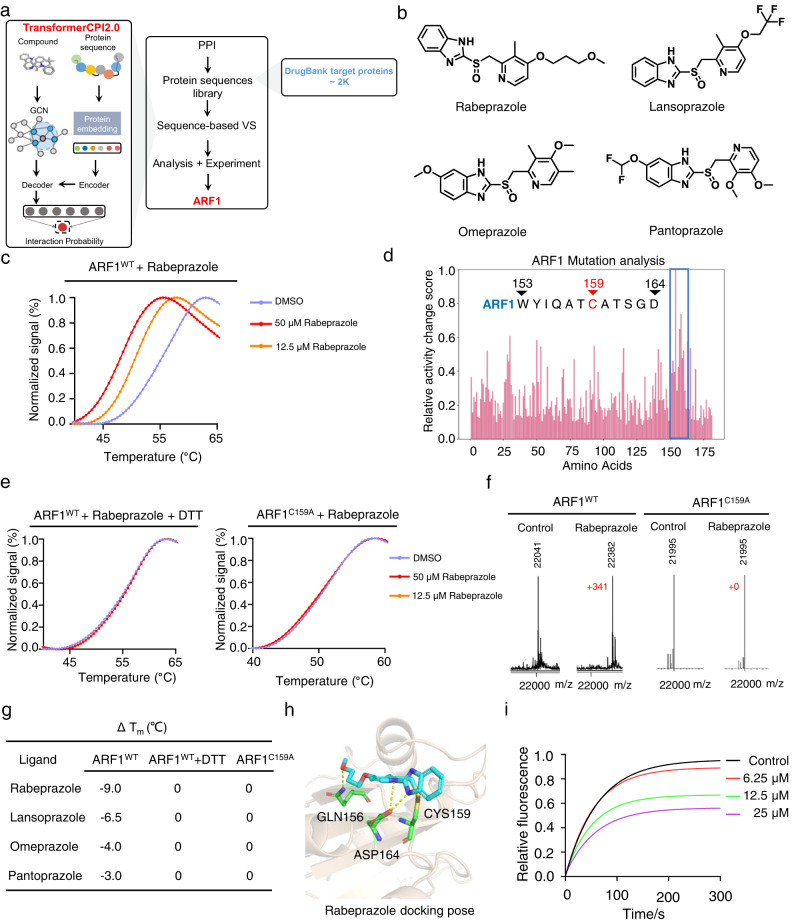
Identifying ARF1 as the new target of PPIs. **a** Scheme of target identification protocol for PPIs. **b** Chemical structures of four PPIs, rabeprazole, lansoprazole, omeprazole and pantoprazole. **c** Effect of rabeprazole (12.5 or 50 μM) on the thermal stability of ARF1^WT^ (2.5 μM) in the PTS assay. **d** Drug resistance mutation analysis interprets the prediction of omeprazole, and there is a cysteine (C159, the only cysteine residue of ARF1) residue among the important residues. **e** Effects of rabeprazole on the thermal stability of ARF1^WT^ (containing DTT) and ARF1^C159A^ in the PTS assay. **f** The protein molecular weights of ARF1^WT^ and ARF1^C159A^ in the presence or absence of rabeprazole were determined by mass spectrometer. **g** Summary of PTS assay results. **h** Potential docking pose of rabeprazole and ARF1 (PDB: 1HUR). **i** GDP/MANT-GTP nucleotide exchange of ARF1 treated with rabeprazole. Source data are provided as a Source Data file.

Benefiting from the end-to-end nature, the sequence-to-drug workflow can be inversely used to enable drug target identification or drug repurposing. This means that we can perform proteome-wide target screening, as only protein sequence information is required except for a given drug molecule as the model input. Here we selected proton pump inhibitors (PPIs) as a drug repurposing case study. To date, preclinical and clinical data support the use of PPIs in cancer treatment^54^, but few new targets have been identified. TransformerCPI2.0 was applied to score 2204 human proteins from the DrugBank database^55^ against four classic PPIs (rabeprazole, lansoprazole, omeprazole and pantoprazole, Fig. 6a, b, Supplementary Tables 12–15), and the results were sorted by predicted interaction probability. After analyzing the top 20 proteins, ARF1 attracted our attention due to its oncogenic effect on cancer stem cells (CSCs) via the lipolysis pathway^56,57^. ARF1 is a small G protein and belongs to the RAS superfamily, which switches between an active GTP-bound and an inactive GDP-bound conformation^58^. Recent studies have shown that the ARF1-regulated lipid metabolism selectively maintains cancer stem cells (CSCs) and ARF1 inhibition or knockdown in CSCs leads to accumulation of lipid droplets, further leading to metabolic stress that not only can kill CSCs selectively, but also stimulate an anticancer immune response and achieve lasting therapeutic effects^56,57^. Inhibition of ARF1 activity is a promising direction for cancer immunotherapy, therefore, we selected ARF1 for investigation.

The PTS assay revealed dose-dependent T_m_ shifts (Fig. 6c, Supplementary Fig. 5a–c), indicating that PPIs could bind directly to wild-type ARF1 (ARF1^WT^) and destabilize the protein. Drug resistance mutation analysis was then applied to interpret the prediction of TransformerCPI2.0. The results indicated that amino acids 150 to 165, a region that containing a cysteine (C159), contributed greatly to compound protein binding (Fig. 6d, Supplementary Fig. 5d). Given that PPIs covalently bind to the cysteine of H^+^/K^+^-ATPase^59^, we conducted different assays to determine whether PPIs covalently bind to ARF1. Therefore, two more PTS assays were conducted: (1) PPIs with ARF1^WT^ and the reducing agent dithiothreitol (DTT), which can break disulfide bonds; and (2) PPIs with ARF1^C159A^ where C159 was mutated to alanine. No T_m_ shifts were observed in either assay (Fig. 6e, Supplementary Fig. 5a–c). Mass spectrometry (MS) further validated that PPIs can covalently bind to ARF1^WT^ but not to ARF1^C159A^ (Fig. 6f, Supplementary Fig. 5e, f), and two-dimensional mass spectrometry determined that covalent binding site is C159 (Supplementary Fig. 6a–d). Among the four PPIs, rabeprazole had the greatest effect on the thermal stability of ARF1 (Fig. 6g), thus we selected rabeprazole for further functional studies. According to the covalent binding site, we provided a possible docking pose of rabeprazole (Fig. 6h). Activation of ARF1 requires the release of GDP followed by the binding of GTP, a process catalyzed by guanine nucleotide exchange factor (GEF)^58^. Therefore, we performed GDP/MANT-GTP nucleotide exchange catalyzed by ARNO (a type of GEF) and found that rabeprazole suppressed the nucleotide exchange process in a concentration-dependent manner (Fig. 6i), verifying its inhibition of ARF1 activity. After validating the physical binding and function of PPIs, we found that PPIs share low similarity with three known ARF1 inhibitors (Supplementary Table 16) and other baseline tools provide lower prediction scores of PPIs-ARF1 pairs (Supplementary Table 17), proving that TransformerCPI2.0 are not doing similarity search against known inhibitors.

**Fig. 7:**
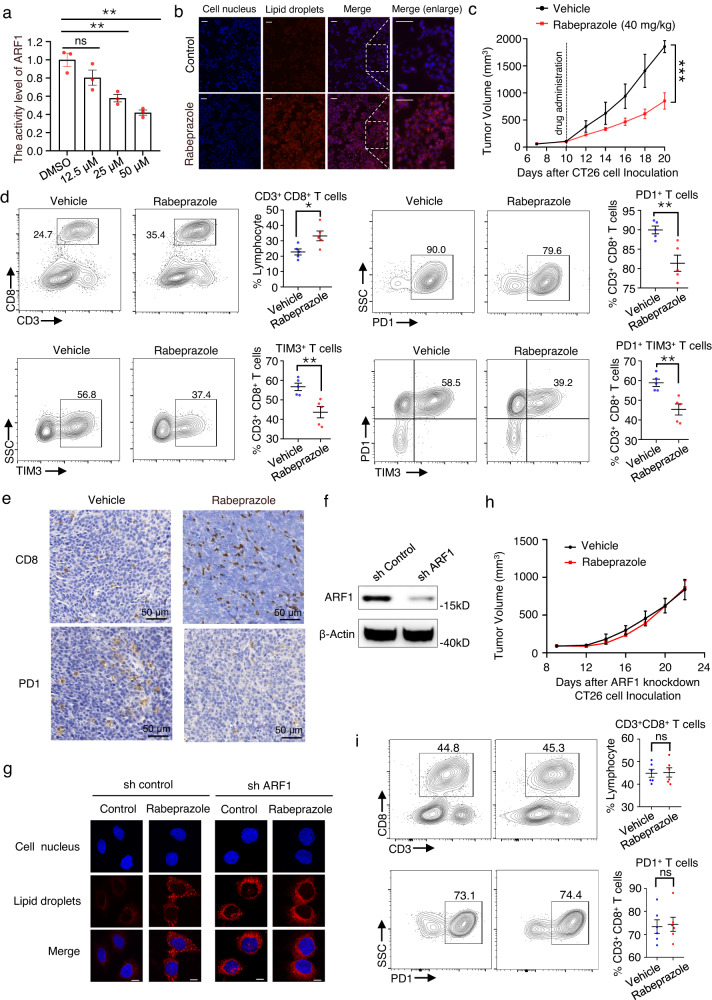
Rabeprazole induces antitumor immune response through lipid metabolism. **a** The activity level of ARF1 in CT26 cells treated with rabeprazole for 48 h was measured by using G-LISA assay. Error bars represent mean ± SEM of three independent experiments. (12.5 μM Rabeprazole vs. DMSO, *P* = 0.1497; 25 μM Rabeprazole vs. DMSO, *P* = 0.0070; 50 μM Rabeprazole vs. DMSO, *P* = 0.0017.) **b** Fluorescent images of CT26 cells stained with DAPI (for nucleus) or Nile red (for lipid droplets) after treatment with rabeprazole (20 μM) or DMSO. Scale bars: 100 μm. This experiment is repeated three times independently with similar results. **c** In vivo efficacy of rabeprazole in CT26 transplanted tumor model in BALB/c mice. Mice were administrated rabeprazole 40 mg/kg daily for 10 days by intraperitoneal dosing. Error bars represent mean ± SEM of six biologically independent animals. (40 mg/kg Rabeprazole vs. Vehicle, *P* = 0.0001.) **d** Impact of rabeprazole delivery on immune cell subsets in CT26 transplanted tumor model, assessed by flow cytometry analysis. Error bars represent mean ± SEM of five biologically independent animals. (In CD3^+^ CD8^+^ T cells: Rabeprazole vs. Vehicle, *P* = 0.0238; in PD1^+^ T cells: Rabeprazole vs. Vehicle, *P* = 0.0060; in TIM3^+^ T cells: Rabeprazole vs. Vehicle, *P* = 0.0054; in PD1^+^ TIM3^+^ T cells: Rabeprazole vs. Vehicle, *P* = 0.0037). **e** Immunohistochemical staining for cell surface markers (CD8, PD1) of tumor tissues in the indicated groups. This experiment is repeated three times independently with similar results. **f** Successful knockdown of ARF1 in CT26 cells. This experiment is repeated three times independently with similar results. **g** Fluorescent images of WT and ARF1-knockdown CT26 cells stained with DAPI (for nucleus) or Nile red (for lipid droplets) after treatment with rabeprazole (20 μM) or DMSO. Scale bars: 20 μm. This experiment is repeated three times independently with similar results. **h** In vivo efficacy of rabeprazole in ARF1-knockdown CT26 transplanted tumor model in BALB/c mice. Mice were administrated rabeprazole 40 mg/kg daily by intraperitoneal dosing. Error bars represent mean ± SEM of six biologically independent animals. **i** Impact of rabeprazole delivery on immune cell subsets in ARF1-knockdown CT26 transplanted tumor model, assessed by flow cytometry analysis. Error bars represent mean ± SEM of six biologically independent animals. *P* values were evaluated using 2-tailed unpaired *t*-test. *P < 0.05, **P < 0.01, ***P < 0.001; ns, not significant, *P* > 0.05. Source data are provided as a Source Data file.

According to previous works^56,57^, we first detected the inhibitory effect of rabeprazole on the activity level of ARF1 in CT26 cells (colon carcinoma cells) using a G-LISA assay. The results showed that rabeprazole effectively inhibited ARF1 activity in CT26 cells in a concentration-dependent manner (Fig. 7a). In addition, a significant accumulation of lipid droplets was observed in rabeprazole-treated CT26 cells (Fig. 7b). To evaluate the antitumor effect of rabeprazole in vivo, we established colon cancer transplanted tumor models by injecting CT26 cells into BALB/c mice. Rabeprazole treatment significantly suppressed the tumor growth in mice as measured by tumor volume (Fig. 7c). To verify that rabeprazole induces an antitumor immune response, we analyzed the immune cell subsets of colon cancer transplanted tumors by fluorescence-activated cell sorting (FACS) and found a significant increase in CD3^+^ CD8^+^ T cells and a significant decrease in CD3^+^ CD8^+^ PD1^+^ T cells, CD3^+^ CD8^+^ TIM3^+^ T cells and CD3^+^ CD8^+^ PD1^+^ TIM3^+^ T cells (Fig. 7d). Additionally, upregulation of CD8 and downregulation of PD1 was detected by immunohistochemical staining (Fig. 7e), confirming that an antitumor immune response was stimulated by rabeprazole. Furthermore, we investigated the effect of rabeprazole on lipid droplet accumulation and tumor growth after ARF1 knockdown to prove that the anti-tumor effect of rabeprazole is ARF1 dependent. ARF1 was successfully knocked down in CT26 cells (Fig. 7f). ARF1 depletion apparently caused lipid droplet accumulation, consistent with the reported data^56,57^, and the addition of rabeprazole had little effect on lipid droplet formation on this basis (Fig. 7g). In addition, rabeprazole failed to suppress tumor growth or affect the immune response in ARF1-knockdown CT26 transplanted tumor model (Fig. 7h, i). Taken together, these data suggested that rabeprazole induced an antitumor immune response through lipid metabolism, which is dependent on ARF1. All of this data suggests that rabeprazole inhibits the growth of colon cancer by inducing an antitumor immune response. In summary, the success of repurposing PPIs to ARF1 demonstrated that the inverse application of the sequence-to-drug concept for drug repositioning is also practicable with encouraging prospects.

## Discussion

The conventional structure-based drug design pipeline is a complex, human-engineered process with multiple independently optimized steps. However, the multistep operation is error-prone due to factors such as inaccurate protein structures, the multiplicity and dynamics of binding pockets, incorrect pocket definition, inappropriate selection of the scoring function, etc. The errors or intrinsic accuracy limitations of each step accumulate rapidly and significantly lower the success rate. When little information about target proteins is available, this issue becomes more serious and constitutes a long-lasting obstacle to rational drug design.

To address this issue, we proposed a sequence-to-drug concept and developed TransformerCPI2.0 to validate this concept on three targets. These targets are challenging and only a few active molecules have been reported. Apart from methodology, the sequence-to-drug concept successfully discovered a inhibitor for SPOP, for which only one active scaffold was reported before, and discovered the first binder of RNF130. Given that SPOP is an adapter of E3 ligase and RNF130 is an E3 ligase, the hits we found have the potential to serve as novel warheads for proteolysis-targeting chimaeras (PROTACs). PROTACs have been successfully developed for harnessing the ubiquitin–proteasome system to degrade a protein of interest, receiving tremendous attention as a new and exciting class of therapeutic agents that promise to significantly impact drug discovery. Furthermore, through an inverse application of the sequence-to-drug concept, the FDA-approved drug rabeprazole showed promise for expanding its indications to colon cancer treatment by regulating lipid metabolism and inducing an antitumour immune response. Additionally, these targets and their corresponding active molecules are not seen in the training phase, and the hits we reported share low similarity with the known active molecules and fail to be discovered by other tools, which supports that our concept is not replaying the training set but can generalize across protein and chemical space. Overall, our findings provide a proof of sequence-to-drug concept, which we believe will become an essential component of future rational drug design pipelines.

However, our work does not guarantee the success to any novel targets. We appreciate and respect other drug discovery tools, and our aim is to add a new perspective to drug design. The rigorous conclusion we draw is that our work can serve as an alternative method to SBDD, and it can be used in combination with other in silico or in vivo tools, to help the community accelerate the drug discovery progress.

Recently, due to the rapid growth of virtual chemical libraries, such as GDBChEMBL^60^, SCUBIDOO^61^, ZINClick^62^, GDB-17^63^, FDB-17^64^, DrugSpaceX^65^ and synthesis (REAL) combinatorial libraries^66^, which cover spaces of 100 million to multiple billions of chemicals, there is a high demand for developing computationally efficient virtual screening approaches. The sequence-to-drug concept and TransformerCPI2.0 can be combined with these large virtual libraries to rapidly discover active scaffolds from the unexplored chemical space.

## Methods

### TransformerCPI2.0 model and training details

Compared with TransformerCPI, TransformerCPI2.0 has been updated in the following four aspects: (1) removing 3-gram protein word embedding calculated by the Word2Vec algorithm, (2) computing protein sequence representation by a pretrained protein language model named TAPE-BERT, (3) replacing 1D convolutional neural networks and gated linear units with a self-attention-based transformer encoder, and (4) introducing a new atom vector into the atom sequence that carries the interaction information at the molecular level.

### Pretraining the protein language model

Word2vec is an unsupervised technique to learn high-quality distributed vector representations that describe sophisticated syntactic and semantic word relationships and maps discrete words to low-dimensional real-valued vectors. However, the final embedding table of Word2vec is stationary, regardless of the upstream and downstream context information of the given word, which may lead to errors regarding the true meaning of the word in its local context. Since BERT^67^ achieved great success in natural language processing (NLP), many efforts have been devoted to protein sequence representation learning. Many pretraining models based on long short-term memory (LSTM) or transformer architectures have been proposed, such as UniRep^68^ and TAPE^69^. To maintain the model consistency and gain parallel computing efficiency, we chose the transformer model in TAPE (TAPE-BERT) to calculate protein sequence embedding. The TAPE-BERT model contains 12 self-attention encoder layers, 12 attention heads for each layer, 768 dimensions for the hidden state, and 3072 dimensions for feedforward layers. We first utilized the TAPE Tokenizer from TAPE-BERT to encode the protein amino acid sequence into real values, where numbers 1–23 represent 23 common amino acids, 0 represents the token ‘<pad>’, 24 represents the token ‘<cls>’, and 25 represents the token ‘<sep>’. Then, we input this encoded real value sequence into the pretrained TAPE-BERT model and finally obtained protein embeddings with 768 dimensions. In TransformerCPI2.0, protein embedding from the TAPE-BERT model serves as the input to the encoder of TransformerCPI2.0, replacing the embedding calculated from the Word2Vec model.

### Encoder of TransformerCPI2.0

Since protein embedding was calculated by the TAPE-BERT model, we replaced 1D convolutional neural networks and gated linear units with the original self-attention-based transformer encoder. Given that the position information of the amino acid sequence has been taken into consideration when computing protein embeddings, position embedding was removed from the transformer encoder. The encoder consists of 3 encoder layers, 8 attention heads for each layer, 768 dimensions for the hidden state, and 3072 dimensions for feedforward layers. To maintain the maximal performance of TAPE-BERT, the hidden state dimension of the encoder was exactly the same as that of TAPE-BERT, ensuring no information loss in this process. After the hyperparameter search, 12 attention heads showed little performance improvement compared to 8 attention heads but much higher training and inference time; therefore, only 8 attention heads were used in TransformerCPI2.0.

### Atom embedding calculation

Each of the atom features was initially represented as a vector of size 34 using the RDKit python package, and the list of atom features can be found in our previous work. In TransformerCPI2.0, we additionally introduced a new virtual atom that carries the information at the molecular level and does not exist in the given compound. This virtual atom was initialized as the average of atom features across the whole compound and was linked to all atoms. All the atom vectors together with the virtual atom vector were put into GCNs^70^ to learn the representation by integrating their own neighborhood information. Notably, only one GCN layer was used and recommended in this process, and more than two GCN layers harmed the performance of TransformerCPI2.0 to a great deal. Too many GCN layers may over smooth the atom features, causing different atom features to tend to be similar to each other. TransformerCPI2.0 then fails to learn compound-protein interaction features when the atom embedding carries excessively similar features. A table of atomic embedding features is shown in the Supplementary Table 18.

### Decoder of TransformerCPI2.0

Protein embedding and atom embedding serve as the target sequence and memory sequence of the transformer decoder, respectively. Consistent with the encoder, the decoder consists of 3 decoder layers, 8 attention heads for each layer, 768 dimensions for the hidden state, and 3072 dimensions for feedforward layers. In addition, the original transformer was designed to solve seq2seq tasks and utilize a causal mask operation to cover the downstream context of each word in the decoder. We removed the mask operation of the decoder to ensure that our model accesses the whole target sequence. Since we introduced a new virtual atom, as described above, we used the last layer representation of this virtual atom rather than the weighted sum of the last layer atom representation to predict the compound protein interaction probability. The last layer presentation of virtual atoms was fed into fully connected layers and finally returned the compound protein interaction probability.

### Training details

The TransformerCPI2.0 model was trained by the RAdam^71^ optimizer with a learning rate of 1e-5 and a weight decay of 1e-3. The batch size of 1 was selected to ensure that the longest protein sequence fit into the GPU memory. We employed the gradient accumulation technique to expand the actual batch size to 64. Training was performed on one NVIDIA Tesla V100 (16G) GPU. The TransformerCPI2.0 model was trained for ~50 epochs or ~1.5 weeks of wall clock time.

### ChEMBL dataset construction

Inheriting our previous work, we followed two rules to construct a dataset: (i) CPI data was collected from an experimentally validated database and (ii) each ligand should exist in both positive and negative classes. To build a universal deep learning model for all types of proteins, we selected the ChEMBL_23^72^ database to construct a universal dataset to train TransformerCPI2.0.

### Data cleaning

The ChEMBL_23 database was released on 1 May 2017 and contains 2,101,843 compound records, 1,735,442 compounds, 14,675,320 activities, 1,302,147 assays, 11,538 targets and 67,722 source documents. We downloaded the whole database and cleaned the data using the following procedure:

1. The target type was set to ‘SINGLE PROTEIN’, and the molecule type was set to ‘Small molecule’;
2. Data with a confidence score of 9 and assay type of ‘B’ were reserved;
3. Activity data with activity metrics of IC_50_, EC_50_, K_i_ in units of nM were selected.

### Dataset process

After cleaning the data, we transformed IC_50_, EC_50_, and K_i_ to pIC_50_, pEC_50_, and pK_i_ and then split the dataset into a positive set and a negative set at the threshold of 6.5. Samples with different labels were removed from the dataset. In the early stage of drug discovery, only hit compounds whose IC_50_, K_i_ or EC_50_ are at the μM or even nM level will be further optimized. Additionally, public data are prone to a certain experimental error, i.e., on average 0.5 log units for IC50 data^73,74^. The threshold of 6 considers those CPI pairs whose IC50, Ki or EC50 are smaller than 1 μM as positive samples, which causes the models to select CPI pairs with high activity. To decrease the risk of experimental error in public data, a stricter threshold of 6.5 was used in a previous work^75^. Therefore, we selected the threshold of 6.5 to define positive data and negative data. Data whose atom number was more than 60 or whose protein sequence length exceeded 4000 were filtered out, guaranteeing that all the data fit into GPU memory. Finally, we constructed a ChEMBL dataset including 3348 proteins, 69,616 compounds, 117,513 positive CPIs, 134,611 negative CPIs and 252,124 samples in total.

### Dataset split and label reversal experiment

We selected ligands that exist in both the positive and negative classes to make the compound distribution in positive samples and negative samples exactly the same, trying to eliminate the potential ligand bias as much as possible. Consequently, we used a label reversal experiment to split the ChEMBL dataset. The mechanism of label reversal experiment is that some ligands in the training set appear only in one class of samples (either positive or negative interaction CPI pairs), while have the opposite labels with other proteins in the test set. In this way, the model was forced to utilize protein information to understand interaction modes and make opposite predictions for those chosen ligands. If a model only memorizes the ligand patterns, it is unlikely to make correct predictions because the ligands it memorizes have the wrong (opposite) labels in test set. Therefore, this label reversal experiment is specifically designed to evaluate CPI models and is capable of indicating how much influence the hidden variables have exerted. For the ChEMBL dataset, we randomly selected 2941 ligands and pooled all the negative CPI samples containing these ligands into the test set. Additionally, we selected another 2900 ligands and pooled all their associated positive samples into the test set. The remaining datasets were split randomly into a training set and a validation set at a ratio of 10:1. The validation set was used to determine the hyperparameters, and the best model was evaluated on the test set. Under this experimental design, we finally established a ChEMBL set containing 217,732 samples in the training set, 24,193 samples in the validation set and 10,199 in the test set.

### External evaluation on external datasets

All the baseline models and TransformerCPI2.0 were tested on the external test set and time-split ChEMBL27 set. This external set contains compounds that were not previously observed and 1192 new protein targets that were not included in the training set. The total number of CPI pairs is 342,447, and the ratio of positive samples to negative samples is 1:3. This external set can evaluate the generalization ability of TransformerCPI2.0 and baseline models to the new compounds and new proteins. Another time-split dataset named the ChEMBL27 dataset contains compounds that were not previously observed and 637 new protein targets that are not included in the training set, and all the data were collected from the ChEMBL_27 database. The total number of CPI pairs is 92,919, and the ratio of positive samples to negative samples is 1:1.

### Baseline models

All the baseline models, including TransformerCPI, CPI-GNN, GraphDTA(GAT-GCN), MolTrans and GCN, were trained on the ChEMBL dataset with their own hyperparameters. Only the learning rate, weight decay rate and dropout rate were subjected to a hyperparameter search.

### Drug resistance mutation analysis

Activity change score calculation. First, we input the wild-type protein and drug into TransformerCPI2.0 to calculate the original prediction score, denoted as $s$. Then, we mutated each amino acid of the protein sequence to all 20 amino acids (including itself) and calculated the prediction score ${s{{\hbox{'}}}}$. Finally, we defined the activity change score ${{\Delta }}{{{{{\bf{S}}}}}}\in {{\mathbb{R}}}^{l\times 20}$ as

1 $$\Delta {S}_{i,j}={{{{{\rm{|}}}}}}\,s-{s}_{i,j}^{{\prime} }\,{{{{{\rm{|}}}}}},\,i=1,2,\ldots,l,\,j=1,2,\ldots,20.$$

Here, $i$ corresponds to the position of the protein sequence, $l$ corresponds to the length of the protein sequence, and $j$ corresponds to 20 types of amino acids. Since an amino acid mutation will increase or decrease the prediction score ${s{{\hbox{'}}}}$ and TransformerCPI2.0 may not be able to predict the trend of activity changes correctly, we calculated the absolute value of Δ**S** here. We analyzed Δ**S** and found that TransformerCPI2.0 actually learns the key features of compound protein interactions because the pattern of Δ**S** revealed by heatmap analysis is consistent with that of drug resistance mutation.

### Relative activity change score

After calculating the activity change score, we can evaluate whether a mutation at a specific position plays an important role in compound protein interactions. However, ${{\Delta }}{{{{{\bf{S}}}}}}$ cannot quantify the contribution of each position on a protein sequence and rank the most important sites from the whole sequence. To quantify the contribution of each amino acid site to drug-protein interactions, we first computed the average score of each position Δ$\bar{{{{{{\bf{S}}}}}}}\in {{\mathbb{R}}}^{l}$ as

2 $$\Delta {\bar{S}}_{i}=\frac{\mathop{\sum }\nolimits_{j=1}^{20}{s}_{i,j}^{{\prime} }}{20}.$$

Furthermore, the value of $\Delta\bar{{{{{{\bf{S}}}}}}}$ was normalized to between 0–1, and the relative activity change score $\Delta{{{{{\bf{R}}}}}}\in {{\mathbb{R}}}^{l}$ was defined as

3 $$\Delta {R}_{i}=\frac{\Delta {\bar{S}}_{i}}{\max (\Delta {\bar{S}}_{i})},\, i=1,2,\ldots,l.$$

The relative activity change score Δ**R** can then characterize the contribution of each amino acid position to TransformerCPI2.0 prediction and help researchers discover novel and potential drug resistance mutation sites. On the other hand, Δ**R** can reflect the compound–protein interactions in TransformerCPI2.0. An amino acid site whose contribution to compound–protein binding is large can be revealed quantitatively by the relative activity change score Δ**R**. Finally, we used the activity change score Δ**S** to plot a heatmap to study the concrete pattern of drug resistance mutations and the relative activity change score Δ**R** to rank the most important sites for compound protein binding.

### Analysis of the substitution effect of the trifluoromethyl group

The replacement of methyl (Me or −CH_3_) with trifluoromethyl (TFM or −CF_3_) is frequently employed in compound optimization. However, the exact effect of −CH_3_/–CF_3_ substitution on bioactivity is still controversial. To further investigate whether TransformerCPI2.0 captures the key features of the compound and comprehensively understands compound–protein interaction, we employed TransformerCPI2.0 to predict the substitution effect of the trifluoromethyl group. We utilized a previously reported dataset, removed the redundancy data and finally got a dataset containing 18,217 pairs of compounds and corresponding bioactivity data with the only difference being that −CH_3_ is substituted by −CF_3_ to study this problem. We checked this dataset with our training set and found only 1,062 pairs (5.8%) were overlapped. The majority of this dataset are not seen by TransformerCPI2.0 and baseline models during the training phase, so it can measure the generalization of these models to some extent. However, this analysis does not prove that TranformerCPI2.0 can solve activity cliff prediction problems. We stress that this analysis is aim to show that TransformerCPI2.0 can capture activity-related information of compounds, and can served as interpretation tools. The interpretation results should be taken with caution.

### Dataset analysis and data cleaning

The statistical results showed that the replacement of −CH_3_ with −CF_3_ does not improve bioactivity on average. However, in 15.73% of cases, substituting −CF_3_ for −CH_3_ increased or decreased the biological activity by at least an order of magnitude, and we called this part of data as the whole dataset. Only 4.6% data in the whole dataset are overlapped with the training set. Besides, we designed a subset consists of 188 data point where substitution of −CH_3_ by −CF_3_ could increase or decrease the biological activity by at least three orders of magnitude. Only 3 data points are overlapped with the training set. The overlapped data points were removed from our analysis. The actual bioactivity change $\Delta {pAct}$ was defined as

4 $$\Delta {pAct}={pAct}\left(-{{{{{\rm{CF}}}}}}3\right)-{pAct}\left(-{{{{{\rm{CH}}}}}}3\right){{{{{\boldsymbol{.}}}}}}$$

### Activity change score

First, we used TransformerCPI2.0 to calculate the prediction scores of compounds with trifluoromethyl substituents and denoted this score as ${{score}}_{-{CF}3}$. Then, we calculated the prediction scores of compounds with methyl substituents and denoted this score as ${{score}}_{-{CH}3}$. The activity change score $\Delta {s}_{c}$ was defined as

5 $$\Delta {s}_{c}={{score}}_{-{CF}3}-{{score}}_{-{CH}3}{{{{{\boldsymbol{.}}}}}}$$

Considering that the distributions of $\Delta {pAct}$ and $\Delta {s}_{c}$ were different from each other, we defined a correct prediction by a model as $\Delta {pAct}$ and $\Delta {s}_{c}$ sharing the same sign. In other words, when the activity change trend predicted by the model matches the actual bioactivity change, the prediction is considered correct. After defining the evaluation metrics, we analyzed the performance of TransformerCPI2.0 on the whole dataset. Furthermore, we investigated the prediction performance on the cases where the corresponding biological activities increased or decreased by at least three orders of magnitude because these cases are relevant to the activity cliff phenomenon in medicinal chemistry. Finally, we selected four cases that were not observed in the training set to show the power of TransformerCPI2.0.

### Virtual screening of SPOP

First, after filtering non-drug-like compounds from the ChemDiv Library (San Diego, CA, USA), which contains approximately 1.6 million in-stock compounds, TransformerCPI2.0 was applied to score the compounds, and the top 35,000 molecules (~top 2%, ensuring compound diversity) were selected by screening. Second, we filtered pan assay interference compounds (PAINS) and clustered these molecules automatically based on their extended-connectivity fingerprints (ECFP), obtaining approximately 800 clusters. Third, we filtered these compounds by the Lipinski rules and selected representative compounds from top ranked clusters. Finally, a total of 82 candidates were purchased for further experimental evaluation.

### Virtual screening of RNF130

First, after filtering non-drug-like compounds from the Chemspace Library (Monmouth Junction, NJ 08852, USA), which contains approximately 2 million in-stock compounds, TransformerCPI2.0 was applied to score the compounds, and the top 10,000 molecules (~top 0.5%, ensuring compound diversity) were selected. Second, we filtered pan assay interference compounds (PAINS) and clustered these molecules automatically based on their extended-connectivity fingerprints (ECFP), obtaining approximately 200 clusters. Third, we filtered these compounds by the Lipinski rules and selected representative compounds from top ranked clusters. Finally, a total of 87 candidates were purchased for further experimental evaluation.

### Target identification of PPIs

We collected potential proteins from the DrugBank database and selected proteins that already have active modulators. Then, TransformerCPI2.0 was applied to score proteins against four classical PPIs (omeprazole, rabeprazole, lansoprazole and pantoprazole), and the results were sorted by predicted interaction probability. Next, we analyzed the top 20 proteins by evaluating their novelty, importance and feasibility, and finally chose ARF1 for experimental validation.

### Compounds

SPOP inhibitors were purchased from ChemDiv Library (San Diego, CA, USA): 221C7, Y502-3210; 231A10, 5282-0816; 231D8, 8017-3040. 230D7 was synthesized in our laboratory. RNF130 inhibitor was purchased from Chemspace Library (Monmouth Junction, NJ 08852, USA): iRNF130-63, CSC138461036. PPIs were purchased from MedChemExpress (Monmouth Junction, NJ, USA): rabeprazole, HY-B0656; lansoprazole, HY-13662; Omeprazole, HY-B0113; pantoprazole, HY-17507.

### Plasmid construction

Wild-type, truncated or mutant versions of the human proteins were used in this study: SPOP (UniProt accession code: O43791-1), PTEN (P60484-1), DUSP7 (Q16829-1), RNF130 (Q86XS8-1), ARF1 (P84077-1) and ARNO (Q99418-1). For plasmid construction, SPOP^WT^ and SPOP^cyto^ (residues 1-366) were subcloned into the pcDNA 3.1 vector with a Flag-tag, SPOP^MATH^ (residues 28–166) and ARNO^Sec7^ (residues 50–250) was subcloned into the pGEX 6p-1 vector with a GST-tag, PTEN and DUSP7 were subcloned into the pcDNA 3.1 vector with a Myc-tag, RNF130 (residues 1–304) was subcloned into the pcDNA3.1 vector with a C-terminal His_8_-Flag-tag. N-terminally truncated human Δ17ARF1 and C159A-mutant Δ17ARF1 were subcloned into pProEX HTb with a His_6_-tag. All of the above plasmids were synthesized by Sangon Biotech (Shanghai) Co., Ltd. pCMV-HA-Ub plasmid (CAT#. kl-zl-0513) was purchased from Shanghai Kelei Biological Technology Co., Ltd.

### Recombinant protein expression and purification

For expression of SPOP^MATH^, GST-tagged SPOP^MATH^ plasmid was transformed into BL21-CodonPlus (DE3)-RIPL Cells (CAT#. EC1007, Shanghai Weidi Biotechnology Co., Ltd), and then the cells were grown in lysogeny broth (LB) medium and induced by isopropyl β-D-1-thiogalactopyranoside (IPTG) at a final concentration of 0.5 mM at 16 °C overnight. Cells were harvested and lysed in solution (20 mM HEPES pH 7.4, 200 mM NaCl, 1 mM dithiothreitol) by sonication and then centrifuged at 32,914 × *g* for 1 h at 4 °C. The supernatants were filtered by 0.22 μM syringe filters and purified on GST Trap columns (GE Healthcare) by elution with 10 mM reduced glutathione. The eluted components were loaded onto desalting columns (GE Healthcare) to remove reduced glutathione and incubated with PreScission Protease for 6–8 h at 4 °C. The components were reloaded onto GST Trap columns to remove the GST tags and further purified by a Superdex 75 10/300 GL column. The purified SPOP^MATH^ protein was concentrated and stored in buffer (20 mM HEPES pH 7.4, 200 mM NaCl) at −80 °C.

RNF130 protein was expressed in Expi-293F (Invitrogen) using Expifectamine transfection reagent according to the manufacturer’s instructions. Cells were collected 3 days after transfection. Proteins were first captured by Ni^2+^-Sepharose 6 Fast Flow resin (GE healthcare) and then further purified by gel filtration chromatography with a Superdex S200 column (GE Healthcare). The purified protein was concentrated and stored in buffer (20 mM HEPES pH 7.5, 150 mM NaCl and 1 mM TCEP) at −80 °C.

For expression of Δ17ARF1 and Δ17ARF1^C159A^, the His-tagged recombinant plasmid was transformed into BL21-CodonPlus (DE3)-RIPL cells, and then the cells were grown in LB medium and induced by IPTG at a final concentration of 0.1 mM at 25 °C for 6 h. The cells were harvested and lysed in solution (20 mM Tris, 100 mM NaCl, 5 mM MgCl_2_, 10 mM imidazole, pH 8.0) by sonication and then centrifuged at 32,914 × *g* for 1 h at 4 °C. The supernatants were filtered by 0.22 μM syringe filters and purified on HiTrap column (GE Healthcare) by elution with 300 mM imidazole. Protein sample was then purified by a Superdex 75 10/300 GL column. Finally, the purified Δ17ARF1 and Δ17ARF1^C159A^ proteins were concentrated and stored in buffer (20 mM Tris, 100 mM NaCl, pH 8.0) at −80 °C.

The purification process ARNO^Sec7^ protein was the same as that of the SPOP^MATH^ protein except the cells were induced by 0.1 mM IPTG at 37 °C for 3 h.

### Fluorescence polarization (FP)

Fluorescence polarization experiments were conducted in a 384-well black plate (Corning, 3575) using a 42 μL reaction system. FITC-labeled SPOP substrate puc_SBC1 (FITC-LACDEVTSTTSSSTA) (synthesized by GL Biochem (Shanghai) Ltd) was used for the probe. Then, 20 μL of reaction buffer (20 mM HEPES, pH 7.4) containing 200 nM SPOP^MATH^ protein was incubated with 2 μL of compound for 30 min at room temperature, and 20 μL of reaction buffer containing 200 nM probe was added. Fluorescence polarization (mP) signals were measured by a fluorescence mode (excitation filter 480 nm, emission filter 535 nm) in Spark microplate reader (Tecan).

### Protein thermal shift (PTS)

The Bio–Rad CFX96 RealTime PCR Detection System was utilized to monitor the thermal stability of ARF1 and SPOP^MATH^ protein. PTS experiments were performed in a 96-well PCR plate (DN Biotech (Hong Kong) Co., Ltd.) with a 20 μL reaction system. A total of 20 μL of reaction buffer containing protein (5 μM for SPOP^MATH^ or 2.5 μM for ARF1), 5 × SYPRO Orange Protein Gel Stain (Sigma, S5692) and indicated concentration of compound. The signals of all reaction systems were continuously monitored and recorded from 25 °C to 90 °C for approximately 45 min. The T_m_ values of SPOP^MATH^ and ARF1 were measured using CFX manager software version 3.1.

### Nuclear magnetic resonance (NMR)

NMR spectroscopy experiments were performed using a 600 MHz spectrometer (AVANCE III, Bruker) to validate protein–ligand interactions. In Carr-Purcell-Meiboom-Gill (CPMG) and saturation transfer difference (STD) NMR experiments, compound was dissolved to a final concentration of 200 μM in a solution of PBS formed with D_2_O containing 5 μM SPOP^MATH^ protein and 5% DMSO‑*d*_6_.

### Mass spectrometry analysis

The experiment was performed on the mass spectrometry service platform of Shanghai Institute of Materia Medica, Chinese Academy of Sciences. The protein (100 μM) was incubated with compounds (1 mM) or solvent control overnight at 4 °C, and then the protein molecular weights were determined by Q Exactive (Thermo) and 6545 XT (Aglient) mass spectrometer. For compound binding site identification, the proteins were digested with trypsin (10 ng/μL) at 37 °C for 17 h. The next day, after centrifugation, the supernatant was lyophilized, desalted, and lyophilized again, followed by the addition of 0.1% FA solution to dissolve peptide lyophilized powder. After centrifugation, the supernatant was detected by mass spectrometry (Q-Exactive). The MS data was analyzed via software MaxQuant (version 1.6.5.0). The false discovery rate (FDR) for peptides and proteins was controlled <1% by Andromeda search engine.

### Surface plasmon resonance (SPR)

The SPR binding assay was performed using a Biacore T200 instrument (GE Healthcare). The purified RNF130 protein was covalently immobilized onto a CM5 sensor chip (Cytiva) by a standard amine-coupling procedure in 10 mM sodium acetate (pH 4.5) with running buffer HBS (50 mM HEPES pH 7.4, 150 mM NaCl). iRNF130-63 was serially diluted and injected onto the sensor chip at a flow rate of 30 μL/min for 120 s (contact phase), followed by 120 s of buffer flow (dissociation phase). The equilibrium dissociation constant (*K*_*D*_) value was derived using Biacore T200 Evaluation software (version 1.0, GE Healthcare).

### Isothermal titration calorimetry (ITC)

The binding parameters of the compound iRNF130-63 to RNF130 were measured with a MicroCal PEAQ-ITC calorimeter. The RNF130 protein was diluted to 25 µM. Then, 2 µL of iRNF130-63 (300 μM) was added to the RNF130 protein. The data were analyzed using MicroCal PEAQ-ITC software.

### Guanine nucleotide exchange assay

First, with the participation of EDTA (a metal chelating agent capable of chelating magnesium ions, which are critical for the binding of GDP/GTP to ARF1), GDP was loaded onto the ARF1 protein by incubating ARF1 with a 20-fold molar concentration of GDP. Excess magnesium chloride was used to terminate the loading reaction, followed by the removal of excess GDP by a NAP-5 column to produce ARF1^GDP^ protein. Next, ARF1^GDP^ protein (20 μM) was mixed with compounds and Mant-GTP (10 μM) in reaction buffer and incubated in the dark for 15 min. Exchange reactions were initiated by the injection of ARNO^Sec7^ (1 μM).

### Cell lines

293T (CRL-3216), MDA-MB-231 (HTB-26), 4T-1 (CRL-2539) and CT26 (CRL-2638) cells were obtained from the American Type Culture Collection (ATCC). OS-RC-2 (1101HUM-PUMC000292) and Caki-2 (1101HUM-PUMC000337) cells were purchased from the National Biomedical Laboratory Cell Resource Bank. 786-O (TCHu186) cells were kindly provided by the Cell Bank/Stem Cell Bank, Chinese Academy of Sciences. 293T and MDA-MB-231 cells were cultured in DMEM medium (BasalMedia, L110KJ) supplemented with 10% fetal bovine serum (FBS, Gibco, 10099141C) and 1% Penicillin-Streptomycin (PS, Gibco, 2321118). 786-O, OS-RC-2, 4T-1 and CT26 cells were cultured in RPMI-1640 medium (BasalMedia, L210KJ) supplemented with 10% FBS and 1% PS. Caki-2 cells were cultured in McCoy’s 5A medium (Gibco, 2193071) supplemented with 10% FBS and 1% PS. All cells were incubated at 37 °C under a 5% (v/v) CO_2_ atmosphere.

### G-LISA

ARF1 activity was measured using corresponding G-LISA Activation Assay Kits (Cytoskeleton, Denver, CO, USA). Briefly, CT-26 cells were treated with different concentrations of rabeprazole and lysed using the provided cell lysis buffer, then lysates were collected by centrifugation at 16,260 × *g* at 4 °C for 1 min. Protein concentrations from each sample were quantified and adjusted to identical concentration for the assay. ARF1 activity was assessed according to the manufacturer’s instructions.

### Western blot

Total proteins from cells were lysed in RIPA lysis buffer (Beyotime, P0013C) containing phosphatase inhibitor (Bimake, B15001) and protease inhibitor (Bimake, B14001) on ice. Cell lysates were centrifuged at 13,000 × *g* for 15 min at 4 °C. The BCA protein assay kit (Thermo Scientific, 23225) was used to quantify the protein concentration. Equal amounts of total proteins were separated by 10% SDS–PAGE and then transferred onto nitrocellulose membranes. The membranes were blocked with 5% skim milk in TBST for 1 h at room temperature and then incubated with primary antibodies overnight at 4 °C. Then membranes were incubated with HRP-conjugated anti-rabbit antibody (secondary antibody, Promega, W4011, 1:1000) for 1 h at room temperature. Finally, the immune complexes were detected with an ECL kit (Meilun, MA0186) and visualized as well as quantified using GenGnome XRQ NPC. The following primary antibodies were used: anti-Flag (Cell Signaling Technology, 14793, 1:1000), anti-myc (Cell Signaling Technology, 2278, 1:1000), anti-GAPDH (Cell Signaling Technology, 5174, 1:1000), anti-PTEN (Cell Signaling Technology, 9559, 1:1000), anti-HA (Cell Signaling Technology, 3724, 1:1000), anti-p-ERK^T202/Y204^ (Cell Signaling Technology, 4376, 1:1000), anti-p-AKT^Thr308^ (Cell Signaling Technology, 4056, 1:1000), anti-ERK (Cell Signaling Technology, 9102, 1:1000), anti-AKT (Cell Signaling Technology, 9272, 1:1000), anti-DUSP7 (ABGENT, AP8450a, 1:1000), anti-SPOP (Abcam, ab192233, 1:1000), anti-GST (Absin, abs830010, 1:1000) and anti-β-Tubulin (Cell Signaling Technology, 15115, 1:1000).

### In vitro GST pull-down

The plasmids (Myc-PTEN or Myc-DUSP7) were transiently transfected into 293T cells. After transfection for 24 h, the cells were harvested and lysed in cell lysis buffer for Western and IP (Beyotime, P0013) containing protease inhibitor on ice. GST or GST-SPOP^MATH^ proteins bound to GST magnetic beads (GenScript, L00327) were incubated with the cell lysates (Myc-PTEN or Myc-DUSP7) in the presence of different doses of compound for 2 h at room temperature. The beads were washed 3 times with PBST, and the precipitated proteins were eluted with 1 × SDS loading buffer (Beyotime, L00327) at 100 °C for 5 min and analyzed by Western Blot.

### Cellular thermal shift assay (CETSA)

293T cells transfected with Flag-RNF130 for 48 h were collected and lysed in 20 mM Tris pH 7.5, 150 mM NaCl and 1% Triton X-100. Then, 50 μM iRNF130-63 or DMSO was added to the supernatant and incubated at 25 °C for 30 min. After denaturing at various temperatures for 3 min on a temperature gradient PCR instrument (Eppendorf), the samples were centrifuged at 20,000 × *g* for 30 min at 4 °C, and the supernatants were analyzed by western blot.

### Coimmunoprecipitation (Co-IP)

The plasmids (Flag-SPOP^cyto^, Myc-PTEN or Myc-DUSP7) were transiently cotransfected into 293T cells. After transfection for 24 h, 293T cells were treated with different doses of compound for another 24 h. The cells were harvested and lysed in cell lysis buffer for Western and IP containing protease inhibitor on ice. Approximately 80% of the total lysates were immunoprecipitated with anti-Flag-conjugated magnetic beads (Bimake, B26102) for 2 h at room temperature, and other lysates were used as input. The magnetic beads were then washed 3 times with PBST, and the immunoprecipitated proteins were eluted with 1 × SDS loading buffer at 100 °C for 5 min. The IP and lysate samples were analyzed by western blot.

### In vivo ubiquitination

The plasmids (Myc-PTEN or Myc-DUSP7, Flag-SPOP^cyto^, HA-Ub) were transiently cotransfected into 293T cells. After transfection for 24 h, 293T cells were treated with different doses of compound for 24 h. The cells were then treated with 10 μM protease inhibitor MG132 (MedChemExpress (Monmouth Junction, NJ, USA), HY-13259) for another 4 h before harvesting. Next, the cells were lysed in denaturing buffer (1% SDS, 50 mM Tris-HCl, 0.5 mM EDTA, 1 mM DTT, pH 7.5). The lysates were incubated for 5 min at 100 °C immediately, and then sonicated and diluted with cell lysis buffer for Western and IP. Approximately 80% of the total lysates were immunoprecipitated with anti-Myc-conjugated magnetic beads (Bimake, B26302) for 2 h at room temperature, and the other lysates were used as input. The magnetic beads were then washed 3 times with PBST, and the immunoprecipitated proteins were eluted with 1 × SDS loading buffer at 100 °C for 5 min. The ubiquitination levels were detected using a Western Blot assay.

### Cell permeability experiments

786-O cells were seeded in 10 cm dish for 70–80% confluency and incubated with 20 µM 230D7 or 221C7 for 6 h. After washing 3 times with PBS, the cells were digested with 0.25% trypsin and lysed by 400 µL methanol. The cell lysates were vortexed and centrifuged at 16,260 × *g* for 30 min at 4 °C, and the supernatants were then processed and analyzed by LC-MS/MS system.

### Cell proliferation

Cells were seeded in 96-well plates and incubated with serially diluted compounds for 72 h. Cell viability was determined using the CellTiter-Glo® Luminescent Cell Viability Assay kit (Promega, G7573) following the manufacturer’s instructions. IC_50_ values were determined by nonlinear regression (curve fit) using a variable slope (four parameters) in GraphPad Prism (9.0).

### Animals

All procedures performed on animals were in accordance with regulations and established guidelines and were reviewed and approved by the Institutional Animal Care and Use Committee at the Shanghai Institute of Materia Medica, Chinese Academy of Sciences (IACUC Issue NO. 2022-01-JHL-27 for NSG mice; IACUC Issue NO. 2021-03-JHL-22 for BALB/c mice). NSG mice were obtained from Shanghai Model Organisms Center, Inc; BALB/c mice were obtained from Beijing Huafukang Biotechnology Co. Ltd (Beijing, China). Six- to eight-week-old mice were used for the studies and were maintained with free access to pellet food and water in plastic cages at 21 ± 2 °C and humidity (50 ± 10%) conditions and kept on a 12 h light/dark cycle. The tumor size tolerated by the xenograft tumor model mice did not exceed 2000 mm^3^, the maximal tumor burden permitted by the Institutional Animal Care and Use Committee at the Shanghai Institute of Materia Medica, Chinese Academy of Sciences.

### Pharmacokinetics

The pharmacokinetic profiles of compound 230D7 were determined in male BALB/c mice. The test compound 230D7 was dissolved in solution containing DMSO, PEG400, PBS (5/5/90, v/v) and administered via intraperitoneal administration (i.p.) at 10 mg/kg. Serial blood samples (50–100 µL) were collected at 0.25, 0.5, 1, 2, 4, 8, 24 h after dosing and centrifuged at 7227 × *g* for 5 min to obtain the plasma fraction. A 10 μL aliquot of plasma was deproteinized with 100 μL acetonitrile/methanol (1/1, v/v) containing internal standard. After centrifugation, the supernatant was diluted with a certain proportion of acetonitrile/water (1/1, v/v), mixed and centrifuged at 1807 × *g* for 10 min. Finally, the aliquots of the diluted supernatant were injected into LC–MS/MS system.

### Acute toxicity

BALB/c mice were used to evaluate the toxicity of compound 230D7. The mice were randomly divided into 3 groups (*n* = 3) and treated with different doses of compound 230D7 (0, 50, 100 mg/kg) by intraperitoneal administration daily for a week. The body weights of mice were measured every day and the significant organs (heart, kidney, lung, liver and spleen) were harvested, weighted and used for histological analysis at the last day.

### H&E staining

For histological analysis of BALB/c mice in 230D7-treated or vehicle control groups, H&E staining were performed using standard histological techniques. According to the manufacturer’s protocol (Servicebio, Inc.), isolated organ tissues were fixed in 4% neutral paraformaldehyde for 24 h and embedded in paraffin wax. Paraffin slides (4 μm) were then dewaxed and hydrated. Subsequently, the slides were sequentially stained with hematoxylin and eosin. Lastly, the slides were sealed with neutral resin and images were captured by microscopy (Eclipse E100, DS-U3, Nikon).

### 786-O cells xenograft tumor growth

NSG mice were used to evaluate the pharmacodynamics of 230D7. The 786-O cells xenograft tumor model was established by the subcutaneous injection of 786-O cells (1 × 10^7^) into the NSG mice. When the tumor reached the volume of approximately 100 mm^3^, the mice were randomly divided into three groups (*n* = 7) and intraperitoneally treated with different dosages of 230D7 (0, 25, 50 mg/kg, 230D7 was synthesized in our laboratory) in solution containing DMSO, PBS (5/90, v/v) once a day for 16 days. Body weight and tumor size were measured every 2 or 3 days, and tumor volume was calculated using the formula: *V* = (*L* × *W*^2^)/2 (*L*, length; *W*, width). At the end of the experiment, the mice were euthanized and the tumors were harvested for Western Blot and other studies.

### CT26 cells transplanted tumor growth

BALB/c mice were used to evaluate the pharmacodynamics of rabeprazole (purchased from MedChemExpress, HY-B0656). The CT26 cells transplanted tumor model was established by the subcutaneous injection of CT26 cells (2.5 × 10^5^) into mice. When the tumor reached the volume of approximately 100 mm^3^, the mice were randomly divided into two groups (*n* = 5) and intraperitoneally treated with rabeprazole (0, 40 mg/kg) in solution containing DMSO, PEG300, PBS, (1/10/89, v/v/v) once a day for 10 days. The tumor size was recorded using callipers, and tumor volume was calculated using the formula: *V* = (*L* × *R*^2^)/2. At the end of the experiment, the mice were euthanized and the tumors were harvested for immunohistochemistry and fluorescence-activated cell sorting.

### Nile red staining

CT26 cells were seeded into Lab-Tek^TM^ II Chamber Slide systems (Thermo) and incubated with rabeprazole or vehicle. Then, the cells were washed with PBS for 15 min, fixed with Immunol Staining Fix Solution (Beyotime, P0098) for 30 min and washed with PBS again, followed by treatment with Immunostaining Permeabilization Buffer with Triton X-100 (Beyotime, P0098) for 30 min and washing with PBS again. To stain the lipid droplets, the cells were incubated with Nile Red (2 µM) in the dark for 10–30 min and then washed with PBS before the nuclei were stained with Antifade Mounting Medium with DAPI (Beyotime, P0131). Fluorescence images were captured using an OLYMPUS IX73 fluorescence microscope and Lecia two-photon confocal microscope.

### Immunohistochemistry (IHC)

The isolated CT26 tumor tissue was fixed with neutral paraformaldehyde, and subsequent staining of cell surface markers was performed by Servicebio Company (Wuhan, China). In brief, the tumor tissue embedded in paraffin was processed through sectioning, dewaxing, rehydration, and antigen retrieval. Following peroxidase inactivation and blocking with goat serum, the tissue was incubated overnight with the corresponding primary monoclonal antibody overnight at 4 °C. The next day, slides were washed three times and incubated with horseradish peroxidase (HRP)-linked secondary antibodies for 1 h at room temperature. Specimens were washed three times then developed with the DAB substrate kit and counterstained with haematoxylin.

### Fluorescence-activated cell sorting (FACS)

We analyzed the infiltration of immune cell subsets in CT26 transplanted tumor tissue by fluorescence-activated cell sorting (FACS) analysis. After the mice were euthanized, the tumor tissues were stripped and cut into pieces, then digested at 37 °C for 60 min with tumor tissue digestive buffer (0.1% collagenase, 0.001% hyaluronidase, 0.002% DNA enzyme, 120 μM CaCl_2_ and 120 μM MgCl_2_ in RPMI1640 medium). The digested tumor tissues were filtered with 200 mesh gauze, followed by the lysis of red blood cells with ammonium chloride solution, and filtered again to obtain single-cell suspensions in PBS. For discriminating the living and dead cells, 1 × 10^6^ cells were stained on ice with Fixable Viability Stain 700 (BD Horizan, 564997) for 10 min and then terminated with cell staining buffer (PBS containing 2% FBS). Fc receptors on the cell surface are blocked by 1 μg anti-Mouse CD16/32 antibody (10 min on ice). Then, appropriately conjugated fluorescent primary antibodies were added to stain cell surface markers. Finally, cells were suspended with cell staining buffer for flow cytometry analysis using Beckman CytoFelx. The following antibodies were used: anti-CD3 (Invitrogen, 11-0032-82, 1:1000), anti-CD8 (Biolegend, 100738, 1:1000), anti-PD1 (Biolegend, 135219, 1:1000), anti-TIM3 (Invitrogen, 12-5870-82, 1:1000). The data were analyzed by Flowjo software and cell populations were defined as shown in Supplementary Fig. 7.

### Statistical analysis and reproducibility

GraphPad Prism 9.0 software was used to perform statistical analysis. Differences of quantitative data between groups were calculated using 2-tailed unpaired *t*-test. The significance level was set as *P < 0.05, **P < 0.01, ^***^P < 0.001, ****P < 0.0001.

### Synthesis of 230D7 and 222A5

All reagents and solvents, unless otherwise specified, were purchased from commercial sources and used without further purification. ^1^H NMR and ^13^C NMR spectra were recorded on Mercury-600 spectrometers at room temperature. Chemical shifts are referenced to the residual solvent peak and reported in ppm (*δ* scale), and all coupling constant (*J*) values are given in Hz. ESI-HRMS and ESI-LRMS data were measured on Thermo Exactive Orbitrap plus spectrometer. Flash column chromatography was performed on Flash 300 Isolera one. Analytical HPLC conditions were as follows: Agilent 1260 Infinity II variable wavelength detector; Waters XBridge C18, 4.6 mm × 150 mm, 3.5 μm particles. Phase A was water with 0.1% TFA, and phase B was MeCN. The entire eluting time was 10 min with a gradient from 10% phase B to 90% phase B in 3.5 min, followed by a 4.5 min hold at 90% phase B, and then a gradient from 90% phase B to 10% phase B in the next 2 min. The flow rate was 1 mL/min. The synthetic routes of the compounds are shown in Supplementary Fig. 8. ^1^H NMR, ^13^C NMR, HRMS and HPLC data of intermediates and final products are reported in Supplementary Figs. 9–24.

#### Ethyl 5-((4-bromo-2-chlorophenoxy)methyl)furan-2-carboxylate (**1**)

To a solution of 4-bromo-2-chlorophenol (1.0 g, 4.9 mmol, 1.0 eq), ethyl 5-(chloromethyl)furan-2-carboxylate (0.92 g, 4.9 mmol, 1.0 eq) in DMF (7 mL) was added K_2_CO_3_ (1.1 g, 8.0 mmol, 1.6 eq), the mixture was stirred at 60 °C for 5 h. After completion of the reaction, H_2_O (50 mL) was added and the mixture was extracted with EtOAc (50 mL × 3). The combined organic phases were washed with brine (30 mL × 3), dried over Na_2_SO_4_, and concentrated in vacuum. The residue was purified by column chromatography using 20% EtOAc in hexane to obtain the title compound **1** (1.2 g, 69%) as a white solid. ^1^H NMR (600 MHz, DMSO-*d*_6_) δ 7.70 (d, *J* = 2.4 Hz, 1H), 7.53 (dd, *J* = 9.0, 2.4 Hz, 1H), 7.32-7.27 (m, 2H), 6.82 (d, *J* = 3.6 Hz, 1H), 5.28 (s, 2H), 4.29 (q, *J* = 7.2 Hz, 2H), 1.29 (t, *J* = 7.2 Hz, 3H); ^13^C NMR (151 MHz, DMSO-*d*_6_) δ 158.23, 154.02, 153.04, 144.79, 132.59, 131.47, 123.40, 119.38, 116.67, 113.47, 113.09, 63.13, 61.24, 14.63; HRMS (m/z): [M + H]^+^ calcd. for C_14_H_13_BrClO_4_, 358.9680; found, 358.9680; HPLC: purity 98.9%, retention time 9.221 min.

#### 5-((4-bromo-2-chlorophenoxy)methyl)furan-2-carboxylic acid (**2**)

To a solution of **1** (1.2 g, 3.4 mmol, 1.0 eq) in MeOH (15 mL) was added 2 M aqueous NaOH (15 mL, 30 mmol, 8.8 eq), and the mixture was stirred at 50 °C for 2 h. After completion of the reaction, the mixture was concentrated under reduced pressure, then the residue was acidified to pH 4 by the dropwise addition of concentrated HCl at 0 °C. The precipitated solid was filtered to afford the title compound **2** (1.09 g, 97%) as a white solid. ^1^H NMR (600 MHz, DMSO-*d*_6_) δ 13.23 (s, 1H), 7.70 (d, *J* = 2.4 Hz, 1H), 7.53 (dd, *J* = 9.0, 2.4 Hz, 1H), 7.30 (d, *J* = 9.0 Hz, 1H), 7.22 (d, *J* = 3.6 Hz, 1H), 6.79 (d, *J* = 3.6 Hz, 1H), 5.26 (s, 2H); ^13^C NMR (151 MHz, DMSO-*d*_6_) δ 159.64, 153.54, 153.07, 145.82, 132.59, 131.47, 123.39, 118.82, 116.65, 113.36, 113.04, 63.18; HRMS (m/z): [M-H]^-^ calcd. for C_12_H_7_BrClO_4_, 328.9222; found, 328.9223; HPLC: purity 99.3%. retention time 8.061 min.

#### 5-((4-bromo-2-chlorophenoxy)methyl)furan-2-carbonyl chloride (**3**)

A solution of **2** (0.20 g, 0.61 mmol, 1.0 eq) in SOCl_2_ (3.0 mL, 41.3 mmol, 67.8 eq) was stirred under reflux for 2 h. After being cooled to rt, the solution was concentrated under reduced pressure to remove the excess SOCl_2_. The residue was then dried under high vacuo for 1 h, and the crude product **3** (0.21 g) as a white solid was used directly for the next step without further purification.

#### (6*R*,7*R*)−3-(acetoxymethyl)−7-(5-((4-bromo-2-chlorophenoxy)methyl)furan-2-carboxamido)−8-oxo-5-thia-1-azabicyclo[4.2.0]oct-2-ene-2-carboxylic acid (**230D7**)

To a solution of (6*R*,7*R*)−3-(acetoxymethyl)−7-amino-8-oxo-5-thia-1-azabicyclo[4.2.0]oct-2-ene-2-carboxylic acid (0.21 g, 0.77 mmol, 1.0 eq) in acetone (7 mL) was added saturated aqueous NaHCO_3_ (14 mL), and then **3** (0.32 g, 0.91 mmol, 1.2 eq) in acetone (5 mL) was added dropwise at 0 °C over 15 min. The reaction mixture was allowed to warm to rt and stirred for 4 h. After completion of the reaction, the pH was adjusted to 4 with 1 M aqueous HCl. The precipitated solid was filtered to obtain a crude product. The crude product was purified by column chromatography using 5% MeOH in DCM, to afford the title compound **230D7** (0.30 g, 66%) as a white solid. ^1^H NMR (600 MHz, DMSO-*d*_6_) *δ* 13.71 (s, 1H), 9.36 (d, *J* = 8.4 Hz, 1H), 7.70 (d, *J* = 2.4 Hz, 1H), 7.52 (dd, *J* = 9.0, 2.4 Hz, 1H), 7.38 (d, *J* = 3.6 Hz, 1H), 7.31 (d, *J* = 9.0 Hz, 1H), 6.78 (d, *J* = 3.6 Hz, 1H), 5.82 (dd, *J* = 7.8, 4.8 Hz, 1H), 5.24 (s, 2H), 5.18 (d, *J* = 4.8 Hz, 1H), 4.99 (d, *J* = 12.8 Hz, 1H), 4.71 (d, *J* = 12.8 Hz, 1H), 3.65 (d, *J* = 18.0 Hz, 1H), 3.51 (d, *J* = 18.0 Hz, 1H), 2.04 (s, 3H); ^13^C NMR (151 MHz, DMSO-*d*_*6*_) δ 170.67, 164.27, 163.31, 158.22, 153.11, 152.64, 147.15, 132.58, 131.47, 127.03, 123.76, 123.44, 116.71, 116.06, 113.30, 113.03, 63.20, 63.14, 59.67, 58.05, 26.04, 21.03; HRMS (m/z): [M+Na]^+^ calcd. for C_22_H_18_BrClN_2_NaO_8_S, 606.9548; found, 606.9565; HPLC: purity 98.6%, retention time 8.023 min.

#### (6*R*,7*R*)−7-(3-cyclopentylpropanamido)−8-oxo-3-vinyl-5-thia-1-azabicyclo[4.2.0]oct-2-ene-2-carboxylic acid (**222A5**)

To a solution of (6*R*,7*R*)−7-amino-8-oxo-3-vinyl-5-thia-1-azabicyclo[4.2.0]oct-2-ene-2-carboxylic acid (0.20 g, 0.88 mmol, 1.0 eq) in acetone (5 mL) was added saturated aqueous NaHCO_3_ (10 mL), followed by dropwise addition of 3-cyclopentylpropanoyl chloride (0.15 μL, 0.97 mmol, 1.1 eq) in acetone (5 mL) at 0 °C over 15 min. The reaction mixture was allowed to warm to rt and stirred for 4 h. After completion of the reaction, the pH was adjusted to 4 with 1 M aqueous HCl, the precipitated solid was filtered to afford a crude product. The crude product was purified by column chromatography using 5% MeOH in DCM to afford the title compound **222A5** (0.15 g, 44%) as a white solid. ^1^H NMR (600 MHz, DMSO-*d*_*6*_) δ 8.85 (d, *J* = 8.4 Hz, 1H), 6.90 (dd, *J* = 17.4, 11.4 Hz, 1H), 5.66 (dd, *J* = 8.4, 4.8 Hz, 1H), 5.59 (d, *J* = 17.4 Hz, 1H), 5.31 (d, *J* = 11.4 Hz, 1H), 5.12 (d, *J* = 4.8 Hz, 1H), 3.86 (d, *J* = 17.7 Hz, 1H), 3.56 (d, *J* = 17.6 Hz, 1H), 2.26-2.13 (m, 2H), 1.76-1.67 (m, 3H), 1.60-1.40 (m, 6H), 1.10-1.00 (m, 2H); ^13^C NMR (151 MHz, DMSO-*d*_*6*_) δ 173.64, 165.23, 163.72, 132.46, 125.93, 124.49, 117.69, 59.55, 58.25, 39.58, 34.56, 32.53, 32.37, 31.98, 25.19, 25.14, 23.51; HRMS (m/z): [M + Na]^+^ calcd. for C_17_H_22_N_2_NaO_4_S, 373.1192; found, 373.1196; HPLC: purity 97.5%, retention time 7.662 min.

### Reporting summary

Further information on research design is available in the Nature Portfolio Reporting Summary linked to this article.

## Supplementary information

Supplementary information

Reporting Summary

## Data Availability

The biological data generated in this study have been deposited in the Figshare database under accession code 10.6084/m9.figshare.23567292. The raw data in this study are provided in the Source Data file. The virtual screening results and spectral data for new compounds are available in the Supplementary Information. ChEMBL database is available at https://www.ebi.ac.uk/chembl/, and DrugBank dataset is available at https://go.drugbank.com/. The commercial Chemspace Library is available at https://chem-space.com/, and ChemDiv Library is available at https://www.chemdiv.com/. All data are available from the corresponding author upon request. Source data are provided with this paper.

## Source data

Source data

## Code availability

The inference and interpretation codes of TransformerCPI2.0 are available at https://github.com/lifanchen-simm/transformerCPI2.0/. The inference and interpretation codes of TransformerCPI2.0 have been deposited in the Zenodo under accession code 10.5281/zenodo.7993486.
