# Supplementary material for: Sequence-based drug design as a concept in computational drug design
Source: Nat Commun. 2023 Jul 14;14:4217. doi: 10.1038/s41467-023-39856-w (PMC10349078; doi:10.1038/s41467-023-39856-w)
Supplement: Supplementary file 1 — Supplementary information [file 41467_2023_39856_MOESM1_ESM.pdf]

Supplementary Information for

Sequence-based drug design as a concept in computational drug design

Lifan Chen, Zisheng Fan, Jie Chang, Ruirui Yang, Hui Hou, Hao Guo, Yinghui Zhang, Tianbiao Yang, Chenmao Zhou, Qibang Sui, Zhengyang Chen, Chen Zheng, Xinyue Hao, Keke Zhang, Rongrong Cui, Zehong Zhang, Hudson Ma, Yiluan Ding, Naixia Zhang, Xiaojie Lu, Xiaomin Luo, Hualiang Jiang, Sulin Zhang, Mingyue Zheng.

Correspondence to: myzheng@simm.ac.cn (Mingyue Zheng), slzhang@simm.ac.cn (Sulin Zhang)

Supplementary Fig. 1~24

Supplementary Tab. 1~18

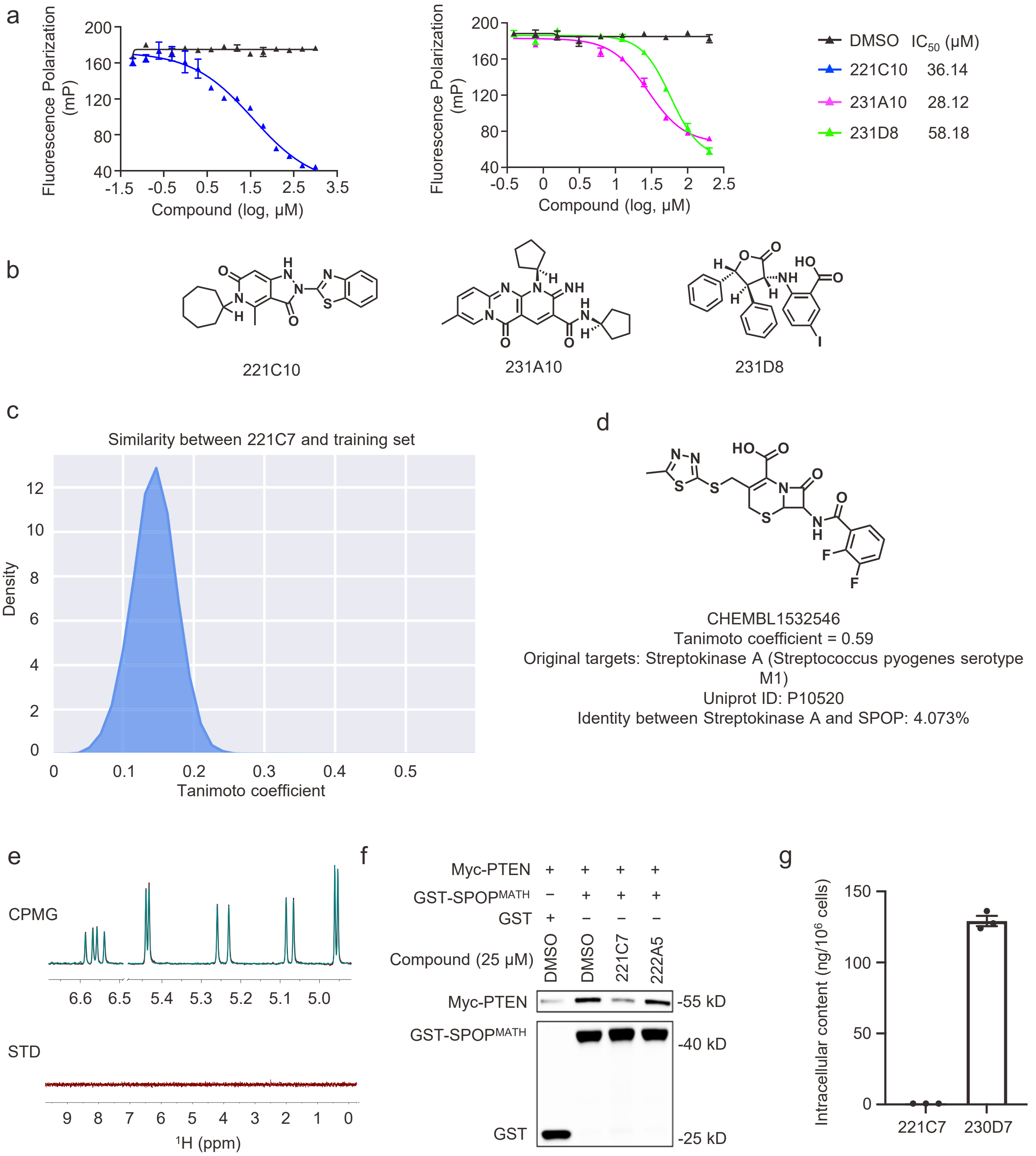
a
Fluorescence Polarization (mP)
Compound (log, μM)
DMSO IC50 (μM)
221C10 36.14
231A10 28.12
231D8 58.18
b
221C10
231A10
231D8
c
Similarity between 221C7 and training set
Density
Tanimoto coefficient
d
CHEMBL1532546
Tanimoto coefficient = 0.59
Original targets: Streptokinase A (Streptococcus pyogenes serotype M1)
Uniprot ID: P10520
Identity between Streptokinase A and SPOP: 4.073%
e
CPMG
STD
1H (ppm)
f
Myc-PTEN
GST-SPOPMATH
GST
Compound (25 μM)
DMSO
DMSO
221C7
222A5
-55 kD
-40 kD
-25 kD
g
Intracellular content (ng/10^6 cells)
221C7
230D7

Supplementary Fig. 1. Supplementary data for Fig. 4. a, The FP assay of three initial hits predicted by TransformerCPI2.0. Error bars represent mean ± SEM of two independent experiments. **b**, Chemical structures of the three other initial hits predicted by TransformerCPI2.0. **c**, Tanimoto similarity between 221C7 and training set compounds calculated by ECFP fingerprints. The most compounds in the training set have low similarity coefficient with 221C7. **d**, We investigated the most similar compounds in the training set, which has Tanimoto coefficient of 0.59. The original target of this compound is Streptokinase A from Streptococcus, and the sequence identity between Streptokinase A and SPOP is 4.073%. **e**, CPMG NMR spectra for 222A5 (red), 222A5 in the presence of 5 μM $SPOP^{MATH}$ (green). The STD spectrum for 222A5 is recorded in the presence of 5 μM $SPOP^{MATH}$. **f**, The ability of 221C7 and 222A5 to disrupt the binding of $SPOP^{MATH}$ to PTEN was determined by the *in vitro* pull-down assay. This experiment is repeated three times independently with similar results. **g**, Cell permeability measurements of 221C7 and 230D7. After treating 786-O cells with 20 μM of the indicated compounds for 6 hours, the intracellular content of 221C7 and 230D7 were measured by LC-MS/MS. Error bars represent mean ± SEM of three independent experiments. Source data are provided as a Source Data file.

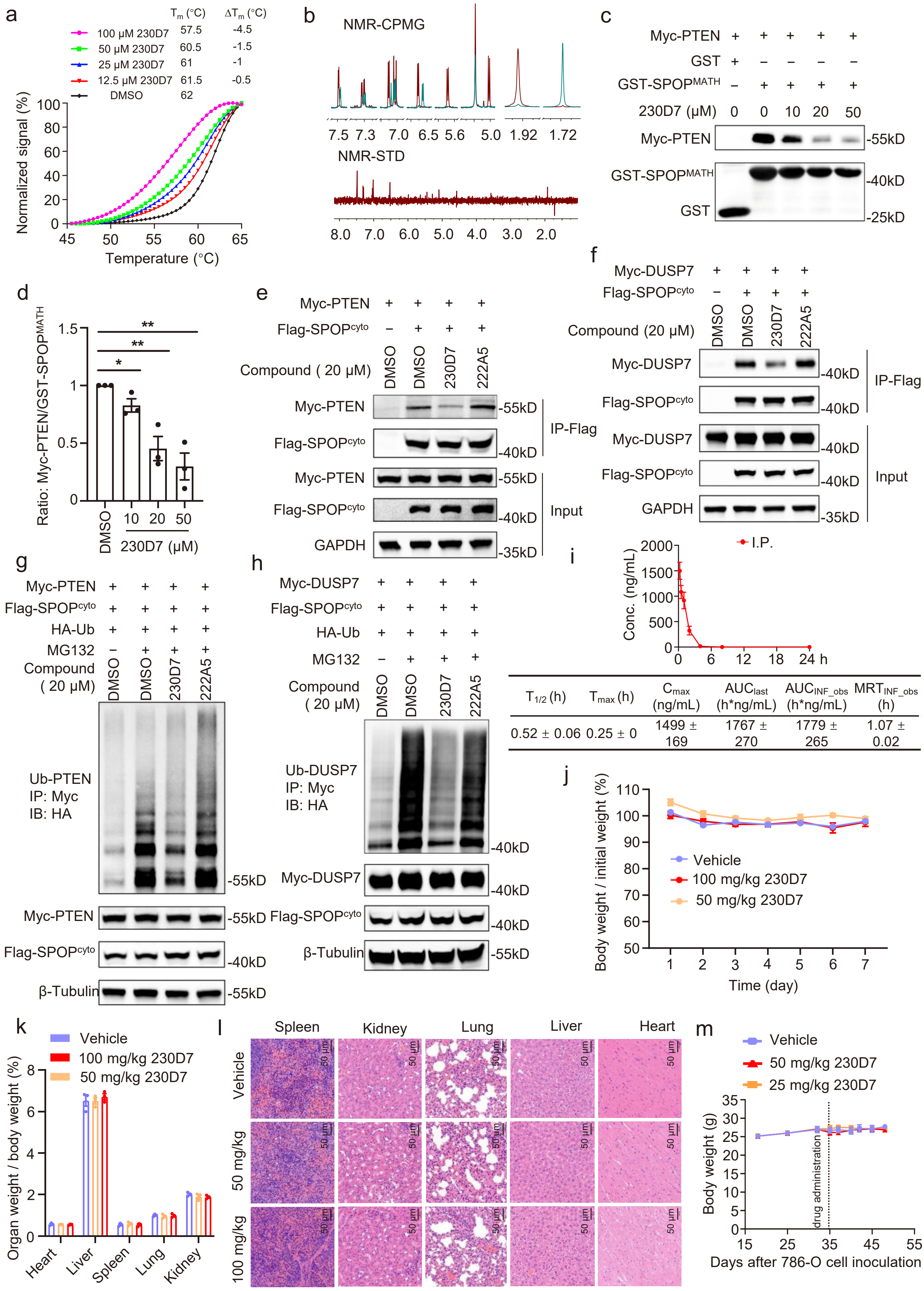

$T_{1/2}$ (h)	T_{max} (h)	C_{max} (ng/mL)	AUC_{last} (h*ng/mL)	AUC_{INF_obs} (h*ng/mL)	MRT_{INF_obs} (h)
0.52 ± 0.06	0.25 ± 0	1499 ± 169	1767 ± 270	1779 ± 265	1.07 ± 0.02

Supplementary Fig. 2. Supplementary data for Fig. 5. a, Thermostability of $SPOP^{MATH}$ (5 μM) treated with different concentrations of 230D7. The thermal stability of $SPOP^{MATH}$ was quantified by the **Δ**T_m. **b**, NMR measurement of direct binding between 230D7 and $SPOP^{MATH}$. CPMG NMR spectra for 230D7 (red), 230D7 in the presence of 5 μM $SPOP^{MATH}$ (green). The STD spectrum for 230D7 is recorded in the presence of 5 μM $SPOP^{MATH}$. **c**, 230D7 disrupts protein binding between $SPOP^{MATH}$ and PTEN, as measured by *in vitro* pull-down assay. This experiment is repeated three times independently with similar results. **d**, The gray values of Myc-PTEN and GST-$SPOP^{MTAH}$ protein bands in (c) were quantified, and the ratio of Myc-PTEN/GST-$SPOP^{MTAH}$ were calculated. Error bars represent mean ± SEM of three independent experiments. *P* values were evaluated using 2-tailed unpaired t-test. *$P < 0.05$, **$P < 0.01$. (10 μM 230D7 vs. DMSO, P = 0.0389; 20 μM 230D7 vs. DMSO, P = 0.0065; 50 μM 230D7 vs. DMSO, P = 0.0038.) **e~f**, Inhibitory activities of 230D7 and negative control compound 222A5 on the binding of SPOP-PTEN (**e**) or SPOP-DUSP7 (**f**) in the coimmunoprecipitation experiments. These experiments are repeated twice independently with similar results. **g~h**, Effects of 230D7 and negative control compound 222A5 on the ubiquitination level of PTEN (**g**) or DUSP7 (**h**) in the *in vivo* ubiquitination experiments. These experiments are repeated twice independently with similar results. **i**, Concentrations (ng/mL) of 230D7 in BALB/c mice plasma after i.p. administration of 10 mg/kg 230D7. The Pharmacokinetic parameters were summarized in the table. Error bars represent mean ± SEM of three biologically independent animals. **j**, The body weight of BALB/c mice treated with different dosages of 230D7 daily for 7 days. Error bars represent mean ± SEM of three biologically independent animals. **k**, The weight of different organs (heart, liver, spleen, lung, and kidney) of BALB/c mice treated with different dosages of 230D7 daily for 7 day. Error bars represent mean ± SEM of three biologically independent animals. **l**, Representative histological morphology of H&E-stained tissue sections of BALB/c mice in 230D7-treated or vehicle control groups. **m**, The body weight of NSG mice were measured during the entire pharmacodynamics study of 230D7. Error bars represent mean ± SEM of seven biologically independent animals. Source data are provided as a Source Data file.

230D7
413 Assays Tested
0 Interactions Mapped

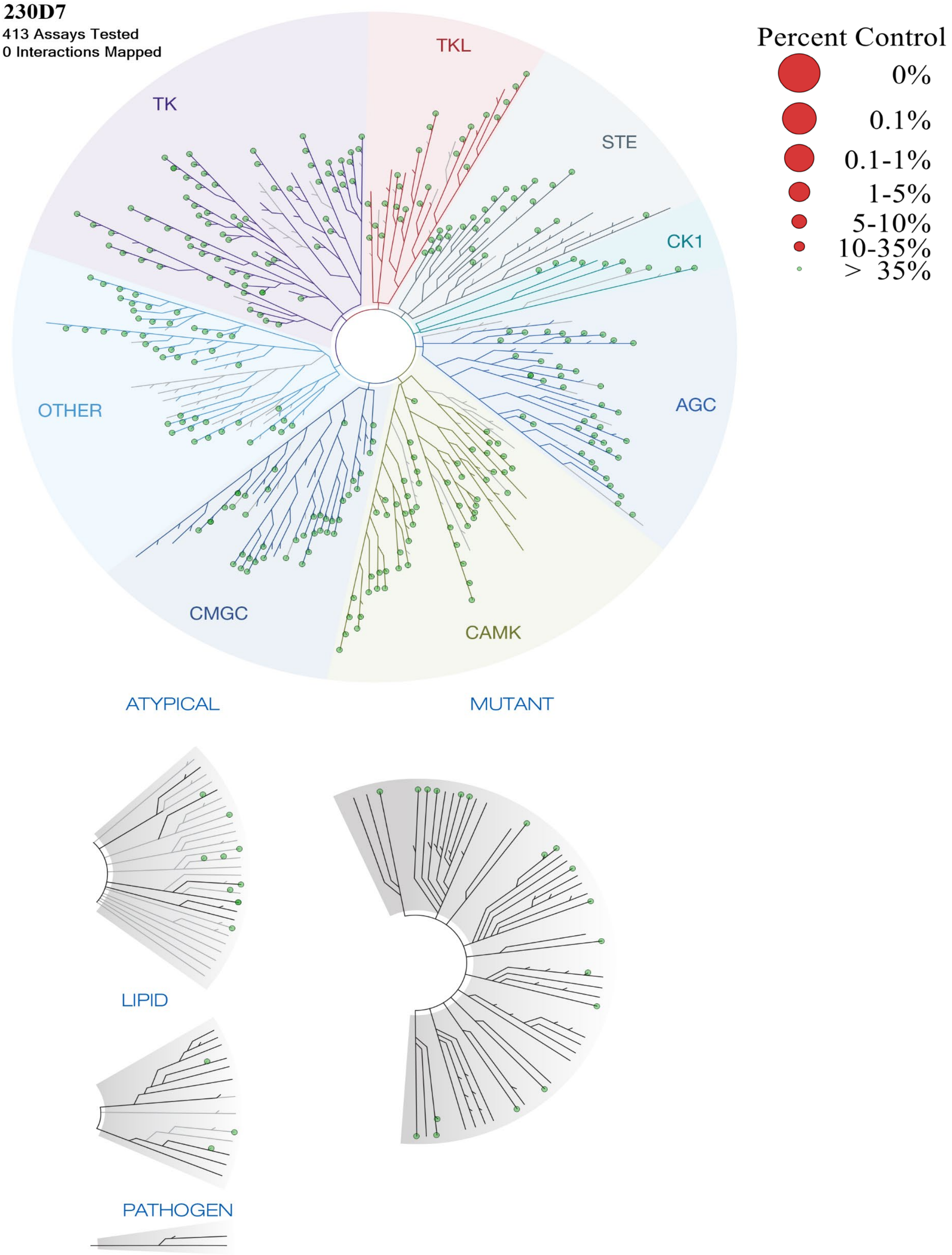

Supplementary Fig. 3. Kinome profiling of 230D7. 230D7 (10 μM) was submitted for a KinaseProfiler (eurofins) to quantify interactions with 413 human wild-type/mutant kinases. The results are displayed as a TREESPOT interaction map. Image generated using TREEspot™ Software Tool and reprinted with permission from KINOMEscan®, a division of DiscoveRx Corporation, © DISCOVERX CORPORATION 2010. Source data are provided as a Source Data file.

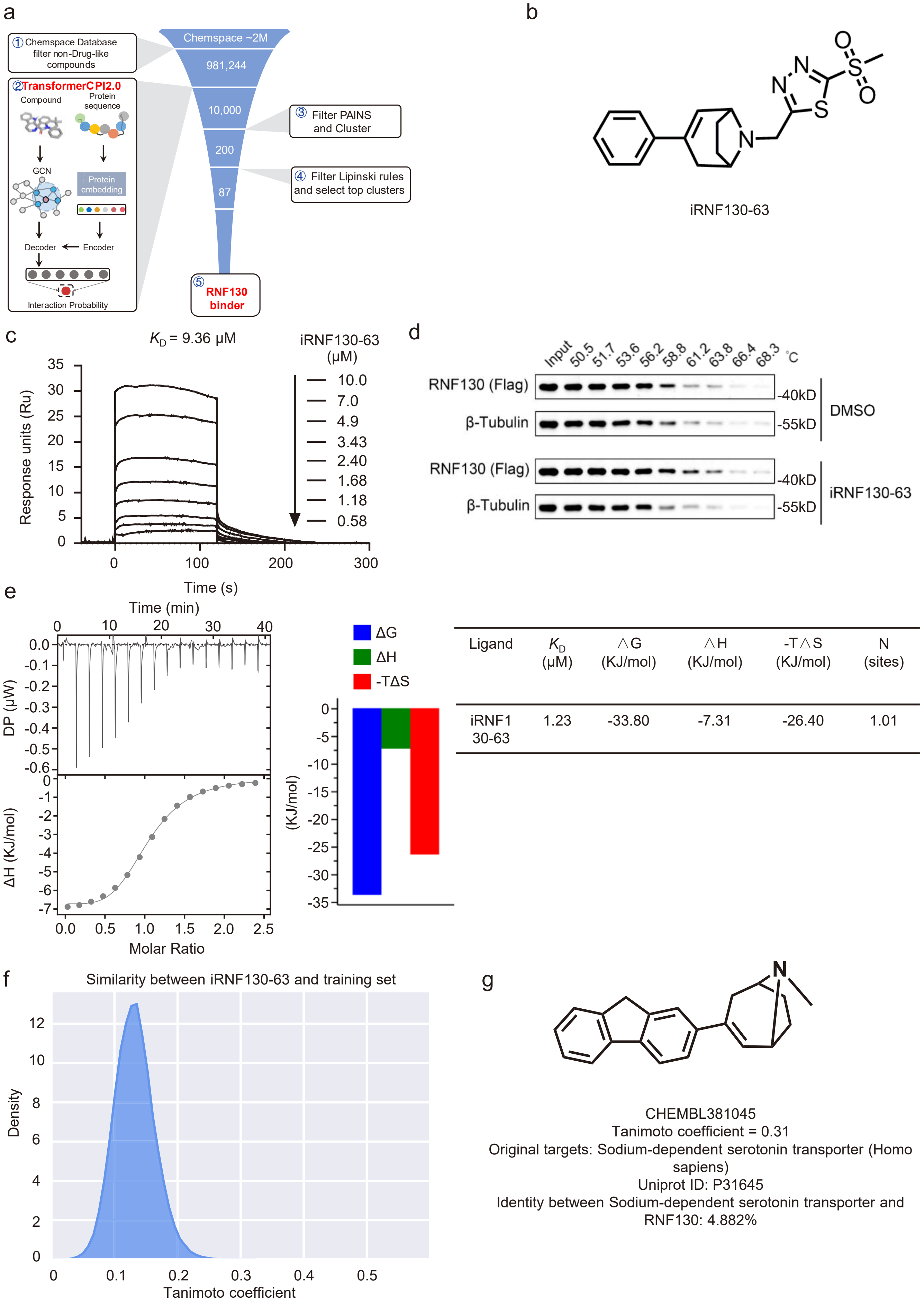

Ligand	K_D (μM)	△G (KJ/mol)	△H (KJ/mol)	-T△S (KJ/mol)	N (sites)
iRNF130-63	1.23	-33.80	-7.31	-26.40	1.01

Supplementary Fig. 4. Discovering the chemical binder of RNF130. **a**, The virtual screening procedure of RNF130. **b**, Chemical structure of iRNF130-63. **c**, Surface plasmon resonance analysis examining the direct binding affinity of iRNF130-63 to RNF130. Graphs of equilibrium unit responses versus iRNF130-63 concentrations are plotted. **d**, Representative western blots for the effect of 50 μM iRNF130-63 on the thermal stabilization of RNF130 protein. Cellular thermal shift assay (CETSA) was assayed in 293T cell lysate. These experiments are repeated twice independently with similar results. **e**, Binding of iRNF130-63 with RNF130 was characterized by isothermal titration calorimetry (ITC). Thermodynamic parameters of iRNF130-63 measured by ITC are shown in the table. **f**, Tanimoto similarity between iRNF130-63 and training set compounds calculated by ECFP fingerprints. The most compounds in the training set have low similarity coefficient with iRNF130-63. **g**, We investigated the most similar compounds in the training set, which has Tanimoto coefficient of 0.31. The original target of this compound is Sodium-dependent serotonin transporter from Homo sapiens, and the sequence identity between Sodium-dependent serotonin transporter and RNF130 is 4.882%. Source data are provided as a Source Data file.

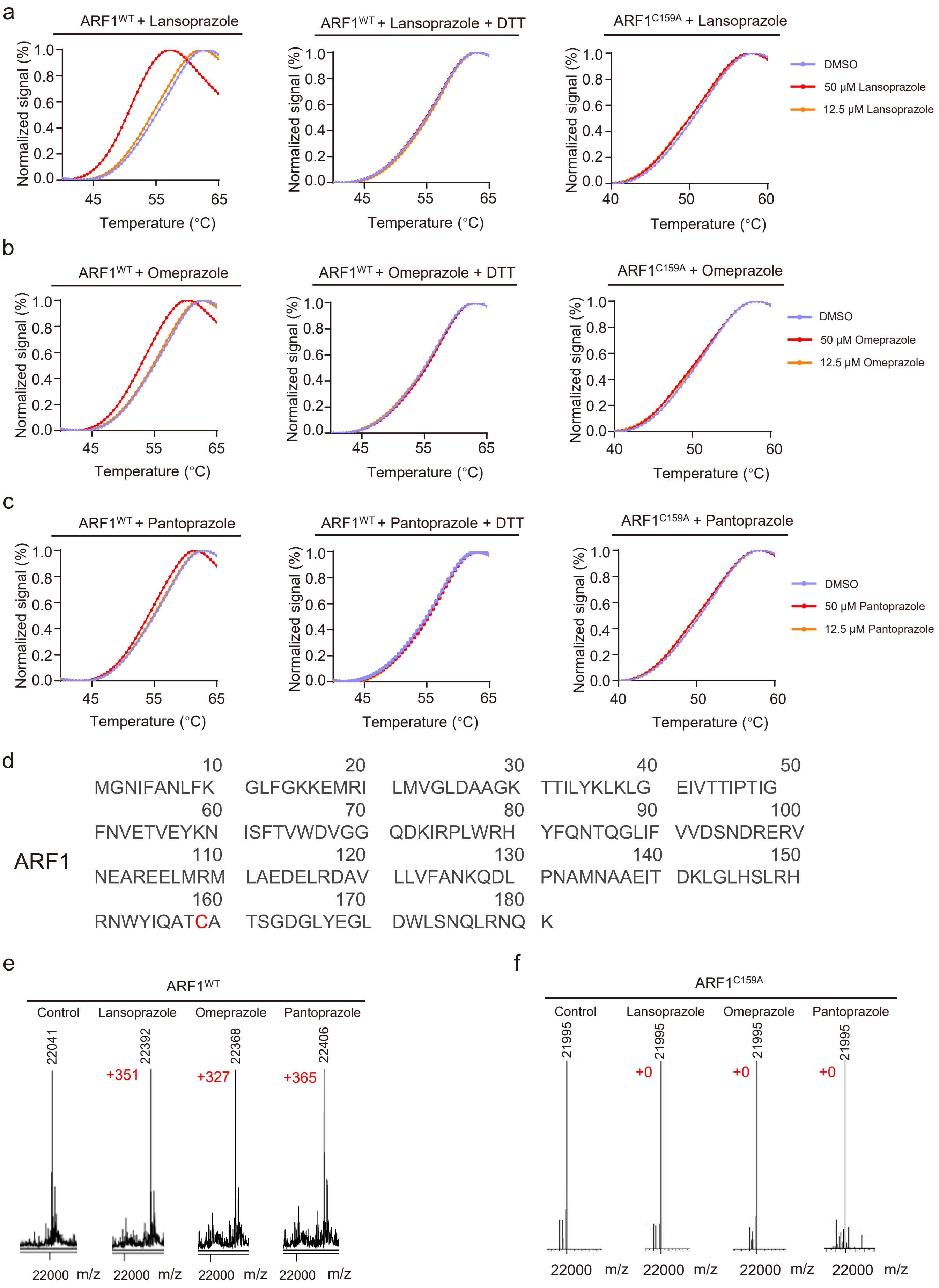
a
ARF1WT + Lansoprazole
ARF1WT + Lansoprazole + DTT
ARF1C159A + Lansoprazole
Normalized signal (%)
Temperature (°C)
DMSO
50 μM Lansoprazole
12.5 μM Lansoprazole
b
ARF1WT + Omeprazole
ARF1WT + Omeprazole + DTT
ARF1C159A + Omeprazole
DMSO
50 μM Omeprazole
12.5 μM Omeprazole
c
ARF1WT + Pantoprazole
ARF1WT + Pantoprazole + DTT
ARF1C159A + Pantoprazole
DMSO
50 μM Pantoprazole
12.5 μM Pantoprazole
d
ARF1
10 20 30 40 50
MGNIFANLFK GLFGKKEMRI LMVGLDAAGK TTILYKLKLG EIVTTIPTIG
60 70 80 90 100
FNVETVEYKN ISFTVWDVGG QDKIRPLWRH YFQNTQGLIF VVDSNDRERV
110 120 130 140 150
NEAREELMRM LAEDELRDAV LLVFANKQDL PNAMNAAEIT DKLGLHSLRH
160 170 180
RNWYIQATCA TSGDGLYEGL DWLSNQLRNQ K
e
ARF1WT
Control
Lansoprazole
Omeprazole
Pantoprazole
22041
22392
+351
22368
+327
22406
+365
22000 m/z
f
ARF1C159A
Control
Lansoprazole
Omeprazole
Pantoprazole
21995
+0
22000 m/z

Supplementary Fig. 5. Supplementary data for Fig 6. a, PTS assay of lansoprazole (12.5 or 50 μM) + $ARF1^{WT}$ (2.5 μM) (left panel); PTS assay of lansoprazole (12.5 or 50 μM) + $ARF1^{WT}$ (2.5 μM) + DTT (middle panel); PTS assay of $ARF1^{C159A}$ (2.5 μM) + lansoprazole (12.5 or 50 μM) (right panel). **b**, PTS assay of omeprazole (12.5 or 50 μM) + $ARF1^{WT}$ (2.5 μM) (left panel); PTS assay of omeprazole (12.5 or 50 μM) + $ARF1^{WT}$ (2.5 μM) + DTT (middle panel); PTS assay of $ARF1^{C159A}$ (2.5 μM) + omeprazole (12.5 or 50 μM) (right panel). **c**, PTS assay of pantoprazole (12.5 or 50 μM) + $ARF1^{WT}$ (2.5 μM) (left panel); PTS assay of pantoprazole (12.5 or 50 μM) + $ARF1^{WT}$ (2.5 μM) + DTT (middle panel); PTS assay of $ARF1^{C159A}$ (2.5 μM) + pantoprazole (12.5 or 50 μM) (right panel). **d**, Amino acid sequence of ARF1, and C159 (marked red) is the only cysteine residue. **e**, Deconvoluted electrospray ionization mass spectra of $ARF1^{WT}$ in the presence of lansoprazole or omeprazole or pantoprazole. **f**, Deconvoluted electrospray ionization mass spectra of $ARF1^{C159A}$ in the presence of lansoprazole or omeprazole or pantoprazole. Source data are provided as a Source Data file.

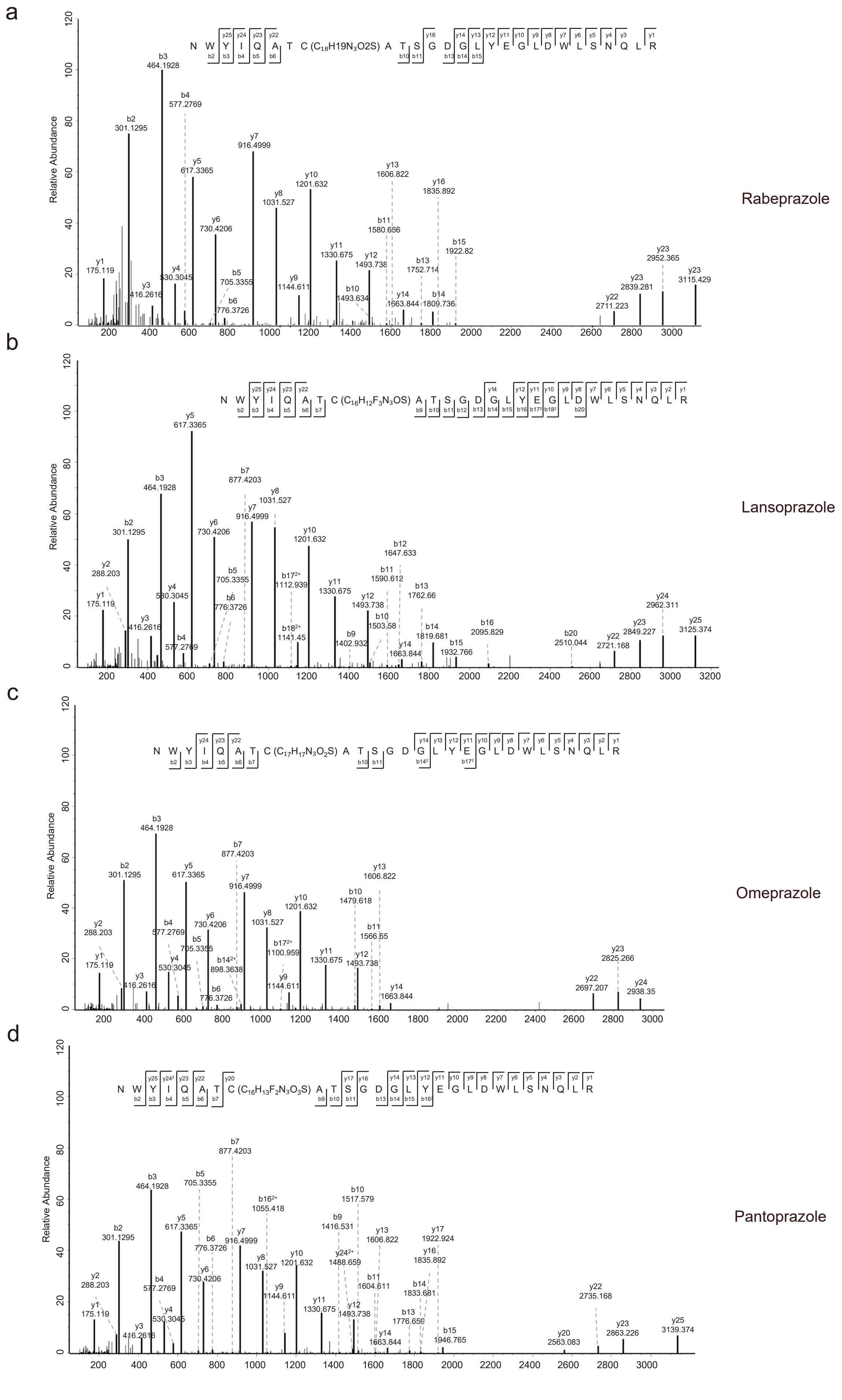
a
N W Y I Q A T C (C18H19N3O2S) A T S G D G L Y E G L D W L S N Q L R
Rabeprazole
b
N W Y I Q A T C (C16H12F3N3OS) A T S G D G L Y E G L D W L S N Q L R
Lansoprazole
c
N W Y I Q A T C (C17H17N3O2S) A T S G D G L Y E G L D W L S N Q L R
Omeprazole
d
N W Y I Q A T C (C16H13F2N3O3S) A T S G D G L Y E G L D W L S N Q L R
Pantoprazole
Relative Abundance

Supplementary Fig. 6. Two-dimensional mass spectra of rabeprazole, lansoprazole, omeprazole, and pantoprazole. a~d, Q-Exactive tandem mass spectra results showed the modified peptide of ARF1, demonstrating that ARF1 was covalently modified by, rabeprazole (**a**), lansoprazole (**b**), omeprazole (**c**) and pantoprazole (**d**) at cysteine 159.

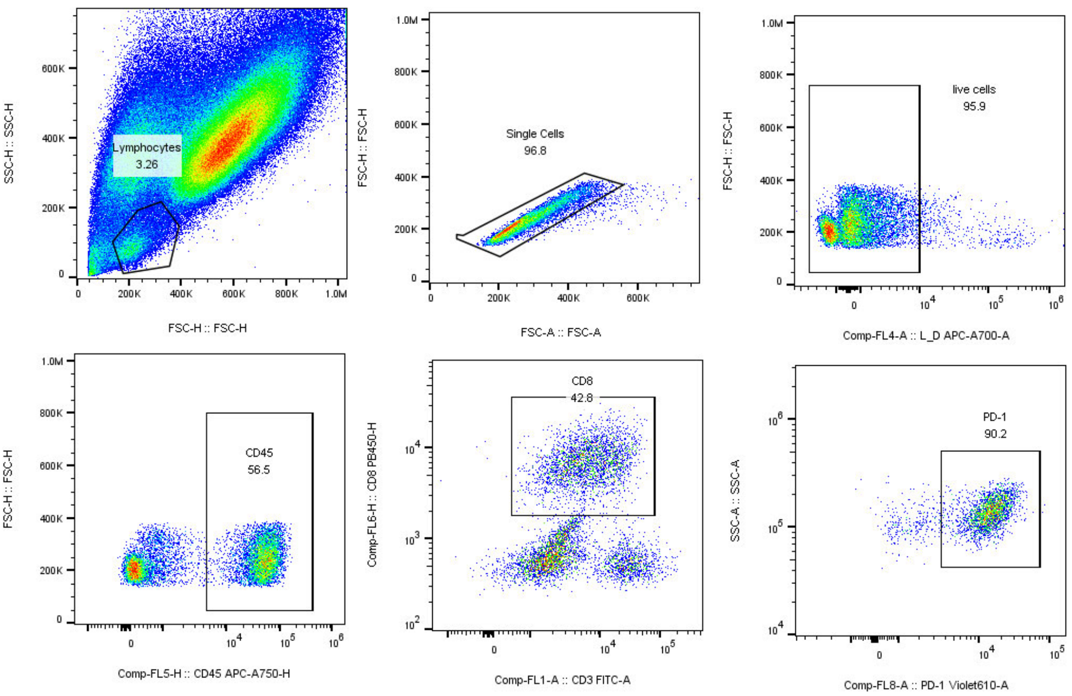

Supplementary Fig. 7. Flow cytometry gating strategies.

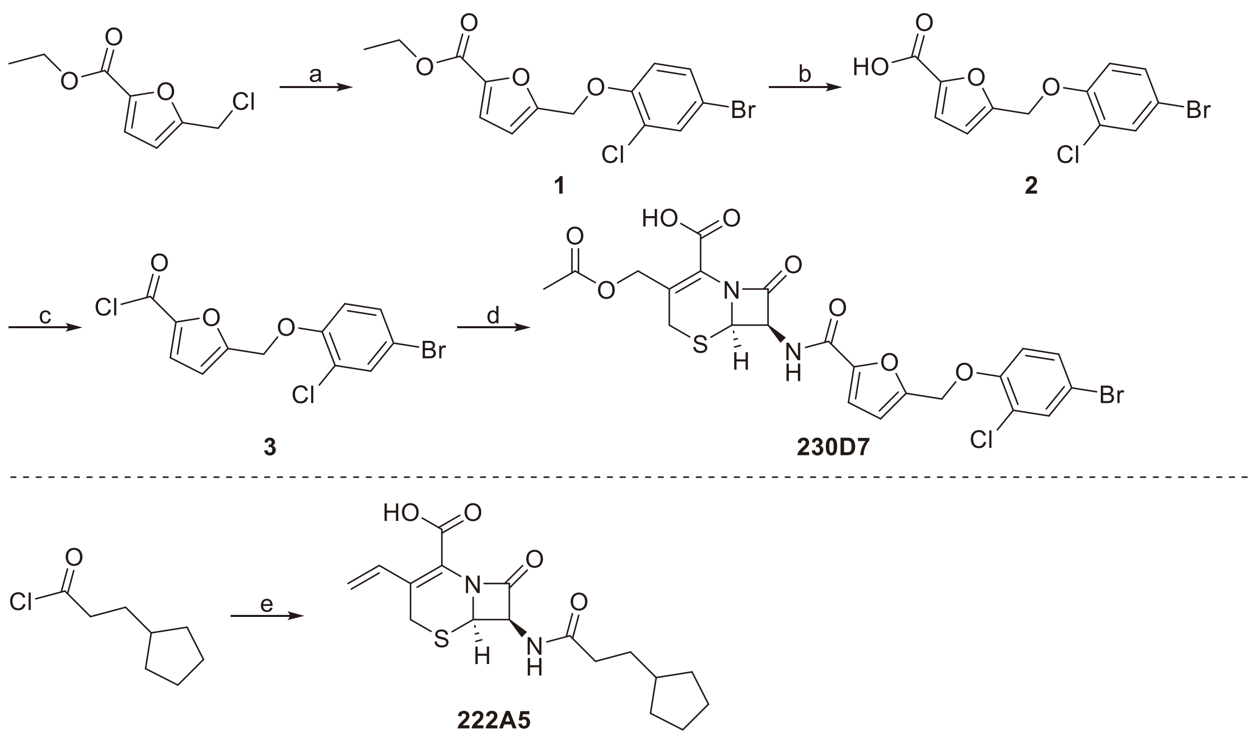

Supplementary Fig. 8. Synthesis of Compounds 230D7 and 222A5[a]. [a]Reagents and conditions: (a) 4-bromo-2-chlorophenol, K_2CO_3, DMF, 60 °C, 5 h; (b) NaOH, MeOH, H_2O, 50 °C, 2 h; (c) $SOCl_2$, reflux, 2 h; (d) (6*R*,7*R*)-3-(acetoxymethyl)-7-amino-8-oxo-5-thia-1-azabicyclo[4.2.0]oct-2-ene-2-carboxylic acid, $NaHCO_3$, acetone, H_2O, 0 °C−rt, 4 h; (e) (6*R*,7*R*)-7-amino-8-oxo-3-vinyl-5-thia-1-azabicyclo[4.2.0]oct-2-ene-2-carboxylic acid, $NaHCO_3$, acetone, H_2O, 0 °C−rt, 4 h.

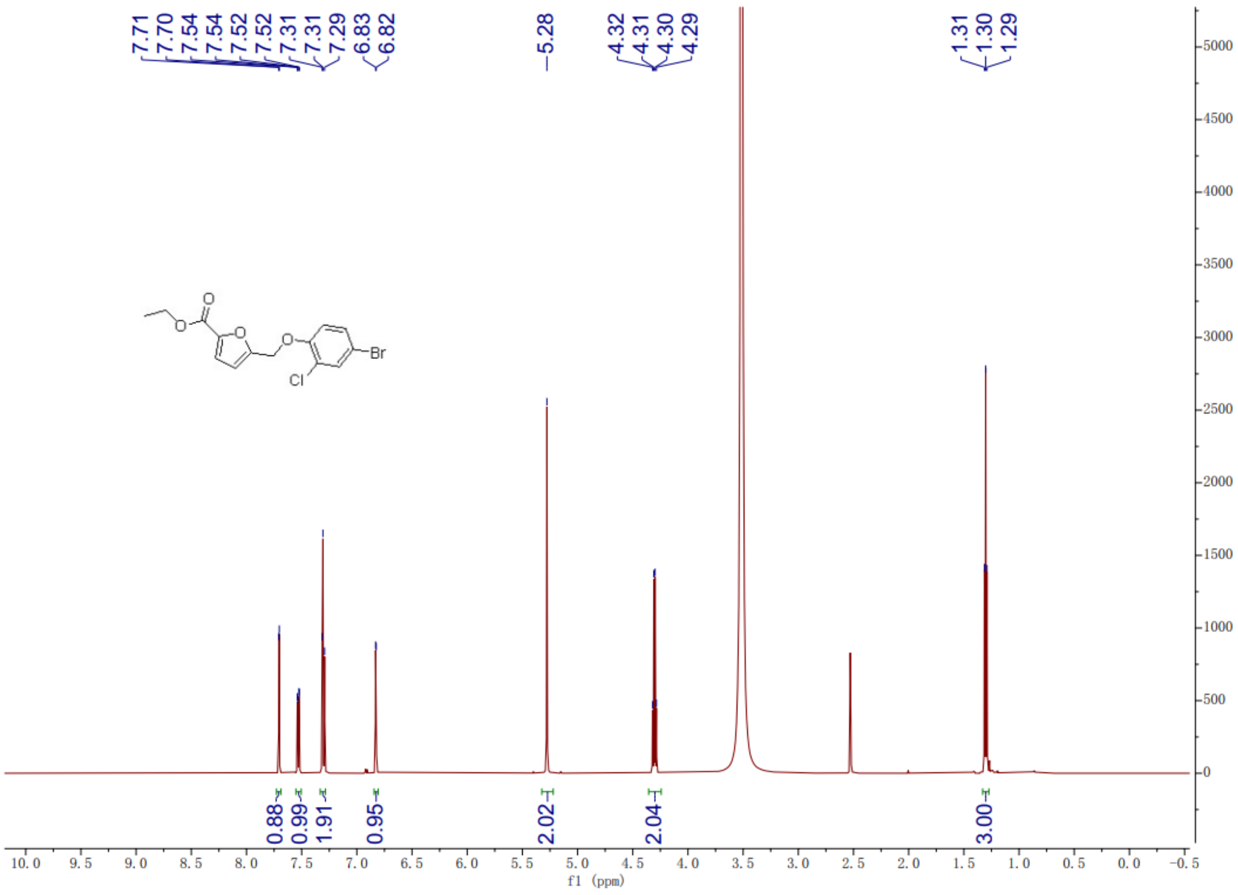

Supplementary Fig. 9. ^{1}H NMR of compound 1.

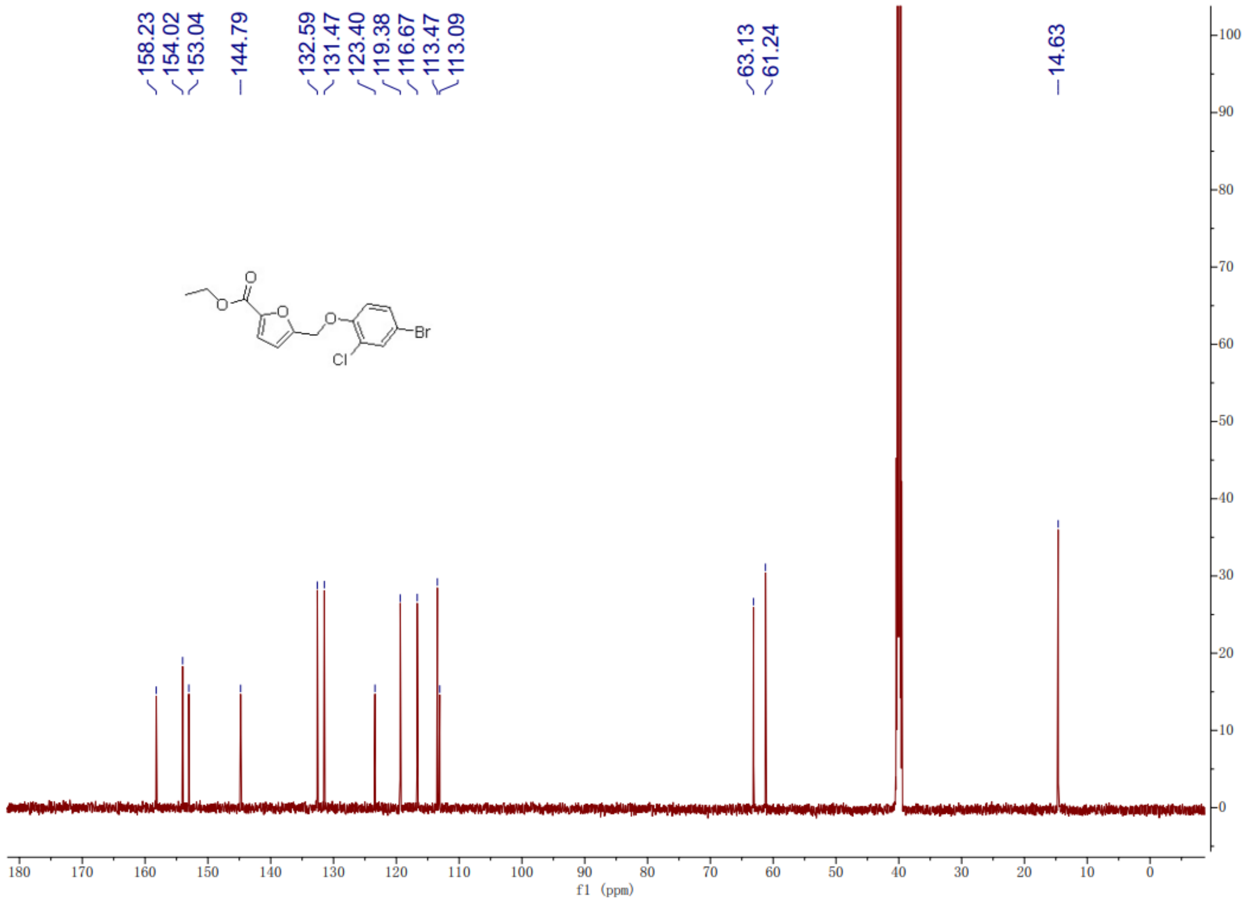

Supplementary Fig. 10. ^{13}C NMR of compound 1.

**Data Filename**	ESIH202302023.d	**Sample Name**	F1-MS-13294-Easter
**Sample ID**		**Position**	P1-A2
**Instrument Name**	Agilent G6520 Q-TOF	**Acq Method**	20160322_MS_ESIH_POS_1min.m
**Acquired Time**	3/20/2023 16:04:22	**IRM Calibration Status**	Success
**DA Method**	small molecular data analysis method.m	**Comment**	ESIH by fangsu

User Spectra

Fragmentor Voltage	Collision Energy	Ionization Mode
170	0	ESI

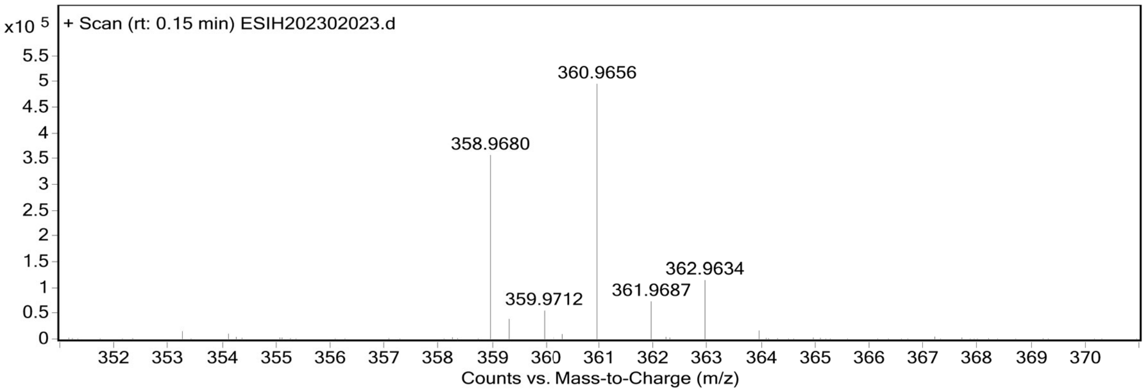

Formula Calculator Results

m/z	Calc m/z	Diff (mDa)	Diff (ppm)	Ion Formula	Ion
358.968	358.968	0.01	0.02	C14 H13 Br Cl O4	(M+H)+

Supplementary Fig. 11. HRMS of compound 1.

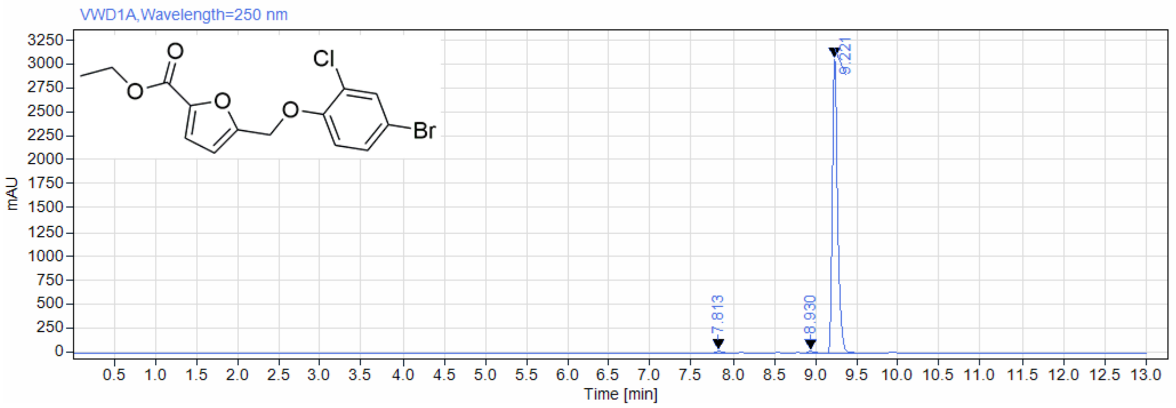

Signal: VWD1A,Wavelength=250 nm

RT [min]	Type	Width [min]	Area	Height	Area%	Name
7.813	BV	0.28	77.54	20.07	0.56	
8.930	VV	0.26	74.29	17.45	0.54	
9.221	VB	0.72	13604.55	3056.54	98.90	
		**Sum**	**13756.38**			

Supplementary Fig. 12. HPLC of compound 1.

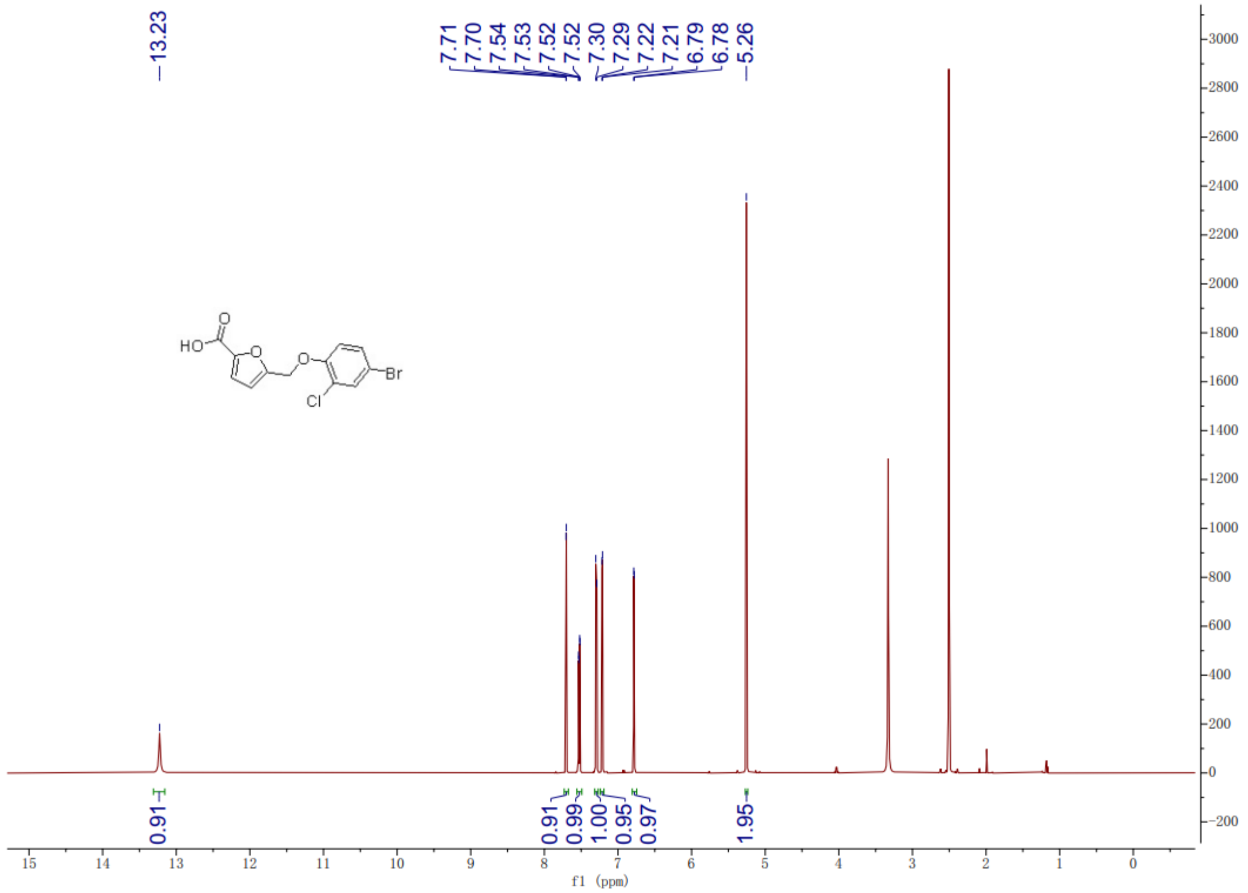

Supplementary Fig. 13. ¹H NMR of compound 2.

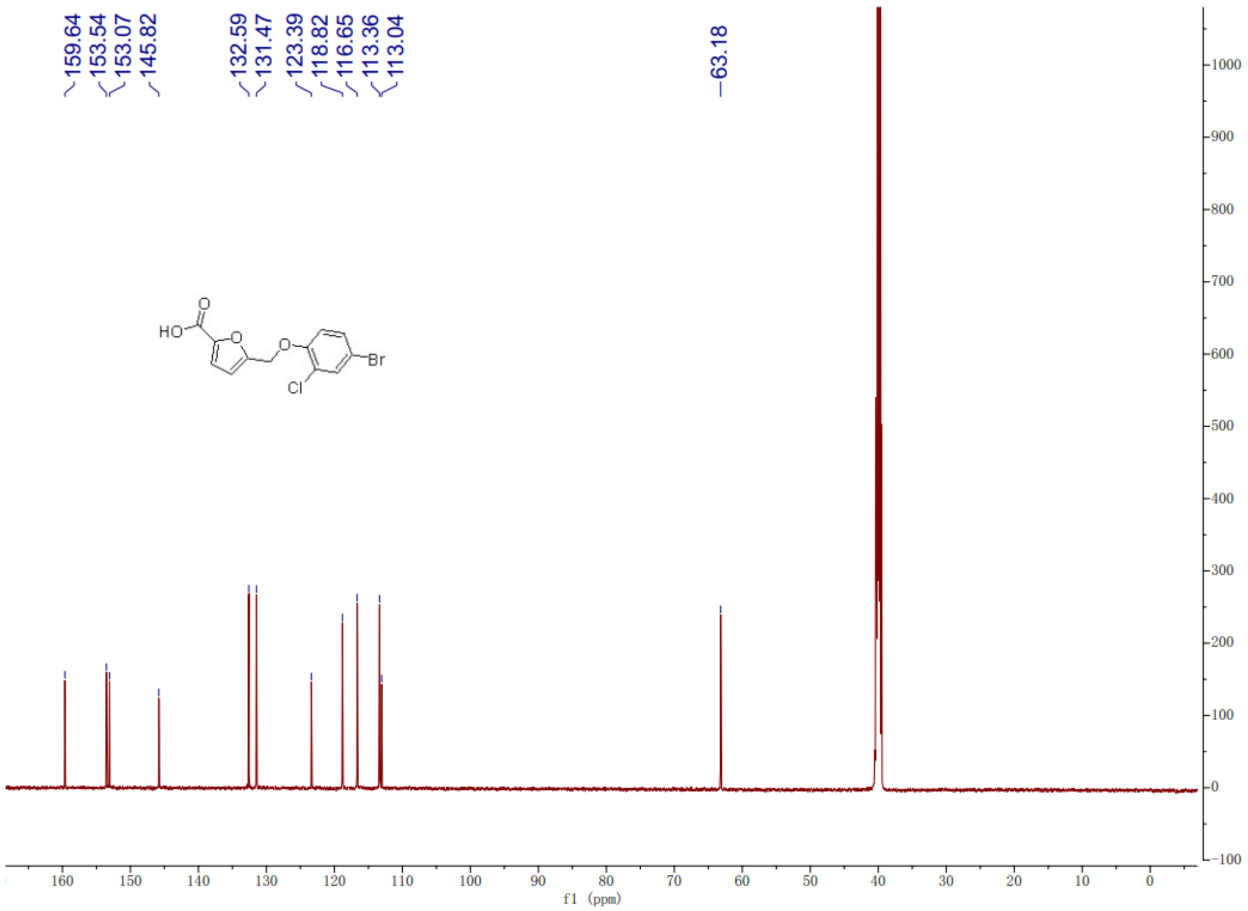

Supplementary Fig. 14. ^{13}C NMR of compound 2.

**Data Filename**	N_ESIH202302022.d	**Sample Name**	F1-MS-13294-Acid
**Sample ID**		**Position**	P1-A1
**Instrument Name**	Agilent G6520 Q-TOF	**Acq Method**	20160324_MS_ESIH_NEG_1min.m
**Acquired Time**	3/20/2023 16:07:46	**IRM Calibration Status**	Success
**DA Method**	small molecular data analysis method.m	**Comment**	ESIH by fangsu

User Spectra

Fragmentor Voltage	Collision Energy	Ionization Mode
170	0	ESI

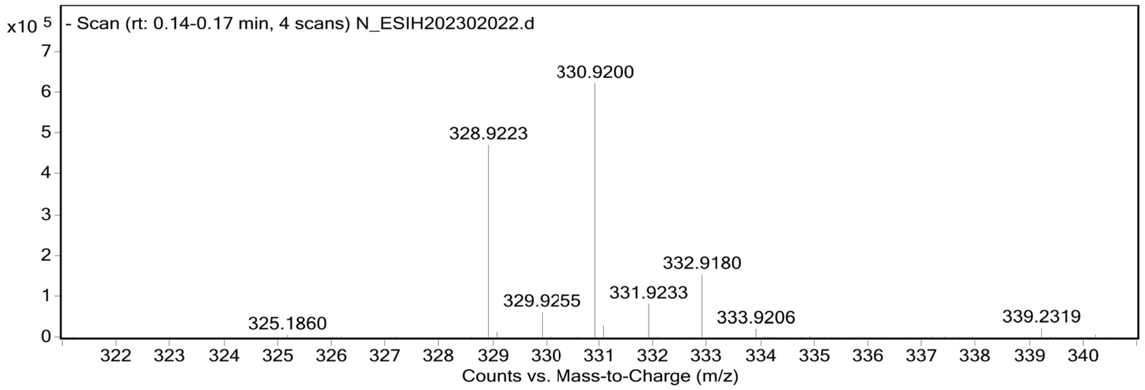

Formula Calculator Results

m/z	Calc m/z	Diff (mDa)	Diff (ppm)	Ion Formula	Ion
328.9223	328.9222	-0.08	-0.24	C12 H7 Br Cl O4	(M-H)-

Supplementary Fig. 15. HRMS of compound 2.

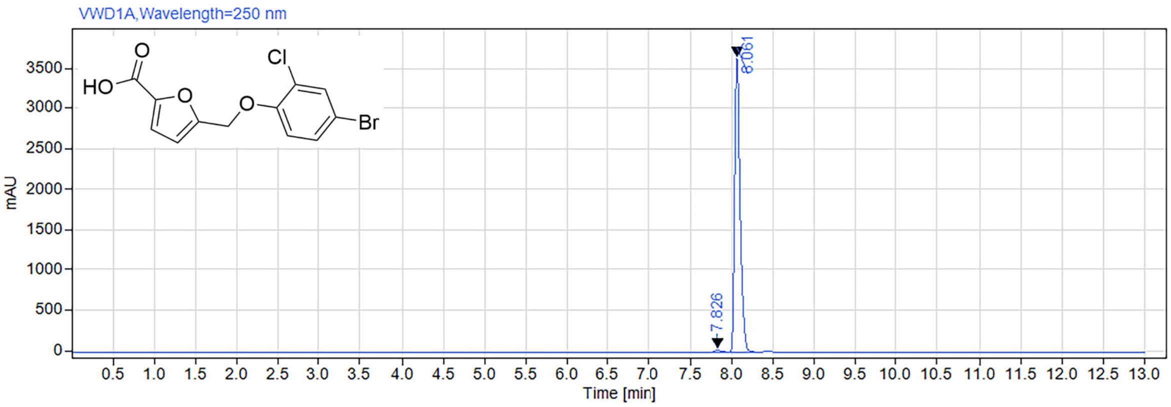

Signal: VWD1A,Wavelength=250 nm

RT [min]	Type	Width [min]	Area	Height	Area%	Name
7.826	BV	0.22	114.08	27.82	0.68	
8.061	VV	0.37	16751.46	3623.05	99.32	
		**Sum**	**16865.55**			

Supplementary Fig. 16. HPLC of compound 2.

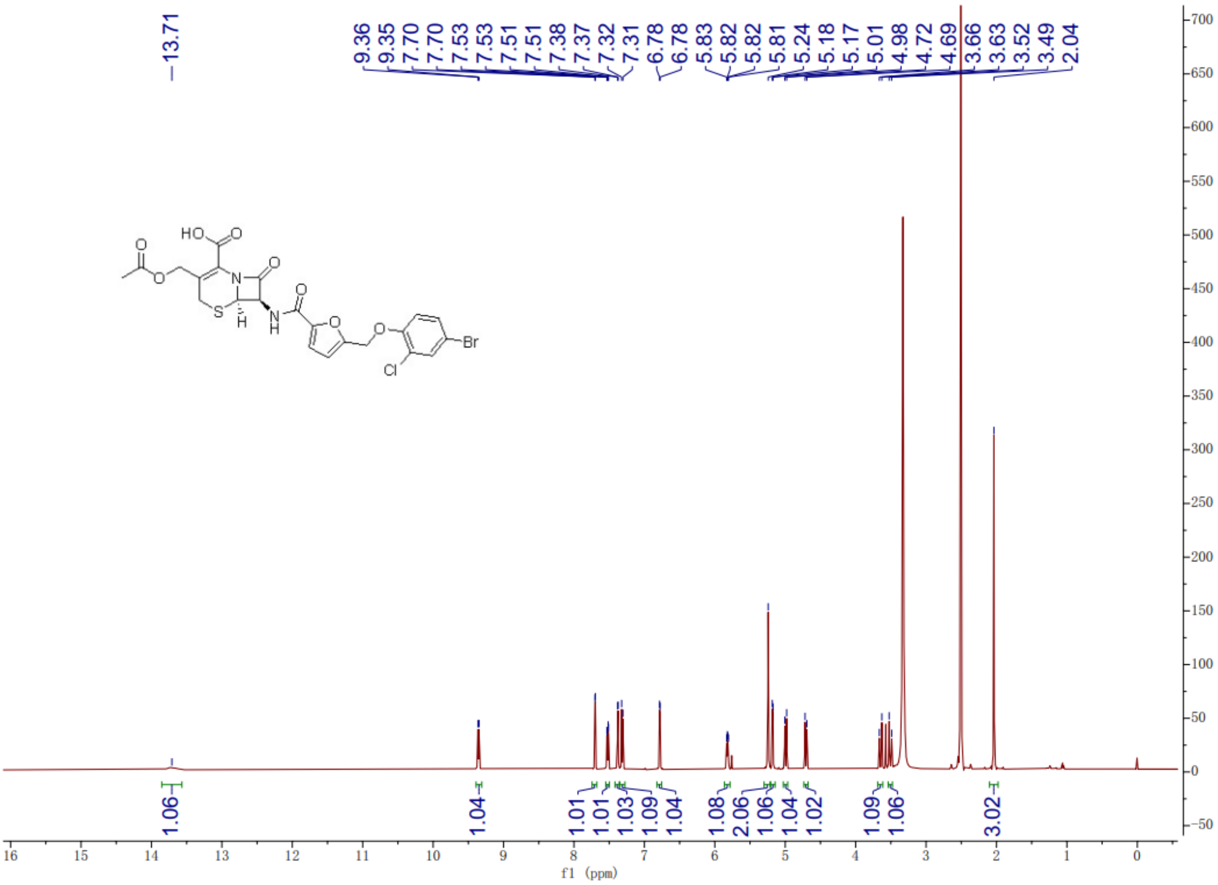

Supplementary Fig. 17. ^{1}H NMR of 230D7.

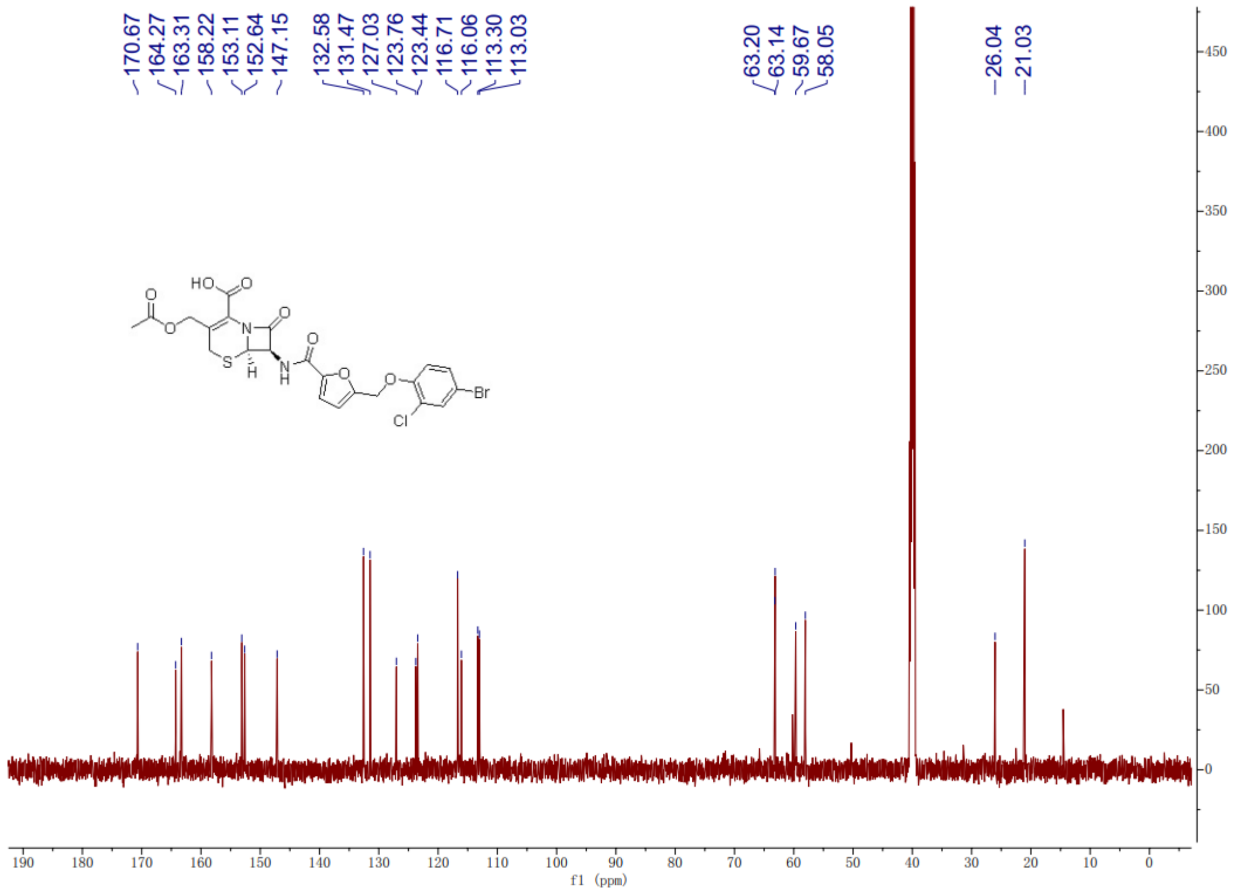

Supplementary Fig. 18. ^{13}C NMR of 230D7.

**Data Filename**	ESIH202204353.d	**Sample Name**	F1-F1-13294-230D7
**Sample ID**		**Position**	P1-A4
**Instrument Name**	Agilent G6520 Q-TOF	**Acq Method**	20160322_MS_ESIH_POS_1min.m
**Acquired Time**	11/2/2022 15:59:50	**IRM Calibration Status**	Success
**DA Method**	small molecular data analysis method.m	**Comment**	ESIH by fangsu

User Spectra

Fragmentor Voltage	Collision Energy	Ionization Mode
170	0	ESI

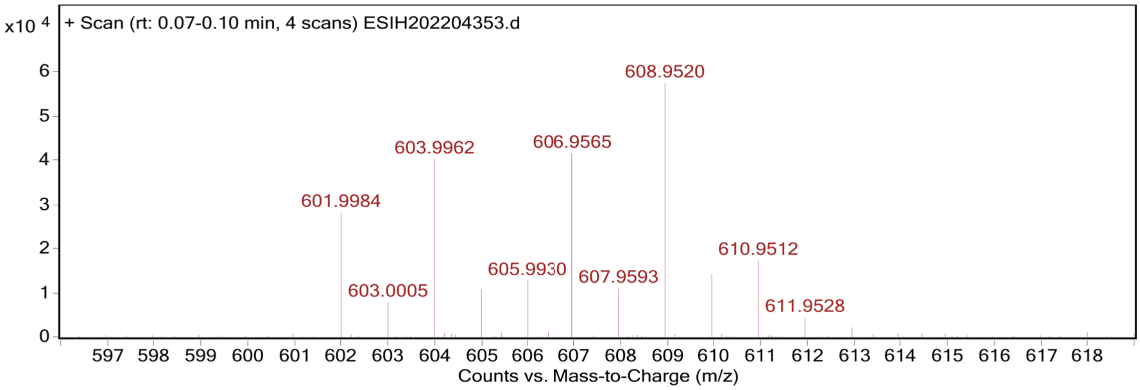

Formula Calculator Results

m/z	Calc m/z	Diff (mDa)	Diff (ppm)	Ion Formula	Ion
606.9565	606.9548	-1.72	-2.84	C22 H18 Br Cl N2 Na O8 S	(M+Na)+
601.9984	601.9994	0.98	1.62	C22 H22 Br Cl N3 O8 S	(M+NH4)+

Supplementary Fig. 19. HRMS of compound 230D7.

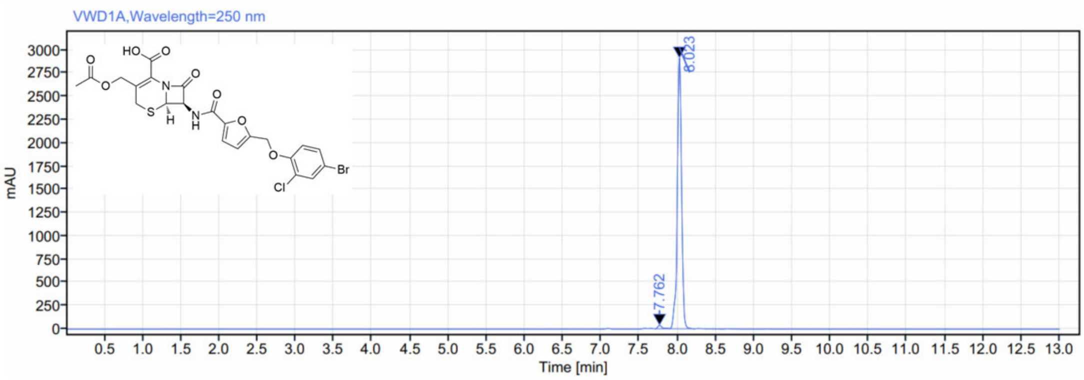

Signal: VWD1A,Wavelength=250 nm

RT [min]	Type	Width [min]	Area	Height	Area%	Name
7.762	BV	0.20	159.92	37.63	1.40	
8.023	VB	0.32	11239.88	2910.64	98.60	
		**Sum**	**11399.80**			

Supplementary Fig. 20. HPLC of 230D7.

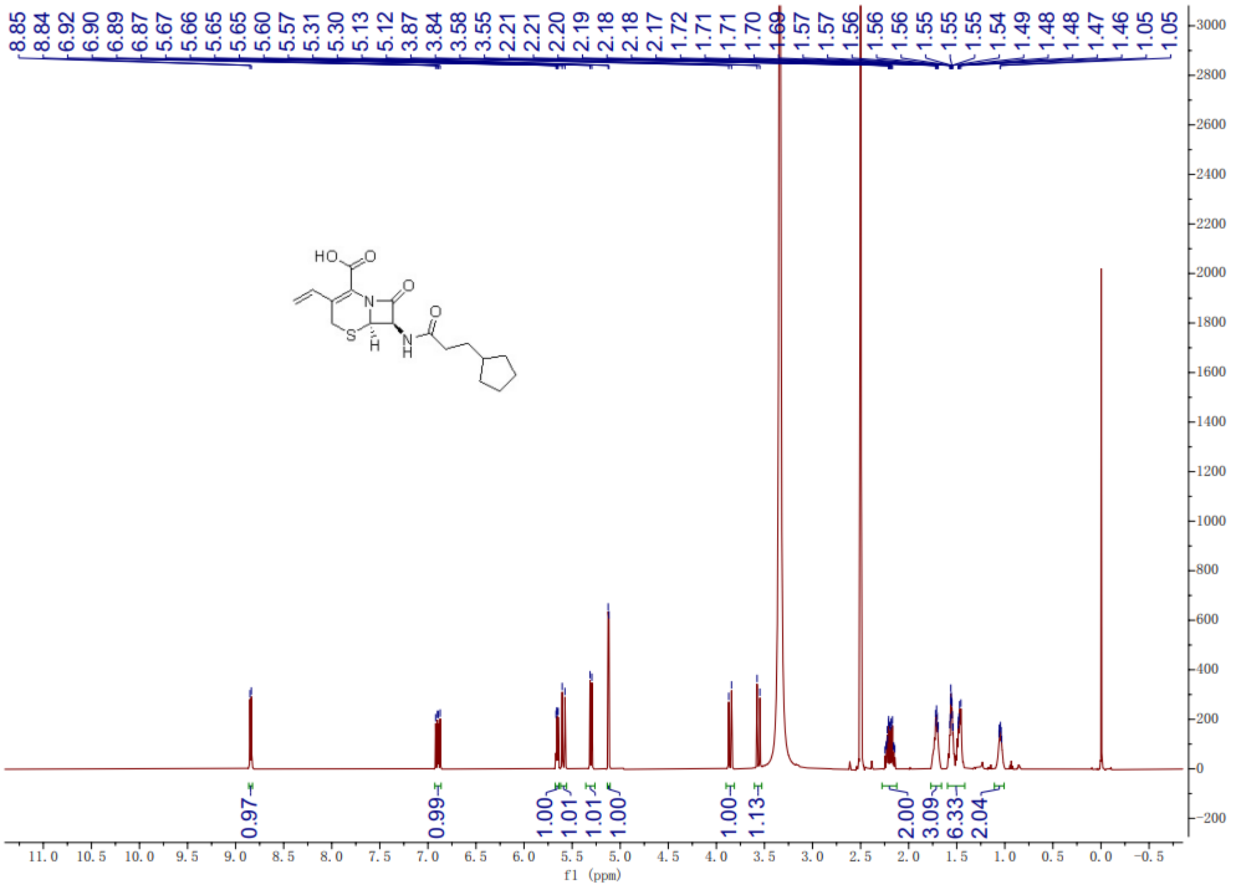

Supplementary Fig. 21. ^{1}H NMR of 222A5.

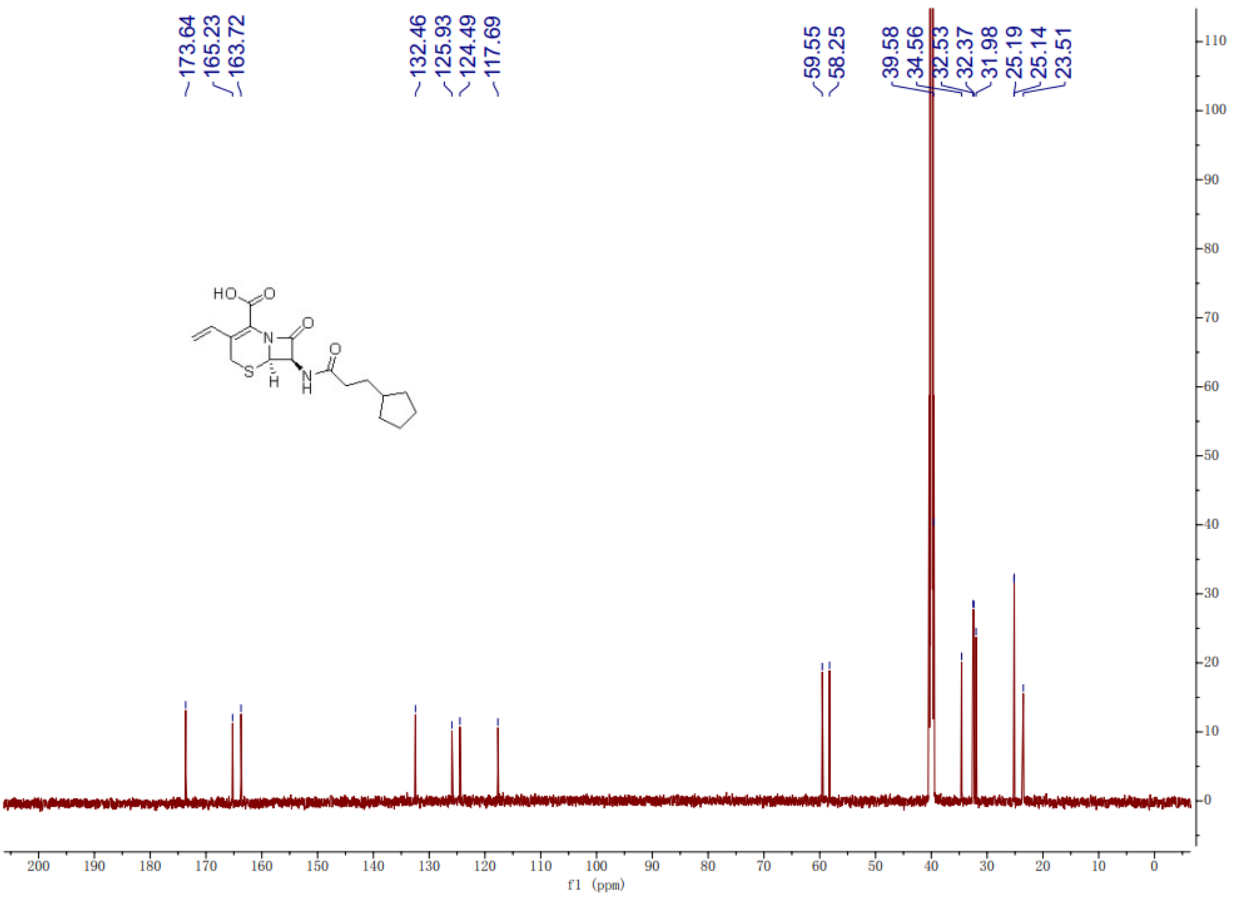

Supplementary Fig. 22. ^{13}C NMR of 222A5.

**Data Filename**	ESIH202302087.d	**Sample Name**	F1-F1-MS-222A5
**Sample ID**		**Position**	P1-A4
**Instrument Name**	Agilent 6520 Q-TOF	**Acq Method**	20160322_MS_ESIH_POS_1min.m
**Acquired Time**	3/23/2023 14:45:53	**IRM Calibration Status**	Success
**DA Method**	small molecular data analysis method.m	**Comment**	ESIH by fangsu

User Spectra

Fragmentor Voltage	Collision Energy	Ionization Mode
170	0	ESI

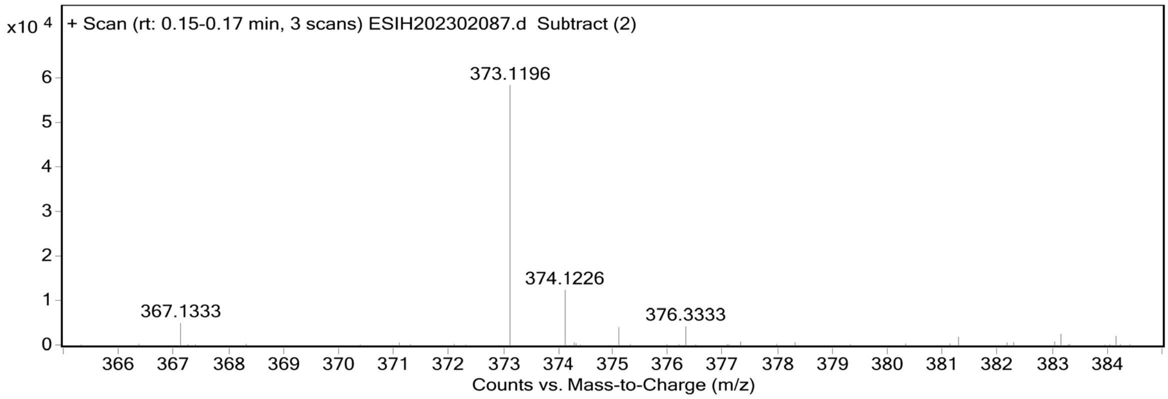

Formula Calculator Results

m/z	Calc m/z	Diff (mDa)	Diff (ppm)	Ion Formula	Ion
373.1196	373.1192	-0.37	-0.98	C17 H22 N2 Na O4 S	(M+Na)+

Supplementary Fig. 23. HRMS of compound 222A5.

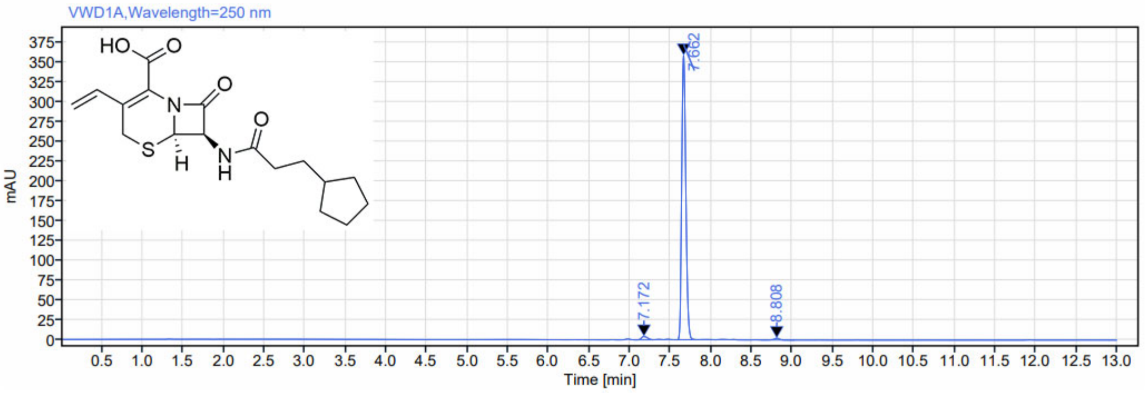

Signal: VWD1A,Wavelength=250 nm

RT [min]	Type	Width [min]	Area	Height	Area%	Name
7.172	BV	0.22	21.73	4.17	1.72	
7.662	BB	0.28	1233.89	358.07	97.48	
8.808	VB	0.41	10.19	2.28	0.81	
		**Sum**	**1265.81**			

Supplementary Fig. 24. HPLC of 222A5.

Supplementary Tab 1. **External performance on a large external set.**

	**TransformerCPI 2.0**	**TransformerCPI**	**GraphDTA**	**GCN**	**MolTrans**	**CPI-GNN**
AUC	0.670	0.638	0.646	0.641	0.630	0.519
PRC	0.395	0.360	0.346	0.341	0.340	0.311

Supplementary Tab 2. External performance on a time-split set.

	**TransformerCPI 2.0**	**TransformerCPI**	**GraphDTA**	**GCN**	**MolTrans**	**CPI-GNN**
AUC	0.639	0.605	0.609	0.605	0.599	0.525
PRC	0.637	0.589	0.590	0.592	0.595	0.517

Supplementary Tab 3. EF values of different tools on DUD-E set.

	TransformerCPI2.0	GOLD	AutoDock Vina
**EF0.5%**	11.028	14.659	9.516
**EF1.0%**	8.488	11.983	7.995
**EF5.0%**	4.286	5.872	4.382

Supplementary Tab 4. EF values of different tools on Dekois 2.0 set.

	TransformerCPI2.0	GOLD	AutoDock Vina
**EF0.5%**	6.460	5.750	5.464
**EF1.0%**	5.490	5.364	4.513
**EF5.0%**	3.319	3.348	2.824

Supplementary Tab 5. Performance on the whole substitution dataset.

Accuracy	TransformerCPI2.0	TransformerCPI	GraphDTA	GCN	Blind Guess
**overall**	0.567±0.006	0.504±0.010	0.506±0.007	0.507±0.010	0.5

Supplementary Tab 6. Performance on the subset dataset, where the corresponding biological activities increase or decrease by at least three orders of magnitude.

Accuracy	TransformerCPI2.0	TransformerCPI	GraphDTA	GCN	Blind Guess
**overall**	0.641±0.008	0.566±0.008	0.575±0.011	0.517±0.009	0.5

Supplementary Tab 7. SPOP screen compound list.

ID	ChemDiv_ID	SMILES	SCORE
221A3	Y020-9678	O=C2N(C=CC=3N=CC=1C(=O)N(C=CC=1C2=3)C4=CC=C(C=C4)OC)N5C=NN=C5	0.717187
221A4	Y020-9740	[Cl]/C2=N/C=1CCCCC=1N2	0.911182
221A5	Y040-0758	O=C4OC2=CC(C)=CC(OCC(=O)N[C@]1([H])CCC[C@@](C)([H])[C@]1(C)[H])=C2C=3CCCCC=34	0.795049
221A6	Y040-6856	O=C(O)[C@@]1([H])N[C@]([H])([S]C1)C=2/C=C(/O)C(=CC=2)OC	0.677123
221A7	Y042-2850	O=C3C(O)=C(/C1=C/N(C)N=C1)C2=CC=CC=C2N3C	0.689197
221A8	Y042-7195	O=C5N4C1=CC=CC=C1C(=O)N(CC(=O)N/C3=C/C=2C=CC(=CC=2N=C3)OC)[C@]4([H])C=6C=CC=CC5=6	0.742336
221A9	Y042-7748	O=C2C=C(N=C1[S]C=NN12)C[S]CCN	0.773825
221A10	Y043-0432	O=C5N(C)[C@]([H])(C=2C=C1OCOC1=CC=2)[C@@]([H])(C(=O)NC=4C=C3NC=CC3=CC=4)C6=CC=CC=C56	0.76356
221B3	Y203-9186	O=C(N/C1=N/C(=C[S]1)C2=CC=CC=3C=CC=CC2=3)[C@]4([H])CCCC[C@@]4([H])C(=O)O	0.911547
221B4	Y203-9240	O=C(N/C1=C/C=C/C=C1/CC)[C@]3([H])[C@@]2([H])C=C[C@]([H])(C2)[C@]3([H])C(=O)O	0.75825
221B5	Y203-9473	O=C(NC=1C=C(C=CC=1)CC)[C@]2([H])CC=CC[C@@]2([H])C(=O)O	0.788327
221B6	Y205-4786	O=C(N[C@@](C)([H])[C@]1([H])OCCC1)C/C2=C(\[Cl])C=C/C=C2/[F]	0.665194
221B7	Y205-4803	O=[S](=O)(N1CCC=2C=CC=CC1=2)C=5C=CC(NC(=O)[C@]3([H])[C@]([H])(C(=O)O)[C@]4([H])O[C@@]3([H])CC4)=CC=5	0.73789
221B8	Y205-8024	O=C(N[C@]1([H])CCCC1)C2=C([S]C(C)=C2C)NC(=O)[C@]3([H])[C@@]([H])(C(=O)O)[C@]4([H])C=C[C@]3([H])CC4	0.66742
221B9	Y205-9539	O=C(NC=2C=C1CCCC1=CC=2)[C@]3([H])[C@]([H])(C(=O)O)[C@]4([H])O[C@@]3([H])C=C4	0.718496
221B10	Y205-9761	O=C(N/C1=C/C=C(/[F])C=C1[F])[C@@]2([H])[C@@]([H])(C(=O)O)[C@@]3([H])O[C@]2([H])CC3	0.754913
221C3	Y206-4045	O=C(NC=1/C=C(/OC)C=CC=1)[C@@]2([H])[C@@]([H])(C(=O)O)[C@@]3([H])C[C@]2([H])CC3	0.69853
221C4	Y206-4218	O=C(N2C=1C=CC=CC=1C[C@]2(C)[H])[C@@]3([H])[C@@]([H])(C(=O)O)[C@@]4([H])C[C@]3([H])CC4	0.843582
221C5	Y500-7017	[F]C([F])([F])C1=CC(=NC2=C1C(=NN2C)C3([H])CC3)C=4C=NN(CC)C=4C	0.752009
221C6	Y501-9359	N#CC=1C=NN2C=1N[C@]([H])(C[C@@]2([H])C([F])([F])[F])C=3C=CC([F])=CC=3	0.677206
221C7	Y502-3210	O=C1N2C(C(O)=O)=C(CSC3=NN=C(C)S3)CSC2C1NC(C4=CC=C(COC5=C(Cl)C=CC=C5)O4)=O	0.715588
221C8	Y502-9376	O=C1N(C(=O)[C@@]2([H])CCCC[C@@]12[H])[C@@](C)([H])C3=CC(=CC=C3)N4C=CC=C4	0.780379
221C9	Y503-2847	O=C(NN)[C@](C)([H])N1N=C(/C=C1/[C@]2([H])CC2)C([F])([F])[F]	0.729244
221C10	Y600-3985	O=C1N(NC2=CC(=O)N(C(C)=C12)C3([H])CCCCC3)C5=NC=4C=CC=CC=4[S]5	0.714081
221D3	ZE09-1328	O=C(C=2[S]C1=C/C=C(/NC(=O)[C@@]([Cl])(C)[H])C=C1C=2OC)N3CCCCC3	0.789824
221D4	Y043-4548	O=C1N[C@@]2([H])[C@@]([H])(N1)C[S][C@@]2([H])CCCCC(=O)NCC/C3=C/NC4=C/C=C(/[Cl])C=C34	0.818989
221D5	Y205-0195	N#C/C1=C(/[S]C=2CCCCCCCCCCC1=2)NC(=O)[C@]3([H])CCCC[C@@]3([H])C(=O)O	0.68645
221D6	Y600-4705	O=C1C(C)=C(N/C=C1/C)C[Cl]	0.733618

231 A3	1185-0186	[Cl]/C1=C/C=CC([Cl])=C1N/C2=N/C(=C([S]2)C=3C=CC=CC=3)C=4C=CC([S]C)=CC=4	0.827448
231 A4	3091-4746	O=C3N(C=1/C=C(/[F])C(C)=CC=1)[C@]([H])(C2=CC(=C/C=C2/OC)OC)[C@@]3([H])N5C(=O)C=4C=C(C=CC=4C5=O)[N+]([O-])=O	0.698414
231 A5	3238-0180	O=C3N(C)C=2/N=C(/NCCCOC)N(C/C1=C/C=C/C=C1/[F])C=2C(=O)N3C	0.784697
231 A6	3260-0051	N=C3N(C[C@](O)([H])C=1[S]C=CC=1)C2=CC=CC=C2N3CCN4CCCCC4	0.759465
231 A7	4593-5763	O=C5N(C=2[S]C=1CCCCC=1C=2C(=O)OCC)C(=O)[C@@]6([H])ON(C=3C=CC=CC=3C)[C@@]([H])(C4=C/C=C(/C)C=C4)[C@]56[H]	0.978679
231 A8	5055-3945	N#C/C1=C(/[S]C=2CCCCC1=2)N6C(=O)[C@@]5([H])ON(/C3=C/C=C(/[Cl])C=C3)[C@@]([H])(C=4OC=CC=4)[C@]5([H])C6=O	0.979211
231 A9	5055-4009	O=C4N(C(=O)[C@]3([H])ON(C=1C=CC=CC=1C)[C@]([H])(C=2[S]C=CC=2)[C@@]34[H])C5=C/C=C(/[Cl])C=C5	0.893642
231 A10	5282-0816	N=C3N(C=2N=C1C=CC(C)=CN1C(=O)C=2/C=C3/C(=O)N[C@@]4([H])CCCC4)[C@@]5([H])CCCC5	0.679165
231 B3	5339-0200	O=C4N(C)C=3N=CN(C/C1=C/C=CC2=CC=CC=C12)C=3C(=O)N4C	0.70898
231 B4	5782-4753	O=C1N(C(=O)[C@]2([H])[C@@]1([H])[C@]3([H])C[C@@]2([H])CC3)[C@]([H])(C/C4=C/C=CC=C4)C(=O)N/C5=C/C([Cl])=C/C=C5/C	0.840434
231 B5	5847-0271	O=C1C(=COC=2C1=C/C=C(/O)C=2CN4CCN(CC=3C=CC=CC=3)CC4)C5=CC=C/C=C5/[Cl]	0.732585
231 B6	6056-0816	O=C(/C2=C/N(CC(=O)NC[C@@]1([H])OCCC1)C3=C2C=C/C=C3/CC)C([F])([F])[F]	0.683887
231 B7	7238-1536	O=C(N/C1=C(\C)C=C/C=C1/CC)[C@]3([H])[S]C2=NN=C(N2N[C@@]3([H])C4=CC=CC=C4)C5=CC=CC=C5	0.747739
231 B8	8001-2890	O=C(C=2[S]C=1N=C(C=CC=1C=2N)C=3[S]C=CC=3)N5CCC=4C=CC=CC=4[C@]5([H])CNC(=O)[C@@]6([H])CCCCC6	0.697008
231 B9	8006-9338	O=C1N(C(=O)[C@]2([H])[C@@]1(C)[C@@]3([H])C=C[C@]2([H])C3)C=4C=CC(=CC=4)C(=O)OCC	0.813252
231 B10	8006-9693	O=C1N(C(=O)[C@@]2([H])[C@@]1([H])[C@]([H])(CC[C@]2([H])C3=CC=CC=C3)C4=CC=CC=C4)C=5C=CC(=CC=5)[N+]([O-])=O	0.741062
231 C3	8010-8365	O=C(C)C1=C(C[C@](O)(C)[C@@]([H])(C(=O)C)[C@]1([H])C=2/C=C(/OC)C(=CC=2)OC)N/C3=C/C=C(/[Br])C=C3	0.679074
231 C4	8011-5826	O=C1N(C(=O)[C@@]3([H])[C@@]1([H])[C@]4([H])C=2C=CC=CC=2[C@@]3([H])C=5C=CC=CC4=5)[C@@]([H])(CC[S]C)C(=O)NC=6/C=C(/[Cl])C([Cl])=CC=6	0.74474
231 C5	8012-5299	[O-][N+](=O)C2=C1C=CC(=NC1=C/C=C2/C)C4=CC=3/C=C(/C)C=CC=3N=C4	0.726103
231 C6	8012-7811	O=C2N(C=1C=CC([Br])=CC=1[C@]2([H])[C@]4([H])[S]C=3C=CC=CC=3NC4=O)C(=O)C	0.719762
231 C7	8013-0260	O=C(N1[C@]([H])(C[S][C@]1([H])C2=C/C=C(/C)C=C2)C(=O)O)C3=CC([Cl])=CC=C3	0.865409
231 C8	8013-1552	O=C4[S]C2=C([S][C@]([H])(C(=O)O)[C@@]3([H])C(=O)OC=1C=CC=CC=1[C@]23[H])N4C/C5=C/C=CC=C5	0.830558
231 C9	8013-5880	O=C3N(CC/C1=C(\C)NC=2C=CC(=CC1=2)OC)C(=O)[C@@]4([H])CCCC[C@]34[H]	0.853814
231 C10	8014-8857	O=C1N(C(=O)[C@@]2([H])[C@]1([H])[C@@]([H])(N[C@]2(CC)C(=O)O)C3=CC=C/C=C3/O)C4=CC([Cl])=CC=C4	0.905549
231 D3	8015-3422	CO/C4=C/C=3CC[C@]2([H])[S]C=1C=CC=CC=1N=C2C=3C=C4	0.82399
231 D4	8015-7350	O=C1N(C(=O)[C@@]2([H])[C@]1([H])[C@@]3(N[C@@]2([H])CC(C)(C)[H])C(=O)N(CC(=O)OCC)C4=CC=CC=C34)C=5C=CC=C6C=CC=CC=56	0.953058
231 D5	8016-0378	O=C1N(C(=O)[C@@]2([H])[C@@]1([H])[C@@]4(N[C@@]2(C)[H])C(=O)N(C/C3=C/C=C(/[F])C=C3)C=5C=CC=CC4=5)C6=CC=CC=C6	0.909611
231 D6	8017-1330	O=C1C(=O)[C@]3(C)CC[C@@]1(C(=O)NC=2/C=C(/[F])C=CC=2[F])C3(C)C	0.896598
231 D7	8017-2278	O/C1=C/C=C(C=C1)C4=NN2C(=N/N=C2/C3=C/C=NC=C3)C=5C=CC=CC4=5	0.69382
231 D8	8017-3040	O=C3O[C@@]([H])(C1=CC=CC=C1)[C@]([H])(C=2C=CC=CC=2)[C@@]3([H])NC=4C=CC([I])=CC=4C(=O)O	0.719256

231 D9	8017-6609	O=C1N(C(=O)[C@]2([H])[C@@]1([H])[C@]4([H])C[C@@]2([H])[C@@]5([H])O/N=C(/C3=C/C=C(/C=C3/OC)OC)[C@@]45[H])C=7C=C6C=CC=CC6=CC=7	0.755072
231 D10	8018-6686	[O-][N+](=O)C=1C=CC(=CC=1)[C@]4([H])O[C@]2([H])CCCC[C@@]2([H])C=3[S]C=CC=34	0.687403
231 E3	8018-7502	O=C4OC(C)(C)[C@]3(C)C2=CC=1C=CC=CC=1N2CCN34	0.888736
231 E4	8019-7720	O=C1CC[C@@]([H])(C(=O)O)[C@@]([H])(N1CC=2C=CC=CC=2)C3=CC=C(C=C3)OC	0.679759
231 E5	8020-2909	[Cl]/C1=C/C=C/C=C1/CN3/C=C(/CNC/C2=C/C=CC=C2)C4=CC=CC=C34	0.818032
231 E6	C082-0552	O=C2N(CCC(=O)N/C1=C/C([F])=C(C)C=C1)C(=O)[C@@]3([H])[C@]2([H])[C@@]4([H])C=C[C@@]3([H])CC4	0.702275
231 E7	C167-0196	O=C(NCCN1CCCC2=CC=CC=C12)C3=CC(=NC4=CC=CC=C34)C=5[S]C=CC=5	0.786434
231 E8	C202-1469	O=C5N(C(=O)[C@]4([H])[C@@]1(CC(=O)N(C1=O)C2=C/C=C(/C)C=C2)N(CCCCC)[C@@]([H])(C=3C=CC(=CC=3OC)OC)[C@]45[H])C=6C=CC(C)=CC=6	0.986464
231 E9	C289-0208	O=C4N2CCC=1/C=C(/OCC)C(=CC=1[C@]2([H])[C@]([H])(C3=CC=CC=C34)C(=O)NCCCN5CCN(CC5)C6=CC=C/C=C6/[F])OCC	0.777312
231 E10	C362-0183	O=C3C2=CC1=C(C=C/C=C1/[Cl])N(C2=NC(=O)N3[H])C=5C=C4C=CC=CC4=CC=5	0.679811
231 F3	C656-0366	O=C2C=1N=NN(C=1N=CN2CC=3/C=C(/C)C=CC=3C)C=4C=CC(C)=CC=4	0.704669
231 F4	C801-0098	O=C2C=1C=NN(C=1N=CN2CC=3/C=C(/[Cl])C=CC=3)C=4C=CC=CC=4	0.75948
231 F5	C890-0681	O=C1C=3C(C=NN1/C2=C/C=CC=C2)=C(C)N(C=3C)[C@]([H])(CC)C(=O)N[C@]5([H])CCN(C/C4=C/C=CC=C4)CC5	0.667658
231 F6	CM4439-2906	O=C2N1CCN(CC1=NN2CCC)C/C3=C/N/N=C3/C4=C/C(C)=C(C)C=C4	0.682379
231 F7	D090-0038	[Cl]C=4C=C3C=2CCC1=CNN=C1C=2NC3=CC=4	0.816077
231 F8	D153-0157	O=C(C)C1=NN(/N=C1/NC=2C=C(C=CC=2)N3N=NN=C3)C4=C/C=C(/OC([F])([F])[F])C=C4	0.706973
231 F9	D225-0021	O=C2C=1NN=C(C=1[C@@]([H])(N2C)C=3C=NC=CC=3)C4=CC=C(C=C4)OC	0.705185
231 F10	D505-0628	O=[S](=O)(NCC=1C=CC([F])=CC=1)C2=CC(=C/C=C2/CC)C3=NN(C)C(=O)C=4CCCCC3=4	0.709343
231 G3	E155-1258	O=[S](=O)(C)NC[C@@]([H])(N1CCN(CC1)C=2C=CC(=CC=2)OC)C=3C=NC=CC=3	0.668649
231 G4	E209-0953	O=C2N(C=CN(C/C1=C/C(=CC=C1)C([F])([F])[F])C2=O)C3=CC(C)=C(C)C=C3	0.703854
231 G5	E612-0534	O=C1N(CC2=CC=CC=C12)[C@]([H])(C/C3=C/C=CC=C3)C(=O)NCC/C4=C/NC=5C=CC=CC4=5	0.810952
231 G6	E634-1423	CN1C(=CC=2OC=CC1=2)C5=N/N=C(/[S]CC=3N=C(OC=3C)C=4[S]C=CC=4)N5C=6C=CC=CC=6C	0.67575
231 G7	E959-0971	O=C3/C(=C(/N[C@]1([H])CCCC=2C=CC=CC1=2)C3=O)C=5C=C4CCCCC4=CC=5	0.926352
231 G8	F321-0661	O=C(NC=1C=C(C=CC=1)C=2N=C(ON=2)[C@@]3([H])CCCC3)[C@]4([H])CCCCC4	0.739761

Supplementary Tab 8. Ranking result of TransformerCPI2.0 and other tools screening the same compound library against SPOP.

Method	221C7 ranking
**TransformerCPI2.0**	34,495/1386372, Top 2.5%
**TransformerCPI**	1,367,035/1386372, Top 98.6%
**GraphDTA**	231,129/1386372, Top 16.7%
**GCN**	385,186/1386372, Top 27.8%
**Docking (Glide)**	229,874/1386372, Top 16.6%

Supplementary Tab 9. Tanimoto similarity between hits and known SPOP inhibitors calculated by ECFP fingerprints.

	**6b**
**221C7**	0.18
**221C10**	0.15
**231A10**	0.30
**231D8**	0.16

Supplementary Tab 10. RNF130 screen compound list.

ID	Chemspace_ID	SMILES	SCORE
iRNF130-1	CSC000581113	CS(=O)(=O)C=1C=CC(Cl)=C(NC(=O)C2CC2)C1	0.956755
iRNF130-2	CSC026929245	CN1N=NN=C1SCC=2C=C(Cl)C=3OCCOC3C2	0.910054
iRNF130-3	CSC016396878	NC(=O)CSC1=NN=C(NC2CC2)S1	0.928877
iRNF130-4	CSC133087246	BrC=1C=CC=C(C1)[C@H]2CC(=O)N(CCC#N)C(=O)N2	0.963414
iRNF130-5	CSC116288185	CCN(CC)C(=O)N1CCC(CC1)C2=NC(=NO2)C3C=CC=NC3=O	0.940859
iRNF130-6	CSC027259745	Br.C1CN2C(=CSC2=N1)C3=CC=CS3	0.910693
iRNF130-7	CSC027016462	NC(=O)CN1C=NC=2C(Br)=CC(Br)=CC2C1=O	0.924522
iRNF130-8	CSC050946457	CCN(C1CC1)C(=O)C=2C=C3C=CC=CC3=CC2O	0.939838
iRNF130-9	CSC005473254	NC(=O)CN(CC=1N=NSC1Cl)C2CCCC2	0.934476
iRNF130-10	CSC027016806	COC=1C=CC(NC(=O)[C@H](C)NC(=O)N)=CC1Cl	0.935626
iRNF130-11	CSC026133553	CC=1C=CC=C(NC(=O)C=2C=CC(F)=CC2F)C1C(=O)N3CCCC3	0.913604
iRNF130-12	CSC000695152	NS(=O)(=O)CC=1C=CC(Cl)=CC1Br	0.958216
iRNF130-13	CSC026421030	CCOC(=O)CNC(=O)[C@@H]1CCCC[C@H]1C(=O)NCC(=O)OCC	0.937247
iRNF130-14	CSC026225287	CN(CC(=O)NC1CC1)S(=O)(=O)C2=CC=C(Cl)S2	0.935377
iRNF130-15	CSC028301548	CC1CN(C)CCC1N(C)C(=O)NC=2C=CC=C3CCN(C(=O)C)C32	0.959149
iRNF130-16	CSC026948146	O=C(OC1CCCCC1=O)C2CCCN(C2)C(=O)N3CCCC3	0.91656
iRNF130-17	CSC028329293	COC[C@@H](O)CSC1=NN=C(O1)C2=CC=C(Cl)S2	0.906825
iRNF130-18	CSC028247587	Cl.CNCC(=O)NC1=NC=C(CC=2C=CC=C(Br)C2)S1	0.915304
iRNF130-19	CSC028262172	O=C(NC=1C=CC=C(C1)N2C(=O)CCNC2=O)C=3C=CN=CC3	0.907522
iRNF130-20	CSC025845312	CCN(CC1CCOC1)C(=O)NC2=NN=C(S2)C3CC3	0.921346
iRNF130-21	CSC027048354	CNC=1SN=C(C)C1C(=O)N2CCN(CC2)C3CC3	0.93481
iRNF130-22	CSC025905910	NC(=O)C1CCN(C1)C(=O)C=2NC=3C=CC(Cl)=CC3C2Cl	0.979589
iRNF130-23	CSC026577280	CCN(C1CC1)C(=O)CC2=CSC(=N2)N3CCNC3=O	0.949157
iRNF130-24	CSC027051961	CCC(=O)N1CCCC(C1)C(=O)C(C#N)C2=NC=3C=CC=CC3O2	0.907865
iRNF130-25	CSC026172021	CC(C)C(=O)NC1CCN(CC1)C=2C=CC=C(Cl)C2C#N	0.926205
iRNF130-26	CSC011440157	COC=1C=CC=C(C1)C(C)(C)C(=O)O	0.926998
iRNF130-27	CSC138471901	C[C@@H]1[C@@H](CCN1C=2C(C#N)=CC=CC2C#N)N3CCOCC3	0.908227
iRNF130-28	CSC138481699	CC1=NN(C)C(Cl)=C1C2CCCN2C(=O)C=3C=CNC3C	0.916622

iRNF130-29	CSC003364705	OC(=O)[C@H]1CCCN1C(=O)CC=2C=CC=CC2Cl	0.923714
iRNF130-30	CSC027664897	C[C@@H]1CN(CCN1C=2C(F)=CC(C#N)=CC2F)C3CCOCC3	0.91084
iRNF130-31	CSC001658573	CC(C)[C@H](NC(=O)C=1C=CC(Cl)=CC1Br)C(=O)O	0.943321
iRNF130-32	CSC116244659	CN1[C@H]2CCN(C[C@H]2NC1=O)C=3C(C#N)=CC=CC3C#N	0.924902
iRNF130-33	CSC000730120	NC1=NC=2CCSCC2S1	0.933829
iRNF130-34	CSC027557546	CS(=O)(=O)CC1=NC(=CS1)C=2C=CC(Cl)=C(Cl)C2	0.964299
iRNF130-35	CSC026426474	CS(=O)(=O)CCSC1=NN=C(CC=2C(F)=CC=CC2Cl)O1	0.969614
iRNF130-36	CSC027927782	CS(=O)(=O)CCNC=1C=CN=C2C=CC(Cl)=CC12	0.979682
iRNF130-37	CSC026485826	C1CC1C=2N=C3CCCC3=C(SC4=NN=C(S4)N5CCCC5)N2	0.921147
iRNF130-38	CSC047400665	CCN1CC[C@H]2OCCN([C@H]2C1)C(=O)C=3C(C)=NSC3NC4CC4	0.962463
iRNF130-39	CSC091063908	OC(=O)[C@H]1CC[C@H](CNS(=O)(=O)C=2C=CC=CC2Cl)CC1	0.917425
iRNF130-40	CSC133099212	O=C(N1CCCC1)C2=CC(=CN2)C(=O)N3CCC(=O)N4CCCC43	0.956596
iRNF130-41	CSC026251738	CCOC(=O)CC(O)C(=O)NC1=NC=2C=CC(OCC)=CC2S1	0.907779
iRNF130-42	CSC138472334	Cl.ClC=1C=CC(OCC2=NOC(=N2)C3CCNC3)=C(Cl)C1	0.923604
iRNF130-43	CSC000748996	OCCOC=1C(Cl)=CC(Cl)=CC1Cl	0.90701
iRNF130-44	CSC026832714	FC=1C=CC(=CC1)C=2N=NN(CC(=O)N(C3CC3)C4=CCCCC4)N2	0.923135
iRNF130-45	CSC028202369	CCCOC(=O)CC1N2C=NC=3C=CC=CC3C2=NNC1=O	0.935704
iRNF130-46	CSC026136920	CN1C=CSC1=NC(=O)CC=2C(F)=CC=CC2Cl	0.92655
iRNF130-47	CSC027592993	CCCN(C1CC1)C=2C=NN(C)C(=O)C2Cl	0.930918
iRNF130-48	CSC102901596	CCS(=O)(=O)CC1=NN=C(O1)C=2C=CC(Cl)=CC2Cl	0.963349
iRNF130-49	CSC138555307	ClC=1C=CC=C(C1Cl)N2C=C(CNS(=O)(=O)C3CC3)N=N2	0.971893
iRNF130-50	CSC026917642	CC1OC=2C=CC=CC2N(CCC(=O)N3CCCC3C(=O)O)C1=O	0.973929
iRNF130-51	CSC026445996	C[C@H](NS(=O)(=O)CC=1C=CC(F)=CC1C)C=2C=CC(Cl)=CN2	0.921103
iRNF130-52	CSC027388000	CCNC1=NN=C(SCC2=CSC(CC)=N2)S1	0.94425
iRNF130-53	CSC025996325	CCC=1ON=C(C)C1C(=O)N2CCC[C@H](CN3CCOCC3)C2	0.924901
iRNF130-54	CSC028347013	COC=1C=C(CC(C)NC=2C=CC(C)=C(NS(=O)(=O)C)C2)C=CN1	0.933109
iRNF130-55	CSC027527646	COC=1C(Cl)=CC(=CC1Cl)C(=O)N2CCC[C@@H]2C(=O)O	0.938295
iRNF130-56	CSC027633161	CNS(=O)(=O)C=1C=CC(=CN1)C(=O)N2CCC2C(C)C	0.924793
iRNF130-57	CSC133022628	CN1CCOCC1C=2C(C)=NN(CC3=NC=C(Cl)S3)C2C	0.925013
iRNF130-58	CSC138453579	CO[C@@H]1C[C@H](N(CC=2C=CC(F)=C(C#N)C2Cl)C1)C3=NC(C)=NN3	0.911173

<table>
<tr><td>iRNF130-59</td><td>CSC138468896</td><td>CN1N=CC=C1CN2C[C@@H](F)C[C@H]2CNC=3N=CN=C(N)C3Cl</td><td>0.918375</td></tr>
<tr><td>iRNF130-60</td><td>CSC138507995</td><td>NC[C@@H]1CCO[C@@H]1C2=NC(COC=3C=CC=C(Cl)C3Cl)=NO2</td><td>0.935445</td></tr>
<tr><td>iRNF130-61</td><td>CSC093238781</td><td>CC1=NN=C(NC(=O)[C@@H]2CCCO[C@H]2C=3C=NN(C)C3)N1C4CC4</td><td>0.932432</td></tr>
<tr><td>iRNF130-62</td><td>CSC133047083</td><td>COC=1C=CC(=CC1OC)C2=NOC(=N2)C3=CSC(CCN)=N3</td><td>0.917672</td></tr>
<tr><td>iRNF130-63</td><td>CSC138461036</td><td>CS(=O)(=O)C1=NN=C(CN2C3CCC2C=C(C3)C=4C=CC=CC4)S1</td><td>0.94284</td></tr>
<tr><td>iRNF130-64</td><td>CSC046486202</td><td>FC=1C(NC(=O)N2CN(CC3CC3)C(=O)C2)=CC=C4CNCCC14</td><td>0.914966</td></tr>
<tr><td>iRNF130-65</td><td>CSC105262941</td><td>CC1=NN=C2CN(C(CN12)C(=O)N)C(=O)C=3C=C(F)C=C(Cl)C3</td><td>0.907588</td></tr>
<tr><td>iRNF130-66</td><td>CSC133029451</td><td>FC=1C=C(C=CC1Cl)[C@@H]2C[C@H]2NS(=O)(=O)CCN3C=NN=N3</td><td>0.97345</td></tr>
<tr><td>iRNF130-67</td><td>CSC060886144</td><td>CCC1N(CCN(CC)C1=O)C(=O)CNC(=O)NC</td><td>0.927963</td></tr>
<tr><td>iRNF130-68</td><td>CSC133100711</td><td>OCC1CCN(C1)C2=NC=3C(F)=CC(Br)=CC3S2</td><td>0.948409</td></tr>
<tr><td>iRNF130-69</td><td>CSC116286215</td><td>CCN(CC)C(=O)N1CC2=NN(C)C=C2C1C(=O)OC</td><td>0.972135</td></tr>
<tr><td>iRNF130-70</td><td>CSC133102217</td><td>COC(C1CC1)C(=O)N2CC(=O)NC=3C=C(F)C(F)=CC23</td><td>0.914191</td></tr>
<tr><td>iRNF130-71</td><td>CSC059178943</td><td>CCC=1N=CN=C(N2CCC(C2)C(=O)N)C1F</td><td>0.938818</td></tr>
<tr><td>iRNF130-72</td><td>CSC073125466</td><td>COC=1C=C2CCN(CC=3C=C4CCCCN4N3)C(CC(=O)O)C2=CC1OC</td><td>0.914232</td></tr>
<tr><td>iRNF130-73</td><td>CSC133146861</td><td>COC(=O)C1=C(NC(=O)NC2CCCCC2C)N=C3CCCN13</td><td>0.916407</td></tr>
<tr><td>iRNF130-74</td><td>CSC133052583</td><td>COC=1C=CC=2C(=NC=NC2C1F)N3CC[C@H]([C@H]3C)N(C)CCO</td><td>0.922464</td></tr>
<tr><td>iRNF130-75</td><td>CSC138501138</td><td>CN(C)[C@@H]1CN(CC=2C=CC=C(C#N)C2F)C[C@@H]1N3C=CN=N3</td><td>0.956617</td></tr>
<tr><td>iRNF130-76</td><td>CSC046871874</td><td>FC=1C=C(C=C(F)C1N2CCOCC2)C3=NC(=NO3)C4CN5CCN4CC5</td><td>0.936864</td></tr>
<tr><td>iRNF130-77</td><td>CSC133144120</td><td>COC=1C=C(Br)C=C(CNC(=O)[C@@H]2C[C@@H]2C(=O)N)C1 |&1:12,14,r|</td><td>0.923758</td></tr>
<tr><td>iRNF130-78</td><td>CSC133035245</td><td>NCC1N(CCC=2C=CC=CC12)C(=O)[C@@H]3C[C@H]3C(=O)O |&1:14,16,r|</td><td>0.923943</td></tr>
<tr><td>iRNF130-79</td><td>CSC102895837</td><td>O=C(NS(=O)(=O)C=1C=CSC1)[C@@H]2C[C@H]2C3CCC3 |&1:11,13,r|</td><td>0.912382</td></tr>
<tr><td>iRNF130-80</td><td>CSC116286621</td><td>CC[C@@H]1OCC[C@H]1NC=2N=C(N)C(Br)=C(Cl)N2 |&1:2,6,r|</td><td>0.921413</td></tr>
<tr><td>iRNF130-81</td><td>CSC000601731</td><td>COC=1C=CC(Cl)=CC1C(=O)N2CCCC2C(=O)O</td><td>0.920031</td></tr>
<tr><td>iRNF130-82</td><td>CSC026394025</td><td>CNS(=O)(=O)C=1C=CC=C(CNC=2C=CC=C(F)C2C#N)C1</td><td>0.976423</td></tr>
<tr><td>iRNF130-83</td><td>CSC000749597</td><td>COC=1C=C(OC)C(=CC1Cl)[C@H](C)O</td><td>0.962979</td></tr>
<tr><td>iRNF130-84</td><td>CSC026808114</td><td>CCN(CC=1C=CC(Cl)=C(Cl)C1)C(=O)[C@@H]2CCCN2C(=O)N</td><td>0.973936</td></tr>
<tr><td>iRNF130-85</td><td>CSC028327045</td><td>CCCC1=NN=C(SC=2C=C(OCC)N=CN2)O1</td><td>0.917031</td></tr>
<tr><td>iRNF130-86</td><td>CSC025728025</td><td>Cl.FC(F)(F)C1=NC(=NO1)C2CCCNC2</td><td>0.917127</td></tr>
</table>

Supplementary Tab 11. Ranking result of TransformerCPI2.0 and other tools screening the same compound library against RNF130.

Method	iRNF130-63 ranking
**TransformerCPI2.0**	3,510/981,244, Top 0.4%
**TransformerCPI**	65,987/981,244, Top 6.7%
**GraphDTA**	189,247/981,244, Top 19.3%
**GCN**	384,498/981,244, Top 39.2%

Supplementary Tab 12. Rabeprazole target prediction.

Rank	Gene	Uniprot ID	Description	Score
1	ITPR1	Q14643	Inositol 1,4,5-trisphosphate receptor type 1	0.998982
2	FBP1	P09467	Fructose-1,6-bisphosphatase 1	0.998867
3	ACACB	O00763	Acetyl-CoA carboxylase 2	0.998795
4	ITPR3	Q14573	Inositol 1,4,5-trisphosphate receptor type 3	0.998475
5	ITPR2	Q14571	Inositol 1,4,5-trisphosphate receptor type 2	0.998127
6	ABCA1	O95477	ATP-binding cassette sub-family A member 1	0.997757
7	ABCC8	Q09428	ATP-binding cassette sub-family C member 8	0.995798
8	CHAT	P28329	Choline O-acetyltransferase	0.994322
9	POLE	Q07864	DNA polymerase epsilon catalytic subunit A	0.994208
10	ARL2	P36404	ADP-ribosylation factor-like protein 2	0.993315
11	LRRK2	Q5S007	Leucine-rich repeat serine/threonine-protein kinase 2	0.989988
12	UTRN	P46939	Utrophin	0.98867
13	ARF1	P84077	ADP-ribosylation factor 1	0.988254
14	ABCC5	O15440	Multidrug resistance-associated protein 5	0.98686
15	C3	P01024	Complement C3	0.986474
16	HTT	P42858	Huntingtin	0.986395
17	EPRS	P07814	Bifunctional glutamate/proline--tRNA ligase	0.983021
18	EIF2AK4	Q9P2K8	eIF-2-alpha kinase GCN2	0.982954
19	ABCC9	O60706	ATP-binding cassette sub-family C member 9	0.982515
20	TAOK3	Q9H2K8	Serine/threonine-protein kinase TAO3	0.981002

Supplementary Tab 13. Lansoprazole target prediction.

Rank	Gene	Uniprot ID	Description	Score
1	FBP1	P09467	Fructose-1,6-bisphosphatase 1	0.999822
2	ITPR1	Q14643	Inositol 1,4,5-trisphosphate receptor type 1	0.998049
3	MAP3K1 9	Q56UN5	Mitogen-activated protein kinase kinase kinase 19	0.997066
4	ITPR2	Q14571	Inositol 1,4,5-trisphosphate receptor type 2	0.996896
5	ARL2	P36404	ADP-ribosylation factor-like protein 2	0.996651
6	ARF1	P84077	ADP-ribosylation factor 1	0.996253
7	ITPR3	Q14573	Inositol 1,4,5-trisphosphate receptor type 3	0.996161
8	LRRK2	Q5S007	Leucine-rich repeat serine/threonine-protein kinase 2	0.996034
9	ABCC8	Q09428	ATP-binding cassette sub-family C member 8	0.995469
10	ACACB	O00763	Acetyl-CoA carboxylase 2	0.995447
11	F8	P00451	Coagulation factor VIII	0.995162
12	ABCA1	O95477	ATP-binding cassette sub-family A member 1	0.994401
13	HDAC4	P56524	Histone deacetylase 4	0.994142
14	ABCC5	O15440	Multidrug resistance-associated protein 5	0.99354
15	ATM	Q13315	Serine-protein kinase ATM	0.991644
16	ALK	Q9UM73	ALK tyrosine kinase receptor	0.990803
17	NUCB1	Q02818	Nucleobindin-1	0.988412
18	HSD17B3	P37058	Testosterone 17-beta-dehydrogenase 3	0.988405
19	KCNH8	Q96L42	Potassium voltage-gated channel subfamily H member 8	0.988265
20	C4A	P0C0L4	Complement C4-A	0.988112

Supplementary Tab 14. Omeprazole target prediction.

**Rank**	**Gene**	**Uniprot ID**	**Description**	**Score**
1	PLD1	Q13393	Phospholipase D1	0.997618
2	FBP1	P09467	Fructose-1,6-bisphosphatase 1	0.997351
3	MAP3K19	Q56UN5	Mitogen-activated protein kinase kinase kinase 19	0.997184
4	DGKZ	Q13574	Diacylglycerol kinase zeta	0.996634
5	ITPR1	Q14643	Inositol 1,4,5-trisphosphate receptor type 1	0.996055
6	ROS1	P08922	Proto-oncogene tyrosine-protein kinase ROS	0.995868
7	ARF1	P84077	ADP-ribosylation factor 1	0.995269
8	ACACB	O00763	Acetyl-CoA carboxylase 2	0.994592
9	PIK3CD	O00329	Phosphatidylinositol 4,5-bisphosphate 3-kinase catalytic subunit delta isoform	0.994178
10	ALK	Q9UM73	ALK tyrosine kinase receptor	0.992792
11	ITPR3	Q14573	Inositol 1,4,5-trisphosphate receptor type 3	0.992778
12	ARL2	P36404	ADP-ribosylation factor-like protein 2	0.992654
13	MAST1	Q9Y2H9	Microtubule-associated serine/threonine-protein kinase 1	0.992632
14	MST1R	Q04912	Macrophage-stimulating protein receptor	0.9925
15	PDE10A	Q9Y233	cAMP and cAMP-inhibited cGMP 3',5'-cyclic phosphodiesterase 10A	0.991689
16	KDM5D	Q9BY66	Lysine-specific demethylase 5D	0.99144
17	MYLK	Q15746	Myosin light chain kinase, smooth muscle	0.991346
18	C4A	P0C0L4	Complement C4-A	0.990324
19	LIG1	P18858	DNA ligase 1	0.989881
20	GAK	O14976	Cyclin-G-associated kinase	0.989778

Supplementary Tab 15. Pantoprazole target prediction.

Rank	Gene	Uniprot ID	Description	Score
1	ITPR1	Q14643	Inositol 1,4,5-trisphosphate receptor type 1	0.996287
2	EPRS	P07814	Bifunctional glutamate/proline--tRNA ligase	0.994877
3	EDNRA	P25101	Endothelin-1 receptor	0.994638
4	ITPR2	Q14571	Inositol 1,4,5-trisphosphate receptor type 2	0.994003
5	STK10	O94804	Serine/threonine-protein kinase 10	0.993642
6	ALK	Q9UM73	ALK tyrosine kinase receptor	0.992905
7	GAK	O14976	Cyclin-G-associated kinase	0.992869
8	MAP3K11	Q16584	Mitogen-activated protein kinase kinase kinase 11	0.991866
9	KDM5D	Q9BY66	Lysine-specific demethylase 5D	0.99125
10	C3	P01024	Complement C3	0.989104
11	MAP3K9	P80192	Mitogen-activated protein kinase kinase kinase 9	0.988459
12	MAST1	Q9Y2H9	Microtubule-associated serine/threonine-protein kinase 1	0.987881
13	PLK2	Q9NYY3	Serine/threonine-protein kinase PLK2	0.987577
14	ABCC8	Q09428	ATP-binding cassette sub-family C member 8	0.987273
15	TYK2	P29597	Non-receptor tyrosine-protein kinase TYK2	0.986807
16	STK3	Q13188	Serine/threonine-protein kinase 3	0.986787
17	ROS1	P08922	Proto-oncogene tyrosine-protein kinase ROS	0.986765
18	DNMT1	P26358	DNA (cytosine-5)-methyltransferase 1	0.986613
19	ARF1	P84077	ADP-ribosylation factor 1	0.98628
20	C4A	P0C0L4	Complement C4-A	0.98627

Supplementary Tab 16. Tanimoto similarity between PPIs and three known ARF1 inhibitors calculated by ECFP fingerprints.

	AMF-26	Brefeldin A	LM-11
**Rabeprazole**	0.101	0.047	0.082
**Lansoprazole**	0.102	0.048	0.083
**Omeprazole**	0.102	0.048	0.071
**Pantoprazole**	0.108	0.045	0.067

Supplementary Tab 17. Prediction scores of PPIs with ARF1 predicted by TransformerCPI2.0 and baseline models.

Methods	Omeprazole	Rabeprazole	Lansoprazole	Pantoprazole
**TransformerCPI2.0**	0.995	0.988	0.996	0.986
**TransformerCPI**	0.936	0.964	0.929	0.952
**GraphDTA**	0.007	0.054	0.154	0.013
**GCN**	0.025	0.018	0.007	0.018

Supplementary Tab 18. Atomic features of TransformerCPI2.0.

**Atom type**	C,N,O,F,P,S,Cl,Br,I,other (one hot)
**Degree of atom**	0,1,2,3,4,5,6 (one hot)
**Formal charge**	0 or 1
**Number of radical electrons**	0 or 1
**Hybridization Type**	sp,sp2,sp3,sp3d,sp3d2,other (one hot)
**Aromatic**	0 or 1
**Number of hydrogen atoms attached**	0,1,2,3,4 (one hot)
**Chirality**	0(False) or 1(True)
**Configuration**	R,S (one hot)
